# A Python script to merge Sanger sequences

**DOI:** 10.7717/peerj.11354

**Published:** 2021-04-27

**Authors:** Cen Chen, Bingguo Lu, Xiaofang Huang, Chuyun Bi, Lili Zhao, Yunzhuo Hu, Xuanyang Chen, Shiqiang Lin, Kai Huang

**Affiliations:** 1 Clinical Center for Human Genomic Research, Union Hospital, Huazhong University of Science and Technology, Wuhan, China; 2 Department of Radiology, Union Hospital, Tongji Medical College, Huazhong University of Science and Technology, Wuhan, China; 3 Department of Cardiovascular Diseases, Union Hospital, Tongji Medical College, Huazhong University of Science and Technology, Wuhan, China; 4 Provincial University Key Laboratory of Cellular Stress Response and Metabolic Regulation, College of Life Science, Fujian Normal University, Fuzhou, China; 5 Key Laboratory of Crop Biotechnology, Fujian Agriculture and Forestry University, Fujian Province Universities, Fuzhou, China; 6 State Key Laboratory of Infectious Disease Prevention and Control, Collaborative Innovation Center for Diagnosis and Treatment of Infectious Diseases, National Institute for Communicable Disease Control and Prevention, Chinese Center for Disease Control, Beijing, China

**Keywords:** Sanger sequencing, Merge, Python script

## Abstract

Merging Sanger sequences is frequently needed during the gene cloning process. In this study, we provide a Python script that is able to assemble multiple overlapping Sanger sequences. The script utilizes the overlapping regions within the tandem Sanger sequences to merge the Sanger sequences. The results demonstrate that the script can produce the merged sequence from the input Sanger sequences in a single run. The script offers a simple and free method for merging Sanger sequences and is useful for gene cloning.

## Introduction

Gene cloning is customarily required to study the gene functions in vivo and in vitro. For the most part, the gene of concern is ligated to a vector with a canonical method of restriction enzyme cutting and ligation, or the new-fashioned technique of seamless ligation (Okegawa & Motohashi, 2015). To ensure that the gene sequence is correct within the constructed plasmid, the Sanger sequencing is utilized to sequence the gene, and the results are aligned and checked (Zimmermann et al., 1988).

Sanger sequencing is a DNA sequencing technology by incorporating chain-terminating dideoxynucleotides, which are fluorescently labeled and can be read-out by automated sequencing machinery. Sanger sequencing has been widely applied to determine the DNA sequence since it was reported first time by Sanger et al. (1977) and Sanger, Nicklen & Coulson (1977). Though the next-generation sequencing has started a new era, Sanger sequencing is still one of the most popular methods due to its reliability, affordability, and feasibility (Schuster, 2008). However, the number of nucleotides determined by one Sanger sequencing reaction is around 1,000. For those genes with lengths of only several hundred nucleotides, it is possible to go through each one of the whole sequences with one single Sanger sequencing reaction, and the results can be directly aligned to the correct gene files. However, in other cases, the lengths of the genes exceed one thousand nucleotides, beyond the scope of one single Sanger sequencing reaction. To get the full-length sequence of a target gene, it is necessary to carry out DNA walking sequencing using a new primer based on the previous sequencing result. To sequence a large gene, several reactions might be required in both forward and reverse directions. These results have to be aligned to the correct gene file for confirmation.

It is preferred to merge all the walking results before alignment with the correct gene file, rather than aligning each walking result with the target gene file separately (Tang et al., 2020a, 2020b), especially for those large genes, which may be over 10,000 bps in length. To merge the multiple walking results, several commercial software can be applied, such as DNASTAR (DNASTAR, Inc., Madison, WI, USA), DNAMAN (Lynnon Biosoft, San Ramon, CA, USA), Vector NTI (Thermo Fisher Scientific Inc., Denver, CO, USA), and SnapGene (GSL Biotech LLC., San Diego, CA, USA), which are robust, however, expensive. For protein science and molecular biology, sequencing is mostly used to confirm whether the subcloning or mutagenesis is correct. Thereby, some functions of these commercial software are not essential, and the price of these software might be too high for the routine work of molecular cloning.

In terms of free software, there is an online tool that is able to merge overlapping long sequence fragments based on the program merger in the “EMBOSS” suite (Bell & Kramvis, 2013; Rice, Longden & Bleasby, 2000). Nevertheless, the web tool relies on web access and server status. “Cap3” is a free and effective program for merging DNA fragments, however, the command-line is complicated for users who are not familiar with commands of “Cap3” (Huang & Madan, 1999). “Staden”, containing a graphic user interface, provides a free and powerful package for analyzing giant genome sequencing by combining the “SPIN” and “EMBOSS” (Staden, Judge & Bonfield, 2003), again, this package is complicated for some users.

Since the first trial of the assembly of DNA sequence by computer, the algorithms have developed rapidly, which can be categorized into three classes: the first is overlap-layout-consensus method (Batzoglou, 2005), which applies an overlap graph; the second is de Bruijn graph roads (Idury & Waterman, 1995; Pevzner, 1989; Pevzner, Tang & Waterman, 2001), which uses a k-mer graph; and the last is greedy algorithm, which uses a k-mer or an overlap graph (Pop, 2009; Pop & Salzberg, 2008). Most of these algorithms are coded by C, which is challenging for biologists with less programming backgrounds.

Here, we introduce a Python script capable of merging multiple overlapping Sanger sequencing files by employing the alignment module of Biopython (Cock et al., 2009). To visualize the critical parameters of the merging process, we print the output of alignment and show the critical junction within the merged sequence. The script is useful for merging the Sanger sequences and increasing the efficiency of subcloning.

## Methods

### Running environments and input files

**Figure 1:**
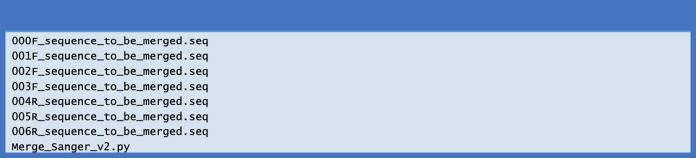
The test files used for running the script.

To run the script “Merge_Sanger_v2.py”, the macOS Catalina 10.15.3 or higher (Apple Inc., Cupertino, CA, USA) is needed. Python 3.7.3 (https://www.python.org/), Biopython 1.7.4, and “EMBOSS” 6.6.0 are necessary for running the script in this study (Cock et al., 2009; Rice, Longden & Bleasby, 2000). The users need to install the above three packages before running the script. Here are the sequencing result files of the pET28a-SV40NLS-hCas9-SV40NLS, which is originated from PX458 (Ran et al., 2013) (Supplemental Information S1) (Fig. 1). All input files are in the seq format. Of note, the first four characters of the input file name must consist of three numbers (000, 001, 002, 003, …) plus one capital letter F or R (F for forward Sanger sequencing and R for reverse Sanger sequencing). Adding extra four characters to the original filename provided by the sequencing company is suggested. The numbers in the filenames should be ordered from small to big along with the 5′ to 3′ of the sense strand.

### Running script

Please follow the steps below to run the script.

1. Make a new directory and copy the sequencing result files of the above section (Running environments and input files) to the newly made directory. Also, copy the script “Merge_Sanger_v2.py” (Supplemental Information S2) to the directory.
2. Open a new terminal and “cd” to the directory. Run the following command. Notice that a space between every two items is needed and the backward slash is used for multiline command input.
  python3.7 Merge_Sanger_v2.py 001F_sequence_to_be_merged.seq \
  002F_sequence_to_be_merged.seq 003F_sequence_to_be_merged.seq \
  004R_sequence_to_be_merged.seq 005R_sequence_to_be_merged.seq \
  006R_sequence_to_be_merged.seq
  The Sanger sequencing result files must be input from lower to higher according to the first three numbers of the file name. It is convenient to input the first three numbers and then use the tab key to fill the rest of the file name automatically. Our script is able to merge dozens of (up to 1,000) Sanger sequencing result files at a time, which is far enough for usual needs in labs.
3. After running finishes, a folder named “merged_sequence”, which contains the merged sequence file with the name “merged.seq”, appears.

### Manual validation of the merged Sanger sequences

First, all the Sanger sequence files are renamed and changed to fasta format with TextEdit. Those Sanger sequence files in the reverse sequencing direction are transformed to the reverse complement sequences with “EMBOSS” command “revseq”. All the Sanger sequence files are now in the forward sequencing direction in fasta format. Then, the “EMBOSS” “needle” is utilized to align each pair of tandem sequence files, and the command “less” is used to open the result files of alignments. The final merged sequence file is generated by combining the Sanger sequences based on the overlapping regions. The manually combined sequence file is used to align with the script’s merged sequence, and the time consumed by the manual method is compared with that used by the script.

### Comparison with CAP3 and Fragment Merger for merging Sanger sequences

“CAP3” is a free and powerful software for contig assembly during shotgun sequencing of genome projects (Huang & Madan, 1999). It is also incorporated in commercial software to assemble Sanger sequencing results, for example, SnapGene (GSL Biotech LLC., San Diego, CA, USA). For comparison, the on-line version of the “CAP3” program at the website (http://doua.prabi.fr/software/cap3) is used. With regard to the example input files, we first try to assemble only two tandem Sanger sequences, then three tandem Sanger sequences, and so on. We also compared “Fragment Merger” (Bell & Kramvis, 2013) at the website (http://hvdr.bioinf.wits.ac.za/fmt/) using the example Sanger files. The example files in seq format were changed to FASTA format and then running of the “Fragment Merger” is carried out following the instructions on the webpage using default parameters. Similarly, we start from assembly of only two tandem Sanger sequences, then three tandem Sanger sequences, and so on.

### Algorithm

**Figure 2:**
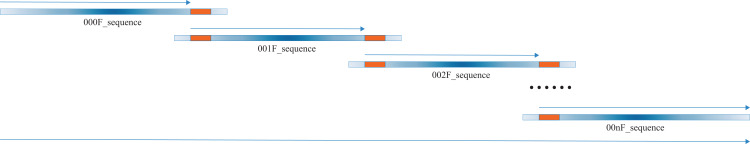
A diagrammatic view of merging multiple Sanger sequences. The thick blue lines show the multiple (no less than two) Sanger sequences to be merged, with the color depth indicating the sequencing quality, which is high in the middle part and low in both ends. The thick orange lines represent the first line of full consensus (50 bps long) appearing in the needle output file for the two consecutive Sanger sequences. The thin blue arrow above the thick blue line indicates the part to be extracted for joining to the full-length sequence, which is shown by the long thin blue arrow at the bottom. All the Sanger sequences are preprocessed to 5′–3′ direction, aligned and merged, thus the direction of all thin arrows is 5′–3′.

Our script heavily relies on excellent pre-existing packages, i.e., “EMBOSS” (Rice, Longden & Bleasby, 2000) and Biopython (Cock et al., 2009), which are versatile and extremely useful to molecular biologists world around. The script begins to transform the input Sanger sequence files, which might be in forward or reverse sequencing directions, into the new Sanger sequence files in forward sequencing direction. Our algorithm takes advantage of the overlapping regions within the tandem Sanger sequences, which are commonly no less than 200 bps (Fig. 2). The alignment module of the Biopython is called to conduct the needle alignment of the two consecutive Sanger sequence files. The script then parses the output file of needle alignment to seek for the first 100% identity alignment fragment that is no less than 50 bps and records the critical fragments of the corresponding Sanger sequences. These critical fragments are then used to extract the bases from each Sanger sequence for merging to the full-length sequence.

## Results

**Figure 3:**
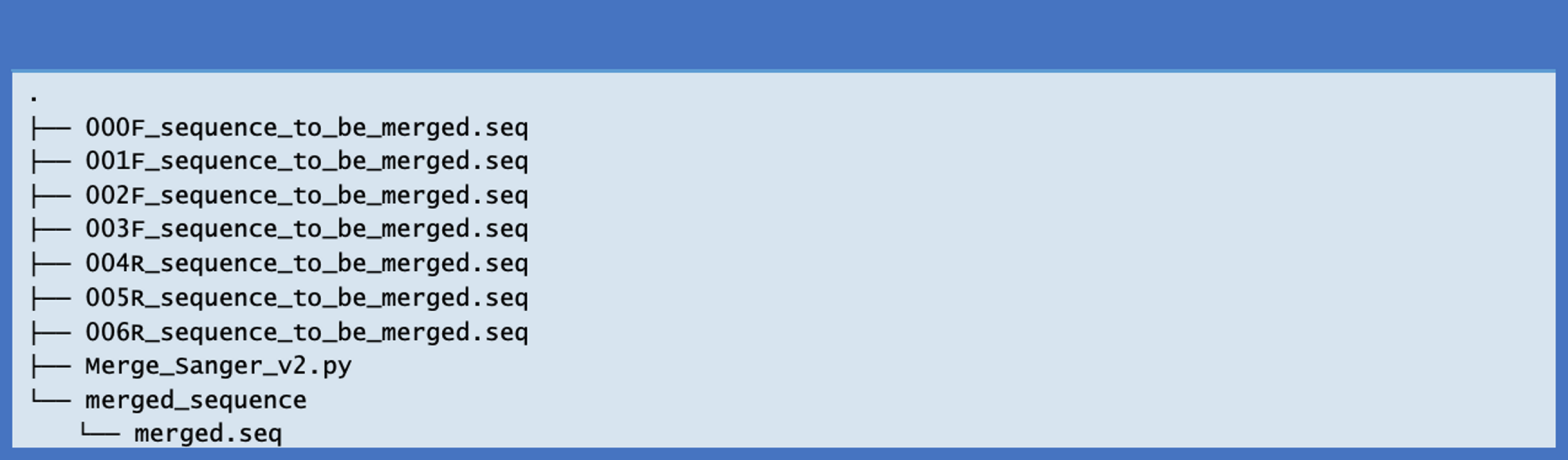
The directory showing the output file after merging the Sanger sequences.

When the script finishes one run, the merged Sanger sequence “merged.seq” is stored in a new folder named “merged_sequence” (Fig. 3).

**Figure 4:**
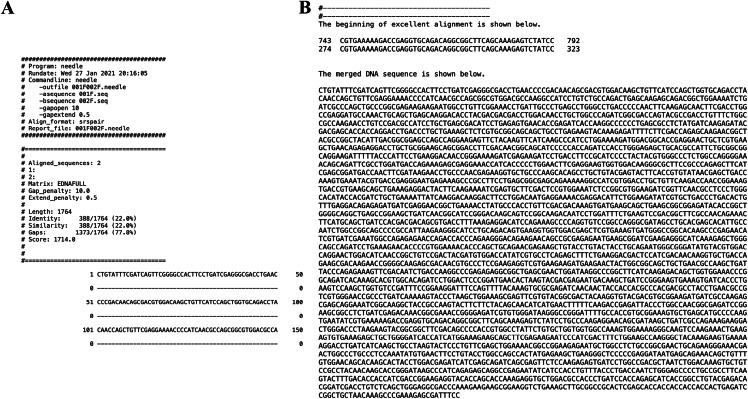
The screenshots of the script run showing the partial output of the needle alignment (A) and the first consensus line and the merged DNA sequence (B).

Besides, there are outputs on the screen showing the needle alignment and the critical junction of the consensus region between the two adjacent Sanger sequences (Fig. 4), which clarifies the working process of merging. Further, the merged sequence is shown on the screen. The results showed that the running process of the script is fast, and the output is clear.

Then, we conducted a manual operation of merging the Sanger sequences (Supplemental Information S3). The manual method is labor-intensive and tedious. It took about one hour to carry out the manual merging of the seven Sanger sequencing files, whereas the script accomplishes the same job within seconds after command input. Theoretically, our script works in the same way as the manual operation, showing that our script conforms to molecular biologists’ intuition and experience when merging the Sanger sequences. Actually, our script prints the working process including the results of each “EMBOSS” “needle” alignment and the boundary used for fragment joining so that the user knows what’s going on during the Sanger sequence merging (Supplemental Information S4). Moreover, the script works as accurately as the manual operation and saves time in the Sanger sequence merging. Besides, the result generated manually (Supplemental Information S3) is the same as that obtained using the script.

We also compared the script with “CAP3”, a free, well-known, and widely-used software for contig assembly. First, using the test input files, we tried the script to assemble two, three, four, and up to seven tandemly arranged Sanger sequences. As was expected, the script worked well in all the above situations. However, when we tried merging the same example Sanger sequences with the online version of “CAP3” (Huang & Madan, 1999), out of expectation, “CAP3” could not merge several two tandem Sanger sequences, e.g., 000F_sequence_to_be_merged.seq and 001F_sequence_to_be_merged.seq could not be merged by “CAP3”. Nevertheless, the quality of 000F_sequence_to_be_merged.seq and 001F_sequence_to_be_merged.seq fulfilled the requirement for Sanger sequences merging. It seems that “CAP3” is not completely well suited to merging the example Sanger sequences in its default parameters.

When using the online “Fragment Merger” to assemble the example Sanger sequences (Supplemental Information S5), we found that the assembled sequence was not the same as that obtained by manual operation. This is probably because the fact that “Fragment Merger” uses the “EMBOSS” “merger”, in which the issue of the disputed bases needs to be solved by reading the corresponding peaks in the chromatogram files.

In comparison, our script can deal with all tested situations since the script adopts the same logic as the manual operation when merging the Sanger sequences, which agrees with the inherent characteristics of Sanger sequencing (sequencing quality from low to high to low in a reaction) and the reality of experimental operations.

**Figure 5:**
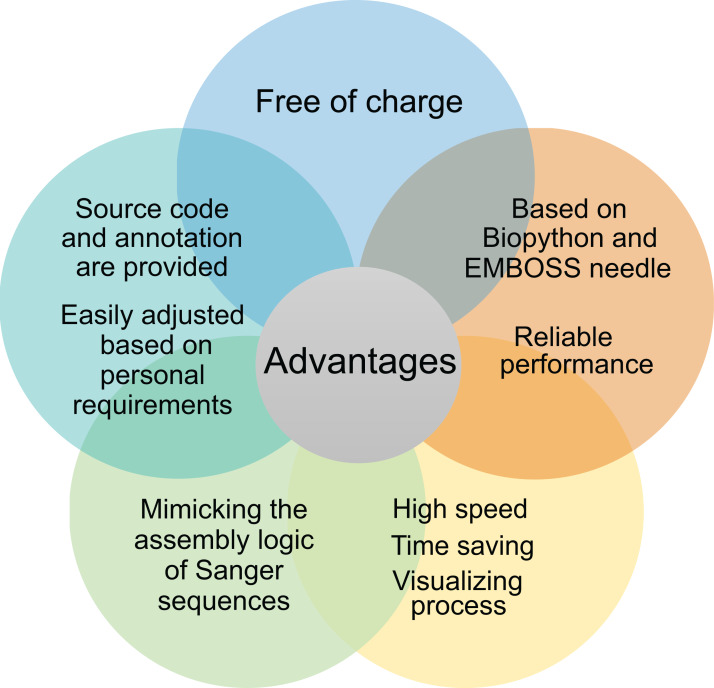
Advantages of the script.

Notably, the commercial solutions, such as DNASTAR, DNAMAN, Vector NTI, and SnapGene, cost hundreds to thousands of dollars’ license fees per year, which is expensive for many small groups. In contrast, our script is open source and free of charge, and fulfills the basic requirements of molecular cloning (Bi et al., 2020). Moreover, compared to the black box of commercial software, we provide the source code, written in Python language that resembles natural languages and annotated in detail, which is relatively easy to learn and allows the users to tailor the script according to their needs. Together, the advantages of our script are free of charge, open-source, reliable, and high speed (Fig. 5).

## Discussion

Merging Sanger sequences is common and necessary work during gene cloning. The “EMBOSS” command “merger” is capable of generating a merged sequenced based on the global alignment of Needleman and Wunsch (Rice, Longden & Bleasby, 2000). To merge two overlapping Sanger sequences, the global alignment might bring in mismatches due to the decrease of signal-to-noise ratio during the later stage of the Sanger sequencing reaction and gel running. According to the local sequence quality score, the “EMBOSS” “merger” deals with the disputed bases, which is rather complicated and often brings in the unwanted bases.

To address this issue, we look for the first full consensus line within the output of needle alignment and pick up the fragments to join the merged sequence according to the variations of the signal-to-noise ratio of the Sanger sequencing reaction, which avoids dealing with the dispute settlement for the mismatch bases. Besides, using “EMBOSS” “needle”, our algorithm can align tandemly arranged Sanger sequences and calculate the boundary of sequences to merge, potentially minimizing the influence of repeated sequences utmostly.

Interestingly, while we are trying to simplify the assembly of Sanger sequences using Python, Chao et al. reported an automated R package, “sangeranalyseR”, for analyzing the Sanger sequencing data (Chao et al., 2020; Hill et al., 2014). The “sangeranalyseR” is an interactive program based on the R/bioconductor that focuses on analyzing Sanger sequence data in ABI format (Gentleman et al., 2004). Together with the program reported in this manuscript, it reflects that the researchers require various programs based on different coding languages for merging DNA fragments since each researcher has specific background and requirements.

## Conclusions

In sum, we provide an efficient and straightforward method to merge multiple Sanger sequences using Python, which can be run at a local computer and fulfills the basic requirements of subcloning and protein science. Our script mimics the manual assembly of the Sanger sequences like a molecular biologist, which is in line with the researchers’ experience and intuition in the fields of gene and protein.

## Supplemental Information

10.7717/peerj.11354/supp-1 Supplemental Information 1 Sanger sequencing file 000F.

10.7717/peerj.11354/supp-2 Supplemental Information 2 Sanger sequencing file 001F.

10.7717/peerj.11354/supp-3 Supplemental Information 3 Sanger sequencing file 002F.

10.7717/peerj.11354/supp-4 Supplemental Information 4 Sanger sequencing file 003F.

10.7717/peerj.11354/supp-5 Supplemental Information 5 Sanger sequencing file 004R.

10.7717/peerj.11354/supp-6 Supplemental Information 6 Sanger sequencing file 005R.

10.7717/peerj.11354/supp-7 Supplemental Information 7 Sanger sequencing file 006R.

10.7717/peerj.11354/supp-8 Supplemental Information 8 The Python script of this study.

10.7717/peerj.11354/supp-9 Supplemental Information 9 Manual validation of the merged Sanger sequences.

10.7717/peerj.11354/supp-10 Supplemental Information 10 Screen output of the script run.

10.7717/peerj.11354/supp-11 Supplemental Information 11 Comparison with Fragment Merger for Sanger sequence merging.

10.7717/peerj.11354/supp-12 Supplemental Information 12 Readme for the script of this study.

## Additional Information and Declarations

### Data Availability

The following information was supplied regarding data availability: The example input files, the Python script, the readme file, the manual validation of the merging of the example input files, the screen output of the script run, and the comparison with Fragment Merger for Sanger sequence merging are available in the Supplemental Files.
