# Supplementary material for: A Python script to merge Sanger sequences
Source: PeerJ. 2021 Apr 27;9:e11354. doi: 10.7717/peerj.11354 (PMC8086567; doi:10.7717/peerj.11354)
Supplement: Supplemental Information 9 [file peerj-09-11354-s009.pdf]

1. The original Sanger sequencing file in seq format is changed to fasta format with TextEdit. The forward Sanger sequencing files are stored as F000~003.fasta and the reverse Sanger sequencing files are stored as R004~006.fasta, which are shown below:

```
>F000
GCTGACTCTTCCCTCTAGAATAATTTTGTTTAACTTTAAGAAGGAGATATACCATGGGCAGCAGCCATCATCATCATCATCACAGCAGCGGCCTGG
AAGTTCTGTTCCAGGGGCCCCATATGGCTAGCATGACTGGTGGACAGCAAATGGGTCGCGGATCCCCAAAGAAGAAGCGGAAGGTCGGTATCCACG
GAGTCCCAGCAGCCGACAAGAAGTACAGCATCGGCCTGGACATCGGCACCAACTCTGTGGGCTGGGCCGTGATCACCGACGAGTACAAGGTGCCCA
GCAAGAAATTCAAGGTGCTGGGCAACACCGACCGGCACAGCATCAAGAAGAACCTGATCGGAGCCCTGCTGTTCGACAGCGGCGAAACAGCCGAGG
CCACCCGGCTGAAGAGAACCGCCAGAAGAAGATACACCAGACGGAAGAACCGGATCTGCTATCTGCAAGAGATCTTCAGCAACGAGATGGCCAAGG
TGGACGACAGCTTCTTCCACAGACTGGAAGAGTCCTTCCTGGTGGAAGAGGATAAGAAGCACGAGCGGCACCCCATCTTCGGCAACATCGTGGACG
AGGTGGCCTACCACGAGAAGTACCCCACCATCTACCACCTGAGAAAGAAACTGGTGGACAGCACCGACAAGGCCGACCTGCGGCTGATCTATCTGG
CCCTGGCCCACATGATCAAGTTCCGGGGCCACTTCCTGATCGAGGGCGACCTGAACCCCGACAACAGCGACGTGGACAAGCTGTTCATCCAGCTGG
TGCAGACCTACAACCAGCTGTTCGAGGAAAACCCCATCAACGCCAGCGGCGTGGACGCCAAGGCCATCCTGTCTGCCAGACTGAGCAAGAGCAGAC
GGCTGGAAAATCTGATCGCCCAGCTGCCCGGCGAGAAGAAGAATGGCCCTGTTCGGAAACCTGATTGCCCTGAGCCTGGGCCTGACCCCCAACTTC
AAGAGCAACTTCGACCTGGCCGAGGATGCCAAACTGCAGCTGAGCAAGGACACCTACGACGACGACCTGACACCTGCTGGCCCAGATCGGCGACCA
GTACGCCGACCTGTTTTCTATGCAGACTGTCGACGCATCTGCTGACGACATCTGAGAGTGAACACGAGATCACAGCTCCCTTGACCTTATGATCAG
AGAATATCGATCGAGCACCTATCGAGACTGAACTCCTGACTGGA

>F001
CTGTATTTCGATCAGTTCGGGGCCACTTCCTGATCGAGGGCGACCTGAACCCCGACAACAGCGACGTGGACAAGCTGTTCATCCAGCTGGTGCAGA
CCTACAACCAGCTGTTCGAGGAAAACCCCATCAACGCCAGCGGCGTGGACGCCAAGGCCATCCTGTCTGCCAGACTGAGCAAGAGCAGACGGCTGG
AAAATCTGATCGCCCAGCTGCCCGGCGAGAAGAAGAATGGCCTGTTCGGAAACCTGATTGCCCTGAGCCTGGGCCTGACCCCCAACTTCAAGAGCA
ACTTCGACCTGGCCGAGGATGCCAAACTGCAGCTGAGCAAGGACACCTACGACGACGACCTGGACAACCTGCTGGCCCAGATCGGCGACCAGTACG
CCGACCTGTTTCTGGCCGCCAAGAACCTGTCCGACGCCATCCTGCTGAGCGACATCCTGAGAGTGAACACCGAGATCACCAAGGCCCCCCTGAGCG
CCTCTATGATCAAGAGATACGACGAGCACCACCAGGACCTGACCCTGCTGAAAGCTCTCGTGCGGCAGCAGCTGCCTGAGAAGTACAAAGAGATTT
TCTTCGACCAGAGCAAGAACGGCTACGCCGGCTACATTGACGGCGGAGCCAGCCAGGAAGAGTTCTACAAGTTCATCAAGCCCATCCTGGAAAAGA
TGGACGGCACCGAGGAACTGCTCGTGAAGCTGAACAGAGAGGACCTGCTGCGGAAGCAGCGGACCTTCGACAACGGCAGCATCCCCCACCAGATCC
ACCTGGGAGAGCTGCACGCCATTCTGCGGCGGCAGGAAGATTTTTACCCATTCCTGAAGGACAACCGGGAAAAGATCGAGAAGATCCTGACCTTCC
GCATCCCCTACTACGTGGGCCCTCTGGGCCAGGGGAAACAGCAGATTCGCCTGGATGACCAGAAGAGCGAGGAAACCATCACCCCCTGCACTTCGA
GGAAGTGTGGACAGGCGCTCGCCAGAGCTCATCGAGCGATGACACTCGATAGACTGCCAACGAGAGGTGCTGCCAGCACAGCTGCTGTA

>F002
GGGATCATCTGGAAGTGGACGGCACCGAGGAACTGCTCGTGAAGCTGAACAGAGAGGACCTGCTGCGGAAGCAGCGGACCTTCGACAACGGCAGCA
TCCCCCACCAGATCCACCTGGGAGAGCTGCACGCCATTCTGCGGCGGCAGGAAGATTTTTACCCATTCCTGAAGGACAACCGGGAAAAGATCGAGA
AGATCCTGACCTTCCGCATCCCCTACTACGTGGGCCCTCTGGCCAGGGGAAACAGCAGATTCGCCTGGATGACCAGAAAGAGCGAGGAAACCATCA
CCCCCTGGAACTTCGAGGAAGTGGTGGACAAGGGCGCTTCCGCCCAGAGCTTCATCGAGCGGATGACCAACTTCGATAAGAACCTGCCCAACGAGA
AGGTGCTGCCCAAGCACAGCCTGCTGTACGAGTACTTCACCGTGTATAACGAGCTGACCAAAGTGAAATACGTGACCGAGGGAATGAGAAAGCCCG
CCTTCCTGAGCGGCGAGCAGAAAAAGGCCATCGTGGACCTGCTGTTCAAGACCAACCGGAAAGTGACCGTGAAGCAGCTGAAAGAGGACTACTTCA
AGAAAATCGAGTGCTTCGACTCCGTGGAAATCTCCGGCGTGGAAGATCGGTTCAACGCCTCCCTGGGCACATACCACGATCTGCTGAAAATTATCA
AGGACAAGGACTTCCTGGACAATGAGGAAAACGAGGACATTCTGGAAGATATCGTGCTGACCCTGACACTGTTTGAGGACAGAGAGATGATCGAGG
AACGGCTGAAAACCTATGCCCACCTGTTCGACGACAAAGTGATGAAGCAGCTGAAGCGGCGGAGATACACCGGCTGGGGCAGGCTGAGCCGGAAGC
TGATCAACGGCATCCGGGACAAGCAGTCCGGGCAGACATCCTGGATTTCCTGAAGTCCGACGGCTTCGCAACAGAAACTCATGCAGCTGATCACGA
CGACAGCTGACTTAAGAGACATCAGATGCCAGTGTCGCAGGCGATAGCTGCACGAGCAATGGCATCTGTGCAGGCCGCATAGAGCATCTGCGACTG
AGTGTGACAGCTCTGAATGATGACCGCCAAGCCGAAAACTCTGGTATTCG

>F003
GGCCAGGCTACACGATCTGCTGAATTATCAAGGACAAGGACTTCCTGGACAATGAGGAAAACGAGGACATTCTGGAAGATATCGTGCTGACCCTGA
CACTGTTTGAGGACAGAGAGATGATCGAGGAACGGCTGAAAACCTATGCCCACCTGTTCGACGACAAAGTGATGAAGCAGCTGAAGCGGCGGAGAT
ACACCGGCTGGGGCAGGCTGAGCCGGAAGCTGATCAACGGCATCCGGGACAAGCAGTCCGGCAAGACAATCCTGGATTTCCTGAAGTCCGACGGCT
TCGCCAACAGAAACTTCATGCAGCTGATCCACGACGACAGCCTGACCTTTAAAGAGGACATCCAGAAAGCCCAGGTGTCCGGCCAGGGCGATAGCC
TGCACGAGCACATTGCCAATCTGGCCGGCAGCCCCGCCATTAAGAAGGGCATCCTGCAGACAGTGAAGGTGGTGGACGAGCTCGTGAAAGTGATGG
GCCGGCACAAGCCCGAGAACATCGTGATCGAAATGGCCAGAGAGAACCAGACCACCCAGAAGGGACAGAAGAACAGCCGCGAGAGAATGAAGCGGA
TCGAAGAGGGCATCAAAGAGCTGGGCAGCCAGATCCTGAAAGAACACCCCGTGGAAAACACCCAGCTGCAGAACGAGAAGCTGTACCTGTACTACC
TGCAGAATGGGCGGGATATGTACGTGGACCAGGAACTGGACATCAACCGGCTGTCCGACTACGATGTGGACCATATCGTGCCTCAGAGCTTTCTGA
AGGACGACTCCATCGACAACAAAGTGCTGACCAGAAGCGACAAGAACCGGGGCAAGAGCGACAACGTGCCCTCCGAAGAGGTCGTGAAGAAGATGA
AGAACTACTGGCGGCAGCTGCTGAACGCCCAAGCTGATTACCCAGAGAAAGTTCGACATCTGACCAACGCCGAGAGAGGCGGCCTGAGCGAACTGG
ATAATGCGCTTCATCAGGAGACAGCTGATGAACCCCGCAGATCACAAAGCACGTGGCACAGATCTGACTCCCGGATGACACTTAGTACGACGAGAT
GACAGCCTGATCGGCAGTGAAGTGATCACCCTTGAGTTCAGCTTGGGTTCCGATTCGCAAAGAATTCCTAGCTTACCAAATTGC

>R004
TCGGTTCTTCGGATCGGACACCAGCTTGGACTTCAGGGTGATCACTTTCACTTCCCGGATCAGCTTGTCATTCTCGTCGTACTTAGTGTTCATCCG
GGAGTCCAGGATCTGTGCCACGTGCTTTGTGATCTGCCGGGTTTCCACCAGCTGTCTCTTGATGAAGCCGGCCTTATCCAGTTCGCTCAGGCCGCC
TCTCTCGGCCTTGGTCAGATTGTCGAACTTTCTCTGGGTAATCAGCTTGGCGTTCAGCAGCTGCCGCCAGTAGTTCTTCATCTTCTTCACGACCTC
TTCGGAGGGCACGTTGTCGCTCTTGCCCCGGTTCTTGTCGCTTCTGGTCAGCACCTTGTTGTCGATGGAGTCGTCCTTCAGAAAGCTCTGAGGCAC
GATATGGTCCACATCGTAGTCGGACAGCCGGTTGATGTCCAGTTCCTGGTCCACGTACATATCCCGCCCATTCTGCAGGTAGTACAGGTACAGCTT
CTCGTTCTGCAGCTGGGTGTTTTCCACGGGGTGTTCTTTCAGGATCTGGCTGCCCAGCTCTTTGATGCCCTCTTCGATCCGCTTCATTCTCTCGCG
GCTGTTCTTCTGTCCCTTCTGGGTGGTCTGGTTCTCTCTGGCCATTTCGATCACGATGTTCTCGGGCTTGTGCCGGCCCATCACTTTCACGAGCTC
GTCCACCACCTTCACTGTCTGCAGGATGCCCTTCTTAATGGCGGGGCTGCCGGCCAGATTGGCAATGTGCTCGTGCAGGCTATCGCCCTGGCCGGA
CACCTGGGGCTTTCTGGATGTCCTCTTTAAAGGTCACGCTGTCGTCGTGGATCAGCTGCATGAAGTTTCTGTTGGCGAAGCCGTCGGACTTCAGAA
ATCCAGGATTGTCTTGCCGGACTGCTTGTCCCGGATGCCGTTGATCAGCTTCCGGCTCAGCCTGCCCCAGCCGGTGTATCTCCGCCGCTTCAGCTG
CTTCATCACTTTGTCGTCGAACAGGTGGGGCATAGGTTTTCAGCCGTTCTCGATCATCTCTCTGTCCTCAAACAGTGTCAGGTCAGCACGATATCT
TTCAGATGTCTCGTTTTCTCATTGGTCAAGCAGTCATGTCCTGATTGCTTCAGCAGAATCGTGATGTGTGCCCAGGAGGCGTTGAATCAGACCATC

>R005
```

```
CCTCACTGTGCTTGGCTTCAGAAGTCGATGGGATTCTTCTCGAAGCTGCTTCTTTCCATGATGGTGATCCCCAGCAGCTCTTTCACACTCTTCAGT
TTCTTGGACTTGCCCTTTTCCACTTTGGCCACCACCAGCACAGAATAGGCCACGGTGGGGCTGTCGAAGCCGCCGTACTTCTTAGGGTCCCAGTCC
TTCTTTCTGGCGATCAGCTTATCGCTGTTCCTCTTGGGCAGGATAGACTCTTTGCTGAAGCCGCCTGTCTGCACCTCGGTCTTTTTCACGATATTC
ACTTGGGGCATGCTCAGCACTTTCCGCACGGTGGCAAAATCCCGGCCCTTATCCCACACGATCTCCCCGGTTTCGCCGTTTGTCTCGATCAGAGGC
CGCTTCCGGATCTCGCCGTTGGCCAGGGTAATCTCGGTCTTGAAAAAGTTCATGATGTTGCTGTAGAAGAAGTACTTGGCGGTAGCCTTGCCGATT
TCCTGCTCGCTCTTGGCGATCATCTTCCGCACGTCGTACACCTTGTAGTCGCCGTACACGAACTCGCTTTCCAGCTTAGGGTACTTTTTGATCAGG
GCGGTTCCCACGACGGCGTTCAGGTAGGCGTCGTGGGCGTGGTGGTAGTTGTTGATCTCGCGCACTTTGTAAAACTGGAAATCCTTCCGGAAATCG
GACACCAGCTTGGACTTCAGGGTGATCACTTTCACTTCCCGGATCAGCTTGTCATTCTCGTCGTACTTAGTGTTCATCCGGGAGTCCAGGATCTGT
GCCACGTGCTTTGTGATCTGCCGGGTTTCCACCAGCTGTCTCTTGATGAAGCCGGGCCTTATCCAGTTCGCTCAGCCGCCTCTCTCGGGCCTTGGT
CAGATTGTCGAACTTTCTCTGGGTAATCAGCTTGGCGTTCAGCAGCTGCCGCCAGTAGTTCTTCATCTTCTCACGACTCTTCGGAGGGCACGTGTC
GCTCTGCCCCGGTCTGTCGCTTCTGGTCAGCACCTTGTGTCGATGGAGTCGTCCTTCAGAAAGC

>R006
GGAAATCGCTCTTTCGGGCTTTGTTAGCAGCCGGATCTCAGTGGTGGTGGTGGTGGTGCTCGAGTGCGGCCGCAAGCTTTCAGACCTTCCGCTTCT
TCTTTGGGTCGCCTCCCAGCTGAGACAGGTCGATCCGTGTCTCGTACAGGCCGGTGATGCTCTGGTGGATCAGGGTGGCGTCCAGCACCTCTTTGG
TGCTGGTGTACCTCTTCCGGTCGATGGTGGTGTCAAAGTACTTGAAGGCGGCAGGGGCTCCCAGATTGGTCAGGGTAAACAGGTGGATGATATTCT
CGGCCTGCTCTCTGATGGGCTTATCCCGGTGCTTGTTGTAGGCGGACAGCACTTTGTCCAGATTAGCGTCGGCCAGGATCACTCTCTTGGAGAACT
CGCTGATCTGCTCGATGATCTCGTCCAGGTAGTGCTTGTGCTGTTCCACAAACAGCTGTTTCTGCTCATTATCCTCGGGGGAGCCCTTCAGCTTCT
CATAGTGGCTGGCCAGGTACAGGAAGTTCACATATTTGGAGGGCAGGGCCAGTTCGTTTCCCTTCTGCAGTTCGCCGGCAGAGGCCAGCATTCTCT
TCCGGCCGTTTTCCAGCTCGAACAGGGAGTACTTAGGCAGCTTGATGATCAGGTCCTTTTTCACTTCTTTGTAGCCCTTGGCTTCCAGAAAGTCGA
TGGGATTCTTCTCGAAGCTGCTTCTTTCCATGATGGTGATCCCCAGCAGCTCTTTCACACTCTTCAGTTTCTTGGACTTGCCCTTTTCCACTTTGG
CCACCACCAGCACAGAATAGGCCACGGTGGGGCTGTCGAAGCCGCCGTACTTCTTAGGGTCCCAGTCCTTCTTTCTGGCGATCAGCTTATCGCTGT
TCCTCTTGGGCAGGATAGACTCTTTGCTGAAGCCGCCTGTCTGCACCTCGGTCTTTTTCACGATATTCACTTGGGGCATGCTCAGCACTTTCCGCA
CGGTGCAAAATCCCGGCTCTTATCCCACACGATCTCCCGGGTTTCGCCGATGTCTCGATCAGAGGCCGCTTTCTGGATCTCGCTCGTTGGCAGGGT
AATCTCGTCTTGAAAAAGTTCATGATGGTGGCTGTAGAAGAAGTACTTGGCGTAGCTTGGCGGATTTCTGGCTTCGCTCTTGCCGATCATCTTCGT
ACGTCGTACTCTTGTAGTCCGTCGTAACTCGAAACTTCGCCTTCAGC
```

2. The reverse Sanger sequencing files are changed to the corresponding forward Sanger sequencing files with EMBOSS revseq, which are stored as R004~006rev.fasta.

```
>R004rev
GATGGTCTGATTCAACGCCTCCTGGGCACACATCACGATTCTGCTGAAGCAATCAGGACATGACTGCTTGACCAATGAGAAAACGAGACATCTGAA
AGATATCGTGCTGACCTGACACTGTTTGAGGACAGAGAGATGATCGAGAACGGCTGAAAACCTATGCCCCACCTGTTCGACGACAAAGTGATGAAG
CAGCTGAAGCGGCGGAGATACACCGGCTGGGGCAGGCTGAGCCGGAAGCTGATCAACGGCATCCGGGACAAGCAGTCCGGCAAGACAATCCTGGAT
TTCTGAAGTCCGACGGCTTCGCCAACAGAAACTTCATGCAGCTGATCCACGACGACAGCGTGACCTTTAAAGAGGACATCCAGAAAGCCCCAGGTG
TCCGGCCAGGGCGATAGCCTGCACGAGCACATTGCCAATCTGGCCGGCAGCCCCGCCATTAAGAAGGGCATCCTGCAGACAGTGAAGGTGGTGGAC
GAGCTCGTGAAAGTGATGGGCCGGCACAAGCCCGAGAACATCGTGATCGAAATGGCCAGAGAGAACCAGACCACCCAGAAGGGACAGAAGAACAGC
CGCGAGAGAATGAAGCGGATCGAAGAGGGCATCAAAGAGCTGGGCAGCCAGATCCTGAAAGAACACCCCGTGGAAAACACCCAGCTGCAGAACGAG
AAGCTGTACCTGTACTACCTGCAGAATGGGCGGGATATGTACGTGGACCAGGAACTGGACATCAACCGGCTGTCCGACTACGATGTGGACCATATC
GTGCCTCAGAGCTTTCTGAAGGACGACTCCATCGACAACAAGGTGCTGACCAGAAGCGACAAGAACCGGGGCAAGAGCGACAACGTGCCCTCCGAA
GAGGTCGTGAAGAAGATGAAGAACTACTGGCGGCAGCTGCTGAACGCCAAGCTGATTACCCAGAGAAAGTTCGACAATCTGACCAAGGCCGAGAGA
GGCGGCCTGAGCGAACTGGATAAGGCCGGCTTCATCAAGAGACAGCTGGTGGAAACCCGGCAGATCACAAAGCACGTGGCACAGATCCTGGACTCC
CGGATGAACACTAAGTACGACGAGAATGACAAGCTGATCCGGGAAGTGAAAGTGATCACCCTGAAGTCCAAGCTGGTGTCCGATCCGAAGAACCGA

>R005rev
GCTTTCTGAAGGACGACTCCATCGACACAAGGTGCTGACCAGAAGCGACAGACCGGGGCAGAGCGACACGTGCCCTCCGAAGAGTCGTGAGAAGAT
GAAGAACTACTGGCGGCAGCTGCTGAACGCCAAGCTGATTACCCAGAGAAAGTTCGACAATCTGACCAAGGCCCGAGAGAGGCGGCTGAGCGAACT
GGATAAGGCCCGGCTTCATCAAGAGACAGCTGGTGGAAACCCGGCAGATCACAAAGCACGTGGCACAGATCCTGGACTCCCGGATGAACACTAAGT
ACGACGAGAATGACAAGCTGATCCGGGAAGTGAAAGTGATCACCCTGAAGTCCAAGCTGGTGTCCGATTTCCGGAAGGATTTCCAGTTTTACAAAG
TGCGCGAGATCAACAACTACCACCACGCCCACGACGCCTACCTGAACGCCGTCGTGGGAACCGCCCTGATCAAAAAGTACCCTAAGCTGGAAAGCG
AGTTCGTGTACGGCGACTACAAGGTGTACGACGTGCGGAAGATGATCGCCAAGAGCGAGCAGGAAATCGGCAAGGCTACCGCCAAGTACTTCTTCT
ACAGCAACATCATGAACTTTTTCAAGACCGAGATTACCCTGGCCAACGGCGAGATCCGGAAGCGGCCTCTGATCGAGACAAACGGCGAAACCGGGG
AGATCGTGTGGGATAAGGGCCGGGATTTTGCCACCGTGCGGAAAGTGCTGAGCATGCCCCAAGTGAATATCGTGAAAAAGACCGAGGTGCAGACAG
GCGGCTTCAGCAAAGAGTCTATCCTGCCCAAGAGGAACAGCGATAAGCTGATCGCCAGAAAGAAGGACTGGGACCCTAAGAAGTACGGCGGCTTCG
ACAGCCCCACCGTGGCCTATTCTGTGCTGGTGGTGGCCAAAGTGGAAAAGGGCAAGTCCAAGAAACTGAAGAGTGTGAAAGAGCTGCTGGGGATCA
CCATCATGGAAAGAAGCAGCTTCGAGAAGAATCCCATCGACTTCTGAAGCCAAGCACAGTGAGG

>R006rev
GCTGAAGGCGAAGTTTCGAGTTACGACGGACTACAAGAGTACGACGTACGAAGATGATCGGCAAGAGCGAAGCCAGAAATCCGCCAAGCTACGCCA
AGTACTTCTTCTACAGCCACCATCATGAACTTTTTCAAGACGAGATTACCCTGCCAACGAGCGAGATCCAGAAAGCGGCCTCTGATCGAGACATCG
GCGAAACCCGGGAGATCGTGTGGGATAAGAGCCGGGATTTTGCACCGTGCGGAAAGTGCTGAGCATGCCCCAAGTGAATATCGTGAAAAAGACCGA
GGTGCAGACAGGCGGCTTCAGCAAAGAGTCTATCCTGCCCAAGAGGAACAGCGATAAGCTGATCGCCAGAAAGAAGGACTGGGACCCTAAGAAGTA
CGGCGGCTTCGACAGCCCCACCGTGGCCTATTCTGTGCTGGTGGTGGCCAAAGTGGAAAAGGGCAAGTCCAAGAAACTGAAGAGTGTGAAAGAGCT
GCTGGGGATCACCATCATGGAAAGAAGCAGCTTCGAGAAGAATCCCATCGACTTTCTGGAAGCCAAGGGCTACAAAGAAGTGAAAAAGGACCTGAT
CATCAAGCTGCCTAAGTACTCCCTGTTCGAGCTGGAAAACGGCCGGAAGAGAATGCTGGCCTCTGCCGGCGAACTGCAGAAGGGAAACGAACTGGC
CCTGCCCTCCAAATATGTGAACTTCCTGTACCTGGCCAGCCACTATGAGAAGCTGAAGGGCTCCCCCGAGGATAATGAGCAGAAACAGCTGTTTGT
GGAACAGCACAAGCACTACCTGGACGAGATCATCGAGCAGATCAGCGAGTTCTCCAAGAGAGTGATCCTGGCCGACGCTAATCTGGACAAAGTGCT
GTCCGCCTACAACAAGCACCGGGATAAGCCCATCAGAGAGCAGGCCGAGAATATCATCCACCTGTTTACCCTGACCAATCTGGGAGCCCCTGCCGC
CTTCAAGTACTTTGACACCACCATCGACCGGAAGAGGTACACCAGCACCAAAGAGGTGCTGGACGCCACCCTGATCCACCAGAGCATCACCGGCCT
GTACGAGACACGGATCGACCTGTCTCAGCTGGGAGGCGACCCAAAGAAGAAGCGGAAGGTCTGAAAGCTTGCGGCCGCACTCGAGCACCACCACCA
CCACCACTGAGATCCGGCTGCTAACAAAGCCCGAAAGAGCGATTTCC
```

3. EMBOSS needle is used to align the tandemly arranged Sanger sequencing files and the results are shown below. The blue highlight shows the first time that the two sequences match for 50 continuous nucleotides. The numbers in red

highlight indicate the positions of interval for joining to the final merged sequence.

```
F000.fasta-F001.fasta
F000     1 GCTGACTCTTCCCTCTAGAATAATTTTGTTTAACTTTAAGAAGGAGATAT    50

F001     0 --------------------------------------------------     0

F000    51 ACCATGGGCAGCAGCCATCATCATCATCATCACAGCAGCGGCCTGGAAGT   100

F001     0 --------------------------------------------------     0

F000   101 TCTGTTCCAGGGGCCCCATATGGCTAGCATGACTGGTGGACAGCAAATGG   150

F001     0 --------------------------------------------------     0

F000   151 GTCGCGGATCCCCAAAGAAGAAGCGGAAGGTCGGTATCCACGGAGTCCCA   200

F001     0 --------------------------------------------------     0

F000   201 GCAGCCGACAAGAAGTACAGCATCGGCCTGGACATCGGCACCAACTCTGT   250

F001     0 --------------------------------------------------     0

F000   251 GGGCTGGGCCGTGATCACCGACGAGTACAAGGTGCCCAGCAAGAAATTCA   300

F001     0 --------------------------------------------------     0

F000   301 AGGTGCTGGGCAACACCGACCGGCACAGCATCAAGAAGAACCTGATCGGA   350

F001     0 --------------------------------------------------     0

F000   351 GCCCTGCTGTTCGACAGCGGCGAAACAGCCGAGGCCACCCGGCTGAAGAG   400

F001     0 --------------------------------------------------     0

F000   401 AACCGCCAGAAGAAGATACACCAGACGGAAGAACCGGATCTGCTATCTGC   450

F001     0 --------------------------------------------------     0

F000   451 AAGAGATCTTCAGCAACGAGATGGCCAAGGTGGACGACAGCTTCTTCCAC   500

F001     0 --------------------------------------------------     0

F000   501 AGACTGGAAGAGTCCTTCCTGGTGGAAGAGGATAAGAAGCACGAGCGGCA   550

F001     0 --------------------------------------------------     0

F000   551 CCCCATCTTCGGCAACATCGTGGACGAGGTGGCCTACCACGAGAAGTACC   600

F001     0 --------------------------------------------------     0

F000   601 CCACCATCTACCACCTGAGAAAGAAACTGGTGGACAGCACCGACAAGGCC   650

F001     0 --------------------------------------------------     0

F000   651 GACCTGCGGCTGATCTATCTGGCCCTGGCCCACATGATCAAGTTCCGGGG   700
                    |||   |||.|              .|||| |||| |||||
F001     1 ---------CTG---TATTT--------------CGATC-AGTT-CGGGG    22

F000   701 CCACTTCCTGATCGAGGGCGACCTGAACCCCGACAACAGCGACGTGGACA   750
           ||||||||||||||||||||||||||||||||||||||||||||||||||
F001    23 CCACTTCCTGATCGAGGGCGACCTGAACCCCGACAACAGCGACGTGGACA    72

F000   751 AGCTGTTCATCCAGCTGGTGCAGACCTACAACCAGCTGTTCGAGGAAAAC   800
           ||||||||||||||||||||||||||||||||||||||||||||||||||
F001    73 AGCTGTTCATCCAGCTGGTGCAGACCTACAACCAGCTGTTCGAGGAAAAC   122

F000   801 CCCATCAACGCCAGCGGCGTGGACGCCAAGGCCATCCTGTCTGCCAGACT   850
           ||||||||||||||||||||||||||||||||||||||||||||||||||
F001   123 CCCATCAACGCCAGCGGCGTGGACGCCAAGGCCATCCTGTCTGCCAGACT   172

F000   851 GAGCAAGAGCAGACGGCTGGAAAATCTGATCGCCCAGCTGCCCGGCGAGA   900
           ||||||||||||||||||||||||||||||||||||||||||||||||||
F001   173 GAGCAAGAGCAGACGGCTGGAAAATCTGATCGCCCAGCTGCCCGGCGAGA   222
```

```
F000                 901 AGAAGAATGGCCCTGTTCGGAAACCTGATTGCCCTGAGCCTGGGCCTGAC    950
                         |||||||||| |||||||||||||||||||||||||||||||||||||||
F001                 223 AGAAGAATGG-CCTGTTCGGAAACCTGATTGCCCTGAGCCTGGGCCTGAC    271

F000                 951 CCCCAACTTCAAGAGCAACTTCGACCTGGCCGAGGATGCCAAACTGCAGC   1000
                         ||||||||||||||||||||||||||||||||||||||||||||||||||
F001                 272 CCCCAACTTCAAGAGCAACTTCGACCTGGCCGAGGATGCCAAACTGCAGC    321

F000                1001 TGAGCAAGGACACCTACGACGACGACCT-GAC-ACCTGCTGGCCCAGATC   1048
                         |||||||||||||||||||||||||||| ||| |||||||||||||||||
F001                 322 TGAGCAAGGACACCTACGACGACGACCTGGACAACCTGCTGGCCCAGATC    371

F000                1049 GGCGACCAGTACGCCGACCTGTTTTCTATGC-----AGA--CTGT-CGAC   1090
                         ||||||||||||||||||||| ||||| .||     |||  |||| ||||
F001                 372 GGCGACCAGTACGCCGACCTG-TTTCT-GGCCGCCAAGAACCTGTCCGAC    419

F000                1091 G-CAT-CTGCTGA-CGACAT-CTGAGAGTGAACA-CGAGATCAC---AGC   1132
                         | ||| ||||||| |||||| ||||||||||||| |||||||||   .||
F001                 420 GCCATCCTGCTGAGCGACATCCTGAGAGTGAACACCGAGATCACCAAGGC    469

F000                1133 TCCCTTGA---CCT-TATGATCAGAGA-ATATCGATCGAGCACCTATCGA   1177
                         .|||.|||   ||| |||||||| ||| ||| ||| |||||||| |.|..
F001                 470 CCCCCTGAGCGCCTCTATGATCA-AGAGATA-CGA-CGAGCACC-ACCAG    515

F000                1196 --------------------------------------------------   1196

F001                 566 CAAAGAGATTTTCTTCGACCAGAGCAAGAACGGCTACGCCGGCTACATTG    615

F000                1196 --------------------------------------------------   1196

F001                 616 ACGGCGGAGCCAGCCAGGAAGAGTTCTACAAGTTCATCAAGCCCATCCTG    665

F000                1196 --------------------------------------------------   1196

F001                 666 GAAAAGATGGACGGCACCGAGGAACTGCTCGTGAAGCTGAACAGAGAGGA    715

F000                1196 --------------------------------------------------   1196

F001                 716 CCTGCTGCGGAAGCAGCGGACCTTCGACAACGGCAGCATCCCCCACCAGA    765

F000                1196 --------------------------------------------------   1196

F001                 766 TCCACCTGGGAGAGCTGCACGCCATTCTGCGGCGGCAGGAAGATTTTTAC    815

F000                1196 --------------------------------------------------   1196

F001                 816 CCATTCCTGAAGGACAACCGGGAAAAGATCGAGAAGATCCTGACCTTCCG    865

F000                1196 --------------------------------------------------   1196

F001                 866 CATCCCCTACTACGTGGGCCCTCTGGCCAGGGGAAACAGCAGATTCGCC    915

F000                1196 --------------------------------------------------   1196

F001                 916 TGGATGACCAGAAGAGCGAGGAAACCATCACCCCCTGCACTTCGAGGAAG    965

F000                1196 --------------------------------------------------   1196

F001                 966 TGTGGACAGGCGCTCGCCAGAGCTCATCGAGCGATGACACTCGATAGACT   1015

F000                1196 ----------------------------------   1196

F001                1016 GCCAACGAGAGGTGCTGCCAGCACAGCTGCTGTA   1049

F001.fasta-F002.fasta
F001                   1 CTGTATTTCGATCAGTTCGGGGCCACTTCCTGATCGAGGGCGACCTGAAC     50

F002                   0 --------------------------------------------------      0

F001                  51 CCCGACAACAGCGACGTGGACAAGCTGTTCATCCAGCTGGTGCAGACCTA    100

F002                   0 --------------------------------------------------      0

F001                 101 CAACCAGCTGTTCGAGGAAAACCCCATCAACGCCAGCGGCGTGGACGCCA    150
```

```
F002                    0 --------------------------------------------------      0

F001                  151 AGGCCATCCTGTCTGCCAGACTGAGCAAGAGCAGACGGCTGGAAAATCTG    200

F002                    0 --------------------------------------------------      0

F001                  201 ATCGCCCAGCTGCCCGGCGAGAAGAAGAATGGCCTGTTCGGAAACCTGAT    250

F002                    0 --------------------------------------------------      0

F001                  251 TGCCCTGAGCCTGGGCCTGACCCCCAACTTCAAGAGCAACTTCGACCTGG    300

F002                    0 --------------------------------------------------      0

F001                  301 CCGAGGATGCCAAACTGCAGCTGAGCAAGGACACCTACGACGACGACCTG    350

F002                    0 --------------------------------------------------      0

F001                  351 GACAACCTGCTGGCCCAGATCGGCGACCAGTACGCCGACCTGTTTCTGGC    400

F002                    0 --------------------------------------------------      0

F001                  401 CGCCAAGAACCTGTCCGACGCCATCCTGCTGAGCGACATCCTGAGAGTGA    450

F002                    0 --------------------------------------------------      0

F001                  451 ACACCGAGATCACCAAGGCCCCCCTGAGCGCCTCTATGATCAAGAGATAC    500

F002                    0 --------------------------------------------------      0

F001                  501 GACGAGCACCACCAGGACCTGACCCTGCTGAAAGCTCTCGTGCGGCAGCA    550

F002                    0 --------------------------------------------------      0

F001                  551 GCTGCCTGAGAAGTACAAAGAGATTTTCTTCGACCAGAGCAAGAACGGCT    600

F002                    0 --------------------------------------------------      0

F001                  601 ACGCCGGCTACATTGACGGCGGAGCCAGCCAGGAAGAGTTCTACAAGTTC    650
                                                         ||             |.||
F002                    1 -------------------------------GG-------------GATC      6

F001                  651 ATCAAGCCCATCCTGGAAAAGATGGACGGCACCGAGGAACTGCTCGTGAA    700
                          ||          ||||||   .||||||||||||||||||||||||||||
F002                    7 AT----------CTGGAA---GTGGACGGCACCGAGGAACTGCTCGTGAA     43

F001                  701 GCTGAACAGAGAGGACCTGCTGCGGAAGCAGCGGACCTTCGACAACGGCA    750
                          ||||||||||||||||||||||||||||||||||||||||||||||||||
F002                   44 GCTGAACAGAGAGGACCTGCTGCGGAAGCAGCGGACCTTCGACAACGGCA     93

F001                  751 GCATCCCCCACCAGATCCACCTGGGAGAGCTGCACGCCATTCTGCGGCGG    800
                          ||||||||||||||||||||||||||||||||||||||||||||||||||
F002                   94 GCATCCCCCACCAGATCCACCTGGGAGAGCTGCACGCCATTCTGCGGCGG    143

F001                  801 CAGGAAGATTTTTACCCATTCCTGAAGGACAACCGGGAAAAGATCGAGAA    850
                          ||||||||||||||||||||||||||||||||||||||||||||||||||
F002                  144 CAGGAAGATTTTTACCCATTCCTGAAGGACAACCGGGAAAAGATCGAGAA    193

F001                  851 GATCCTGACCTTCCGCATCCCCTACTACGTGGGCCCTCTGGGCCAGGGGA    900
                          ||||||||||||||||||||||||||||||||||||||| ||||||||||
F002                  194 GATCCTGACCTTCCGCATCCCCTACTACGTGGGCCCTCT-GGCCAGGGGA    242

F001                  901 AACAGCAGATTCGCCTGGATGACCAG-AAGAGCGAGGAAACCATCACCCC    949
                          |||||||||||||||||||||||||| |||||||||||||||||||||||
F002                  243 AACAGCAGATTCGCCTGGATGACCAGAAAGAGCGAGGAAACCATCACCCC    292

F001                  950 CT-GCACTTCGAGGAAGT-GTGGACA--GGCGCT--CG-CCAGAGC-TCA    991
                          || |.||||||||||||| |||||||  ||||||  || ||||||| |||
F002                  293 CTGGAACTTCGAGGAAGTGGTGGACAAGGGCGCTTCCGCCCAGAGCTTCA    342

F001                  992 TCGAGC-GATGAC--AC-TCGAT-AGA--CTG-CCAACGAG-AGGTGCTG   1032
                          |||||| ||||||  || ||||| |||  ||| |||||||| ||||||||
F002                  343 TCGAGCGGATGACCAACTTCGATAAGAACCTGCCCAACGAGAAGGTGCTG    392
```

```
F001              1033 CC--AGCACAG-CTGCTGTA------------------------------   1049
                       ||  ||||||| ||||||||
F002               393 CCCAAGCACAGCCTGCTGTACGAGTACTTCACCGTGTATAACGAGCTGAC    442

F001              1049 --------------------------------------------------   1049

F002               443 CAAAGTGAAATACGTGACCGAGGGAATGAGAAAGCCCGCCTTCCTGAGCG    492

F001              1049 --------------------------------------------------   1049

F002               493 GCGAGCAGAAAAAGGCCATCGTGGACCTGCTGTTCAAGACCAACCGGAAA    542

F001              1049 --------------------------------------------------   1049

F002               543 GTGACCGTGAAGCAGCTGAAAGAGGACTACTTCAAGAAAATCGAGTGCTT    592

F001              1049 --------------------------------------------------   1049

F002               593 CGACTCCGTGGAAATCTCCGGCGTGGAAGATCGGTTCAACGCCTCCCTGG    642

F001              1049 --------------------------------------------------   1049

F002               643 GCACATACCACGATCTGCTGAAAATTATCAAGGACAAGGACTTCCTGGAC    692

F001              1049 --------------------------------------------------   1049

F002               693 AATGAGGAAAACGAGGACATTCTGGAAGATATCGTGCTGACCCTGACACT    742

F001              1049 --------------------------------------------------   1049

F002               743 GTTTGAGGACAGAGAGATGATCGAGGAACGGCTGAAAACCTATGCCCACC    792

F001              1049 --------------------------------------------------   1049

F002               793 TGTTCGACGACAAAGTGATGAAGCAGCTGAAGCGGCGGAGATACACCGGC    842

F001              1049 --------------------------------------------------   1049

F002               843 TGGGGCAGGCTGAGCCGGAAGCTGATCAACGGCATCCGGGACAAGCAGTC    892

F001              1049 --------------------------------------------------   1049

F002               893 CGGGCAGACATCCTGGATTTCCTGAAGTCCGACGGCTTCGCAACAGAAAC    942

F001              1049 --------------------------------------------------   1049

F002               943 TCATGCAGCTGATCACGACGACAGCTGACTTAAGAGACATCAGATGCCAG    992

F001              1049 --------------------------------------------------   1049

F002               993 TGTCGCAGGCGATAGCTGCACGAGCAATGGCATCTGTGCAGGCCGCATAG   1042

F001              1049 --------------------------------------------------   1049

F002              1043 AGCATCTGCGACTGAGTGTGACAGCTCTGAATGATGACCGCCAAGCCGAA   1092

F001              1049 --------------   1049

F002              1093 AACTCTGGTATTCG   1106

F002.fasta-F003.fasta
F002                 1 GGGATCATCTGGAAGTGGACGGCACCGAGGAACTGCTCGTGAAGCTGAAC     50

F003                 0 --------------------------------------------------      0

F002                51 AGAGAGGACCTGCTGCGGAAGCAGCGGACCTTCGACAACGGCAGCATCCC    100

F003                 0 --------------------------------------------------      0

F002               101 CCACCAGATCCACCTGGGAGAGCTGCACGCCATTCTGCGGCGGCAGGAAG    150

F003                 0 --------------------------------------------------      0

F002               151 ATTTTTACCCATTCCTGAAGGACAACCGGGAAAAGATCGAGAAGATCCTG    200
```

```
F003                     0 --------------------------------------------------      0

F002                   201 ACCTTCCGCATCCCCTACTACGTGGGCCCTCTGGCCAGGGGAAACAGCAG    250

F003                     0 --------------------------------------------------      0

F002                   251 ATTCGCCTGGATGACCAGAAAGAGCGAGGAAACCATCACCCCCTGGAACT    300

F003                     0 --------------------------------------------------      0

F002                   301 TCGAGGAAGTGGTGGACAAGGGCGCTTCCGCCCAGAGCTTCATCGAGCGG    350

F003                     0 --------------------------------------------------      0

F002                   351 ATGACCAACTTCGATAAGAACCTGCCCAACGAGAAGGTGCTGCCCAAGCA    400

F003                     0 --------------------------------------------------      0

F002                   401 CAGCCTGCTGTACGAGTACTTCACCGTGTATAACGAGCTGACCAAAGTGA    450

F003                     0 --------------------------------------------------      0

F002                   451 AATACGTGACCGAGGGAATGAGAAAGCCCGCCTTCCTGAGCGGCGAGCAG    500

F003                     0 --------------------------------------------------      0

F002                   501 AAAAAGGCCATCGTGGACCTGCTGTTCAAGACCAACCGGAAAGTGACCGT    550

F003                     0 --------------------------------------------------      0

F002                   551 GAAGCAGCTGAAAGAGGACTACTTCAAGAAAATCGAGTGCTTCGACTCCG    600

F003                     0 --------------------------------------------------      0

F002                   601 TGGAAATCTCCGGCGTGGAAGATCGGTTCAACGCCTCCCTGGGCACA---    647
                                                                    ||| ||
F003                     1 -----------------------------------------GGC-CAGGC      8

F002                   648 TACCACGATCTGCTGAAAATTATCAAGGACAAGGACTTCCTGGACAATGA    697
                           || ||||||||||||  |||||||||||||||||||||||||||||||||
F003                     9 TA-CACGATCTGCTG--AATTATCAAGGACAAGGACTTCCTGGACAATGA     55

F002                   698 GGAAAACGAGGACATTCTGGAAGATATCGTGCTGACCCTGACACTGTTTG    747
                           ||||||||||||||||||||||||||||||||||||||||||||||||||
F003                    56 GGAAAACGAGGACATTCTGGAAGATATCGTGCTGACCCTGACACTGTTTG    105

F002                   748 AGGACAGAGAGATGATCGAGGAACGGCTGAAAACCTATGCCCACCTGTTC    797
                           ||||||||||||||||||||||||||||||||||||||||||||||||||
F003                   106 AGGACAGAGAGATGATCGAGGAACGGCTGAAAACCTATGCCCACCTGTTC    155

F002                   798 GACGACAAAGTGATGAAGCAGCTGAAGCGGCGGAGATACACCGGCTGGGG    847
                           ||||||||||||||||||||||||||||||||||||||||||||||||||
F003                   156 GACGACAAAGTGATGAAGCAGCTGAAGCGGCGGAGATACACCGGCTGGGG    205

F002                   848 CAGGCTGAGCCGGAAGCTGATCAACGGCATCCGGGACAAGCAGTCCGGGC    897
                           ||||||||||||||||||||||||||||||||||||||||||||||||..
F003                   206 CAGGCTGAGCCGGAAGCTGATCAACGGCATCCGGGACAAGCAGTCCGGCA    255

F002                   898 AGAC-ATCCTGGATTTCCTGAAGTCCGACGGCTTCG-CAACAGAAAC-TC    944
                           |||| ||||||||||||||||||||||||||||||| |||||||||| ||
F003                   256 AGACAATCCTGGATTTCCTGAAGTCCGACGGCTTCGCCAACAGAAACTTC    305

F002                   945 ATGCAGCTGAT-CACGACGACAG-CTGAC---TTAAGA-GACATC---AG    985
                           ||||||||||| ||||||||||| |||||   |.|||| ||||||   |.
F003                   306 ATGCAGCTGATCCACGACGACAGCCTGACCTTTAAAGAGGACATCCAGAA    355

F002                   986 ATGCCA-GTGTC--GC--AGGCGATAG-CTGCACGAGCA-ATGGC--ATC   1026
                           |..||| |||||  ||  .|||||||| ||||||||||| ||.||  |||
F003                   356 AGCCCAGGTGTCCGGCCAGGGCGATAGCCTGCACGAGCACATTGCCAATC    405

F002                  1027 TGTGCAGGCCG------CAT-----AGAGCAT-CTGC-GAC--TGA----   1057
                           || ||.|||.|      |||     ||.|||| |||| |||  |||
F003                   406 TG-GCCGGCAGCCCCGCCATTAAGAAGGGCATCCTGCAGACAGTGAAGGT    454

F002                  1058 -GTGTGAC-AGCTC-TGAA--TGAT-GACCG--CCAAG-CCGAAAAC-TC   1097
```

```
                         ||| ||| ||||| ||||  |||| |.|||  .|||| ||||.||| ||
F003                455 GGTG-GACGAGCTCGTGAAAGTGATGGGCCGGCACAAGCCCGAGAACATC    503

F002               1098 TGGTATTCG-----------------------------------------   1106
                          ||..|||
F003                504 --GTGATCGAAATGGCCAGAGAGAACCAGACCACCCAGAAGGGACAGAAG    551

F002               1106 --------------------------------------------------   1106

F003                552 AACAGCCGCGAGAGAATGAAGCGGATCGAAGAGGGCATCAAAGAGCTGGG    601

F002               1106 --------------------------------------------------   1106

F003                602 CAGCCAGATCCTGAAAGAACACCCCGTGGAAAACACCCAGCTGCAGAACG    651

F002               1106 --------------------------------------------------   1106

F003                652 AGAAGCTGTACCTGTACTACCTGCAGAATGGGCGGGATATGTACGTGGAC    701

F002               1106 --------------------------------------------------   1106

F003                702 CAGGAACTGGACATCAACCGGCTGTCCGACTACGATGTGGACCATATCGT    751

F002               1106 --------------------------------------------------   1106

F003                752 GCCTCAGAGCTTTCTGAAGGACGACTCCATCGACAACAAAGTGCTGACCA    801

F002               1106 --------------------------------------------------   1106

F003                802 GAAGCGACAAGAACCGGGGCAAGAGCGACAACGTGCCCTCCGAAGAGGTC    851

F002               1106 --------------------------------------------------   1106

F003                852 GTGAAGAAGATGAAGAACTACTGGCGGCAGCTGCTGAACGCCCAAGCTGA    901

F002               1106 --------------------------------------------------   1106

F003                902 TTACCCAGAGAAAGTTCGACATCTGACCAACGCCGAGAGAGGCGGCCTGA    951

F002               1106 --------------------------------------------------   1106

F003                952 GCGAACTGGATAATGCGCTTCATCAGGAGACAGCTGATGAACCCCGCAGA   1001

F002               1106 --------------------------------------------------   1106

F003               1002 TCACAAAGCACGTGGCACAGATCTGACTCCCGGATGACACTTAGTACGAC   1051

F002               1106 --------------------------------------------------   1106

F003               1052 GAGATGACAGCCTGATCGGCAGTGAAGTGATCACCCTTGAGTTCAGCTTG   1101

F002               1106 ---------------------------------------   1106

F003               1102 GGTTCCGATTCGCAAAGAATTCCTAGCTTACCAAATTGC   1140

F003.fasta-R004rev.fasta
F003                  1 -----------------------GGCCAGGCTACACGA-TCTGCTGAA-T     25
                                               ||.||..|..||||| ||||||||| .
R004rev               1 GATGGTCTGATTCAACGCCTCCTGGGCACACATCACGATTCTGCTGAAGC     50

F003                 26 TATCAAGGACAAGGACTTCCTGGACAATGAGGAAAACGAGGACATTCTGG     75
                        .||| ||||| |.||||.|.||..|||||| |||||||| |||| ||||.
R004rev              51 AATC-AGGAC-ATGACTGCTTGACCAATGA-GAAAACGA-GACA-TCTGA     95

F003                 76 AAGATATCGTGCTGACCCTGACACTGTTTGAGGACAGAGAGATGATCGAG    125
                        ||||||||||||||| |||||||||||||||||||||||||||||||||
R004rev              96 AAGATATCGTGCTGA-CCTGACACTGTTTGAGGACAGAGAGATGATCGA-    143

F003                126 GAACGGCTGAAAACCTATG-CCCACCTGTTCGACGACAAAGTGATGAAGC    174
                        ||||||||||||||||||| ||||||||||||||||||||||||||||||
R004rev             144 GAACGGCTGAAAACCTATGCCCCACCTGTTCGACGACAAAGTGATGAAGC    193

F003                175 AGCTGAAGCGGCGGAGATACACCGGCTGGGGCAGGCTGAGCCGGAAGCTG    224
                        ||||||||||||||||||||||||||||||||||||||||||||||||||
R004rev             194 AGCTGAAGCGGCGGAGATACACCGGCTGGGGCAGGCTGAGCCGGAAGCTG    243
```

```
F003                 225 ATCAACGGCATCCGGGACAAGCAGTCCGGCAAGACAATCCTGGATTTCCT    274
                         ||||||||||||||||||||||||||||||||||||||||||||||| ||
R004rev              244 ATCAACGGCATCCGGGACAAGCAGTCCGGCAAGACAATCCTGGATTT-CT    292

F003                 275 GAAGTCCGACGGCTTCGCCAACAGAAACTTCATGCAGCTGATCCACGACG    324
                         ||||||||||||||||||||||||||||||||||||||||||||||||||
R004rev              293 GAAGTCCGACGGCTTCGCCAACAGAAACTTCATGCAGCTGATCCACGACG    342

F003                 325 ACAGCCTGACCTTTAAAGAGGACATCCAGAAAG-CCCAGGTGTCCGGCCA    373
                         |||||.||||||||||||||||||||||||||| ||||||||||||||||
R004rev              343 ACAGCGTGACCTTTAAAGAGGACATCCAGAAAGCCCCAGGTGTCCGGCCA    392

F003                 374 GGGCGATAGCCTGCACGAGCACATTGCCAATCTGGCCGGCAGCCCCGCCA    423
                         ||||||||||||||||||||||||||||||||||||||||||||||||||
R004rev              393 GGGCGATAGCCTGCACGAGCACATTGCCAATCTGGCCGGCAGCCCCGCCA    442

F003                 424 TTAAGAAGGGCATCCTGCAGACAGTGAAGGTGGTGGACGAGCTCGTGAAA    473
                         ||||||||||||||||||||||||||||||||||||||||||||||||||
R004rev              443 TTAAGAAGGGCATCCTGCAGACAGTGAAGGTGGTGGACGAGCTCGTGAAA    492

F003                 474 GTGATGGGCCGGCACAAGCCCGAGAACATCGTGATCGAAATGGCCAGAGA    523
                         ||||||||||||||||||||||||||||||||||||||||||||||||||
R004rev              493 GTGATGGGCCGGCACAAGCCCGAGAACATCGTGATCGAAATGGCCAGAGA    542

F003                 524 GAACCAGACCACCCAGAAGGGACAGAAGAACAGCCGCGAGAGAATGAAGC    573
                         ||||||||||||||||||||||||||||||||||||||||||||||||||
R004rev              543 GAACCAGACCACCCAGAAGGGACAGAAGAACAGCCGCGAGAGAATGAAGC    592

F003                 574 GGATCGAAGAGGGCATCAAAGAGCTGGGCAGCCAGATCCTGAAAGAACAC    623
                         ||||||||||||||||||||||||||||||||||||||||||||||||||
R004rev              593 GGATCGAAGAGGGCATCAAAGAGCTGGGCAGCCAGATCCTGAAAGAACAC    642

F003                 624 CCCGTGGAAAACACCCAGCTGCAGAACGAGAAGCTGTACCTGTACTACCT    673
                         ||||||||||||||||||||||||||||||||||||||||||||||||||
R004rev              643 CCCGTGGAAAACACCCAGCTGCAGAACGAGAAGCTGTACCTGTACTACCT    692

F003                 674 GCAGAATGGGCGGGATATGTACGTGGACCAGGAACTGGACATCAACCGGC    723
                         ||||||||||||||||||||||||||||||||||||||||||||||||||
R004rev              693 GCAGAATGGGCGGGATATGTACGTGGACCAGGAACTGGACATCAACCGGC    742

F003                 724 TGTCCGACTACGATGTGGACCATATCGTGCCTCAGAGCTTTCTGAAGGAC    773
                         ||||||||||||||||||||||||||||||||||||||||||||||||||
R004rev              743 TGTCCGACTACGATGTGGACCATATCGTGCCTCAGAGCTTTCTGAAGGAC    792

F003                 774 GACTCCATCGACAACAAAGTGCTGACCAGAAGCGACAAGAACCGGGGCAA    823
                         |||||||||||||||||.||||||||||||||||||||||||||||||||
R004rev              793 GACTCCATCGACAACAAGGTGCTGACCAGAAGCGACAAGAACCGGGGCAA    842

F003                 824 GAGCGACAACGTGCCCTCCGAAGAGGTCGTGAAGAAGATGAAGAACTACT    873
                         ||||||||||||||||||||||||||||||||||||||||||||||||||
R004rev              843 GAGCGACAACGTGCCCTCCGAAGAGGTCGTGAAGAAGATGAAGAACTACT    892

F003                 874 GGCGGCAGCTGCTGAACGCCCAAGCTGATTACCCAGAGAAAGTTCGAC-A    922
                         |||||||||||||||||| ||||||||||||||||||||||||||||| |
R004rev              893 GGCGGCAGCTGCTGAACG-CCAAGCTGATTACCCAGAGAAAGTTCGACAA    941

F003                 923 TCTGACCAACGCCGAGAGAGGCGGCCTGAGCGAACTGGATAATGC--GCT    970
                         |||||||||.||||||||||||||||||||||||||||||||.||  |||
R004rev              942 TCTGACCAAGGCCGAGAGAGGCGGCCTGAGCGAACTGGATAAGGCCGGCT    991

F003                 971 TCATCAGGAGACAGCTGATG--AACCCCGCAGATCACAAAGCACGTGGCA   1018
                         ||||||.||||||||||.||  |||||.||||||||||||||||||||||
R004rev              992 TCATCAAGAGACAGCTGGTGGAAACCCGGCAGATCACAAAGCACGTGGCA   1041

F003                1019 CAGATC--TGACTCCCGGATG-ACACTTAGTACGACGAG-ATGACAGCCT   1064
                         ||||||  .|||||||||||| |||||.||||||||||| ||||||..||
R004rev             1042 CAGATCCTGGACTCCCGGATGAACACTAAGTACGACGAGAATGACAAGCT   1091

F003                1065 GATC--GGCAGTG-AAGTGATCACCCTTGAGTTC-AGCTTGGGT-TCCGA   1109
                         ||||  ||.|||| |||||||||||||..|||.| ||||  ||| |||||
R004rev             1092 GATCCGGGAAGTGAAAGTGATCACCCTGAAGTCCAAGCT--GGTGTCCGA   1139

F003                1110 TTCGCAAAGAATTCCTAGCTTACCAAATTGC   1140
                         |.||  |||||  ||.|
```

```
R004rev           1140 TCCG--AAGAA--CCGA--------------   1152

R004rev.fasta-R005rev.fasta
R004rev                1 GATGGTCTGATTCAACGCCTCCTGGGCACACATCACGATTCTGCTGAAGC     50

R005rev                0 --------------------------------------------------      0

R004rev               51 AATCAGGACATGACTGCTTGACCAATGAGAAAACGAGACATCTGAAAGAT    100

R005rev                0 --------------------------------------------------      0

R004rev              101 ATCGTGCTGACCTGACACTGTTTGAGGACAGAGAGATGATCGAGAACGGC    150

R005rev                0 --------------------------------------------------      0

R004rev              151 TGAAAACCTATGCCCCACCTGTTCGACGACAAAGTGATGAAGCAGCTGAA    200

R005rev                0 --------------------------------------------------      0

R004rev              201 GCGGCGGAGATACACCGGCTGGGGCAGGCTGAGCCGGAAGCTGATCAACG    250

R005rev                0 --------------------------------------------------      0

R004rev              251 GCATCCGGGACAAGCAGTCCGGCAAGACAATCCTGGATTTCTGAAGTCCG    300

R005rev                0 --------------------------------------------------      0

R004rev              301 ACGGCTTCGCCAACAGAAACTTCATGCAGCTGATCCACGACGACAGCGTG    350

R005rev                0 --------------------------------------------------      0

R004rev              351 ACCTTTAAAGAGGACATCCAGAAAGCCCCAGGTGTCCGGCCAGGGCGATA    400

R005rev                0 --------------------------------------------------      0

R004rev              401 GCCTGCACGAGCACATTGCCAATCTGGCCGGCAGCCCCGCCATTAAGAAG    450

R005rev                0 --------------------------------------------------      0

R004rev              451 GGCATCCTGCAGACAGTGAAGGTGGTGGACGAGCTCGTGAAAGTGATGGG    500

R005rev                0 --------------------------------------------------      0

R004rev              501 CCGGCACAAGCCCGAGAACATCGTGATCGAAATGGCCAGAGAGAACCAGA    550

R005rev                0 --------------------------------------------------      0

R004rev              551 CCACCCAGAAGGGACAGAAGAACAGCCGCGAGAGAATGAAGCGGATCGAA    600

R005rev                0 --------------------------------------------------      0

R004rev              601 GAGGGCATCAAAGAGCTGGGCAGCCAGATCCTGAAAGAACACCCCGTGGA    650

R005rev                0 --------------------------------------------------      0

R004rev              651 AAACACCCAGCTGCAGAACGAGAAGCTGTACCTGTACTACCTGCAGAATG    700

R005rev                0 --------------------------------------------------      0

R004rev              701 GGCGGGATATGTACGTGGACCAGGAACTGGACATCAACCGGCTGTCCGAC    750

R005rev                0 --------------------------------------------------      0

R004rev              751 TACGATGTGGACCATATCGTGCCTCAGAGCTTTCTGAAGGACGACTCCAT    800
                                                     ||||||||||||||||||||||
R005rev                1 ----------------------------GCTTTCTGAAGGACGACTCCAT     22

R004rev              801 CGACAACAAGGTGCTGACCAGAAGCGACAAGAACCGGGGCAAGAGCGACA    850
                         |||| ||||||||||||||||||||||||  .|||||||| |||||||| 
R005rev               23 CGAC-ACAAGGTGCTGACCAGAAGCGACA--GACCGGGGC-AGAGCGAC-     67

R004rev              851 ACGTGCCCTCCGAAGAGGTCGTGAAGAAGATGAAGAACTACTGGCGGCAG    900
                         |||||||||||||||| |||||| ||||||||||||||||||||||||||
R005rev               68 ACGTGCCCTCCGAAGA-GTCGTG-AGAAGATGAAGAACTACTGGCGGCAG    115
```

```
R004rev        901  CTGCTGAACGCCAAGCTGATTACCCAGAGAAAGTTCGACAATCTGACCAA   950
                    ||||||||||||||||||||||||||||||||||||||||||||||||||
R005rev        116  CTGCTGAACGCCAAGCTGATTACCCAGAGAAAGTTCGACAATCTGACCAA   165

R004rev        951  GG-CCGAGAGAGGCGGCCTGAGCGAACTGGATAAGG-CCGGCTTCATCAA   998
                    || ||||||||||||| |||||||||||||||||| ||||||||||||||
R005rev        166  GGCCCGAGAGAGGCGG-CTGAGCGAACTGGATAAGGCCCGGCTTCATCAA   214

R004rev        999  GAGACAGCTGGTGGAAACCCGGCAGATCACAAAGCACGTGGCACAGATCC  1048
                    ||||||||||||||||||||||||||||||||||||||||||||||||||
R005rev        215  GAGACAGCTGGTGGAAACCCGGCAGATCACAAAGCACGTGGCACAGATCC   264

R004rev       1049  TGGACTCCCGGATGAACACTAAGTACGACGAGAATGACAAGCTGATCCGG  1098
                    ||||||||||||||||||||||||||||||||||||||||||||||||||
R005rev        265  TGGACTCCCGGATGAACACTAAGTACGACGAGAATGACAAGCTGATCCGG   314

R004rev       1099  GAAGTGAAAGTGATCACCCTGAAGTCCAAGCTGGTGTCCGAT---CCGAA  1145
                    ||||||||||||||||||||||||||||||||||||||||||   |.|||
R005rev        315  GAAGTGAAAGTGATCACCCTGAAGTCCAAGCTGGTGTCCGATTTCCGGAA   364

R004rev       1146  GAACCGA-------------------------------------------  1152
                    |    ||
R005rev        365  G----GATTTCCAGTTTTACAAAGTGCGCGAGATCAACAACTACCACCAC   410

R004rev       1152  --------------------------------------------------  1152

R005rev        411  GCCCACGACGCCTACCTGAACGCCGTCGTGGGAACCGCCCTGATCAAAAA   460

R004rev       1152  --------------------------------------------------  1152

R005rev        461  GTACCCTAAGCTGGAAAGCGAGTTCGTGTACGGCGACTACAAGGTGTACG   510

R004rev       1152  --------------------------------------------------  1152

R005rev        511  ACGTGCGGAAGATGATCGCCAAGAGCGAGCAGGAAATCGGCAAGGCTACC   560

R004rev       1152  --------------------------------------------------  1152

R005rev        561  GCCAAGTACTTCTTCTACAGCAACATCATGAACTTTTTCAAGACCGAGAT   610

R004rev       1152  --------------------------------------------------  1152

R005rev        611  TACCCTGGCCAACGGCGAGATCCGGAAGCGGCCTCTGATCGAGACAAACG   660

R004rev       1152  --------------------------------------------------  1152

R005rev        661  GCGAAACCGGGGAGATCGTGTGGGATAAGGGCCGGGATTTTGCCACCGTG   710

R004rev       1152  --------------------------------------------------  1152

R005rev        711  CGGAAAGTGCTGAGCATGCCCCAAGTGAATATCGTGAAAAAGACCGAGGT   760

R004rev       1152  --------------------------------------------------  1152

R005rev        761  GCAGACAGGCGGCTTCAGCAAAGAGTCTATCCTGCCCAAGAGGAACAGCG   810

R004rev       1152  --------------------------------------------------  1152

R005rev        811  ATAAGCTGATCGCCAGAAAGAAGGACTGGGACCCTAAGAAGTACGGCGGC   860

R004rev       1152  --------------------------------------------------  1152

R005rev        861  TTCGACAGCCCCACCGTGGCCTATTCTGTGCTGGTGGTGGCCAAAGTGGA   910

R004rev       1152  --------------------------------------------------  1152

R005rev        911  AAAGGGCAAGTCCAAGAAACTGAAGAGTGTGAAAGAGCTGCTGGGGATCA   960

R004rev       1152  --------------------------------------------------  1152

R005rev        961  CCATCATGGAAAGAAGCAGCTTCGAGAAGAATCCCATCGACTTCTGAAGC  1010

R004rev       1152  --------------   1152

R005rev       1011  CAAGCACAGTGAGG   1024
```

```
R005rev.fasta-R006rev.fasta
R005rev                  1 GCTTTCTGAAGGACGACTCCATCGACACAAGGTGCTGACCAGAAGCGACA     50

R006rev                  0 --------------------------------------------------      0

R005rev                 51 GACCGGGGCAGAGCGACACGTGCCCTCCGAAGAGTCGTGAGAAGATGAAG    100

R006rev                  0 --------------------------------------------------      0

R005rev                101 AACTACTGGCGGCAGCTGCTGAACGCCAAGCTGATTACCCAGAGAAAGTT    150

R006rev                  0 --------------------------------------------------      0

R005rev                151 CGACAATCTGACCAAGGCCCGAGAGAGGCGGCTGAGCGAACTGGATAAGG    200

R006rev                  0 --------------------------------------------------      0

R005rev                201 CCCGGCTTCATCAAGAGACAGCTGGTGGAAACCCGGCAGATCACAAAGCA    250

R006rev                  0 --------------------------------------------------      0

R005rev                251 CGTGGCACAGATCCTGGACTCCCGGATGAACACTAAGTACGACGAGAATG    300

R006rev                  0 --------------------------------------------------      0

R005rev                301 ACAAGCTGATCCGGGAAGTGAAAGTGATCACCCTGAAGTCCAAGCTGGTG    350

R006rev                  0 --------------------------------------------------      0

R005rev                351 TCCGATTTCCGGAAGGATTTCCAGTTTTACAAAGTGCGCGAGATCAACAA    400

R006rev                  0 --------------------------------------------------      0

R005rev                401 CTACCACCACGCCCACGACGCCTACCTGAACGCCGTCGTGGGAACCGCCC    450

R006rev                  0 --------------------------------------------------      0

R005rev                451 TGATCAAAAAGTACCCTAAGCTGGAAAGCGA--GTTCGTG-TACGGC-GA    496
                                              ||| |||.||||  .||||.| ||||.| ||
R006rev                  1 -------------------GCT-GAAGGCGAAGTTTCGAGTTACGACGGA     30

R005rev                497 CTACAAGGTGTACGACGTGCGGAAGATGATCGCCAAGAGCG-AGCAGGAA    545
                           |||||| |.|||||||||.| |||||||||||.|||||||| |||..|||
R006rev                 31 CTACAA-GAGTACGACGTAC-GAAGATGATCGGCAAGAGCGAAGCCAGAA     78

R005rev                546 ATCGGCAAGGCTACCGCCAAGTACTTCTTCTACAG-CAACATCATGAACT    594
                           |||.||.|.|||| ||||||||||||||||||||| ||.|||||||||||
R006rev                 79 ATCCGCCAAGCTA-CGCCAAGTACTTCTTCTACAGCCACCATCATGAACT    127

R005rev                595 TTTTCAAGACCGAGATTACCCTGGCCAACG-GCGAGATCC-GGAAGCGGC    642
                           ||||||||| |||||||||||| ||||||| ||||||||| |.|||||||
R006rev                128 TTTTCAAGA-CGAGATTACCCT-GCCAACGAGCGAGATCCAGAAAGCGGC    175

R005rev                643 CTCTGATCGAGACAAACGGCGAAACCGGGGAGATCGTGTGGGATAAGGGC    692
                           ||||||||||||| |.|||||||||||.|||||||||||||||||||.||
R006rev                176 CTCTGATCGAGAC-ATCGGCGAAACCCGGGAGATCGTGTGGGATAAGAGC    224

R005rev                693 CGGGATTTTGCCACCGTGCGGAAAGTGCTGAGCATGCCCCAAGTGAATAT    742
                           |||||||||| |||||||||||||||||||||||||||||||||||||||
R006rev                225 CGGGATTTTG-CACCGTGCGGAAAGTGCTGAGCATGCCCCAAGTGAATAT    273

R005rev                743 CGTGAAAAAGACCGAGGTGCAGACAGGCGGCTTCAGCAAAGAGTCTATCC    792
                           ||||||||||||||||||||||||||||||||||||||||||||||||||
R006rev                274 CGTGAAAAAGACCGAGGTGCAGACAGGCGGCTTCAGCAAAGAGTCTATCC    323

R005rev                793 TGCCCAAGAGGAACAGCGATAAGCTGATCGCCAGAAAGAAGGACTGGGAC    842
                           ||||||||||||||||||||||||||||||||||||||||||||||||||
R006rev                324 TGCCCAAGAGGAACAGCGATAAGCTGATCGCCAGAAAGAAGGACTGGGAC    373

R005rev                843 CCTAAGAAGTACGGCGGCTTCGACAGCCCCACCGTGGCCTATTCTGTGCT    892
                           ||||||||||||||||||||||||||||||||||||||||||||||||||
R006rev                374 CCTAAGAAGTACGGCGGCTTCGACAGCCCCACCGTGGCCTATTCTGTGCT    423

R005rev                893 GGTGGTGGCCAAAGTGGAAAAGGGCAAGTCCAAGAAACTGAAGAGTGTGA    942
```

```
                                 ||||||||||||||||||||||||||||||||||||||||||||||||||
R006rev                      424 GGTGGTGGCCAAAGTGGAAAAGGGCAAGTCCAAGAAACTGAAGAGTGTGA    473

R005rev                      943 AAGAGCTGCTGGGGATCACCATCATGGAAAGAAGCAGCTTCGAGAAGAAT    992
                                 ||||||||||||||||||||||||||||||||||||||||||||||||||
R006rev                      474 AAGAGCTGCTGGGGATCACCATCATGGAAAGAAGCAGCTTCGAGAAGAAT    523

R005rev                      993 CCCATCGAC-TTCT-GAAGCCAAGCAC--------AGTG----AGG----   1024
                                 ||||||||| |||| |||||||||..|        ||||    |||
R006rev                      524 CCCATCGACTTTCTGGAAGCCAAGGGCTACAAAGAAGTGAAAAAGGACCT    573

R005rev                     1024 --------------------------------------------------   1024

R006rev                      574 GATCATCAAGCTGCCTAAGTACTCCCTGTTCGAGCTGGAAAACGGCCGGA    623

R005rev                     1024 --------------------------------------------------   1024

R006rev                      624 AGAGAATGCTGGCCTCTGCCGGCGAACTGCAGAAGGGAAACGAACTGGCC    673

R005rev                     1024 --------------------------------------------------   1024

R006rev                      674 CTGCCCTCCAAATATGTGAACTTCCTGTACCTGGCCAGCCACTATGAGAA    723

R005rev                     1024 --------------------------------------------------   1024

R006rev                      724 GCTGAAGGGCTCCCCCGAGGATAATGAGCAGAAACAGCTGTTTGTGGAAC    773

R005rev                     1024 --------------------------------------------------   1024

R006rev                      774 AGCACAAGCACTACCTGGACGAGATCATCGAGCAGATCAGCGAGTTCTCC    823

R005rev                     1024 --------------------------------------------------   1024

R006rev                      824 AAGAGAGTGATCCTGGCCGACGCTAATCTGGACAAAGTGCTGTCCGCCTA    873

R005rev                     1024 --------------------------------------------------   1024

R006rev                      874 CAACAAGCACCGGGATAAGCCCATCAGAGAGCAGGCCGAGAATATCATCC    923

R005rev                     1024 --------------------------------------------------   1024

R006rev                      924 ACCTGTTTACCCTGACCAATCTGGGAGCCCCTGCCGCCTTCAAGTACTTT    973

R005rev                     1024 --------------------------------------------------   1024

R006rev                      974 GACACCACCATCGACCGGAAGAGGTACACCAGCACCAAAGAGGTGCTGGA   1023

R005rev                     1024 --------------------------------------------------   1024

R006rev                     1024 CGCCACCCTGATCCACCAGAGCATCACCGGCCTGTACGAGACACGGATCG   1073

R005rev                     1024 --------------------------------------------------   1024

R006rev                     1074 ACCTGTCTCAGCTGGGAGGCGACCCAAAGAAGAAGCGGAAGGTCTGAAAG   1123

R005rev                     1024 --------------------------------------------------   1024

R006rev                     1124 CTTGCGGCCGCACTCGAGCACCACCACCACCACCACTGAGATCCGGCTGC   1173

R005rev                     1024 --------------------------    1024

R006rev                     1174 TAACAAAGCCCGAAAGAGCGATTTCC    1199
```

4. The numbers with red highlight are F000 (1-700), F001 (23-700), F002 (44-697), F003 (56-174), R004rev (194-900), R005rev (116-742), R006rev (274-1199), which are shown in red highlight in the corresponding Sanger sequencing files as below.

```
>F000
GCTGACTCTTCCCTCTAGAATAATTTTGTTTAACTTTAAGAAGGAGATATACCATGGGCAGCAGCCATCATCATCATCATCACAGCAGCGGCCTGG
AAGTTCTGTTCCAGGGGCCCCATATGGCTAGCATGACTGGTGGACAGCAAATGGGTCGCGGATCCCCAAAGAAGAAGCGGAAGGTCGGTATCCACG
GAGTCCCAGCAGCCGACAAGAAGTACAGCATCGGCCTGGACATCGGCACCAACTCTGTGGGCTGGGCCGTGATCACCGACGAGTACAAGGTGCCCA
GCAAGAAATTCAAGGTGCTGGGCAACACCGACCGGCACAGCATCAAGAAGAACCTGATCGGAGCCCTGCTGTTCGACAGCGGCGAAACAGCCGAGG
CCACCCGGCTGAAGAGAACCGCCAGAAGAAGATACACCAGACGGAAGAACCGGATCTGCTATCTGCAAGAGATCTTCAGCAACGAGATGGCCAAGG
TGGACGACAGCTTCTTCCACAGACTGGAAGAGTCCTTCCTGGTGGAAGAGGATAAGAAGCACGAGCGGCACCCCATCTTCGGCAACATCGTGGACG
```

AGGTGGCCTACCACGAGAAGTACCCCACCATCTACCACCTGAGAAAGAAACTGGTGGACAGCACCGACAAGGCCGACCTGCGGCTGATCTATCTGG
CCCTGGCCCACATGATCAAGTTCCGGGGCCACTTCCTGATCGAGGGCGACCTGAACCCCGACAACAGCGACGTGGACAAGCTGTTCATCCAGCTGG
TGCAGACCTACAACCAGCTGTTCGAGGAAAACCCCATCAACGCCAGCGGCGTGGACGCCAAGGCCATCCTGTCTGCCAGACTGAGCAAGAGCAGAC
GGCTGGAAAATCTGATCGCCCAGCTGCCCGGCGAGAAGAAGAATGGCCCTGTTCGGAAACCTGATTGCCCTGAGCCTGGGCCTGACCCCCAACTTC
AAGAGCAACTTCGACCTGGCCGAGGATGCCAAACTGCAGCTGAGCAAGGACACCTACGACGACGACCTGACACCTGCTGGCCCAGATCGGCGACCA
GTACGCCGACCTGTTTTCTATGCAGACTGTCGACGCATCTGCTGACGACATCTGAGAGTGAACACGAGATCACAGCTCCCTTGACCTTATGATCAG
AGAATATCGATCGAGCACCTATCGAGACTGAACTCCTGACTGGA

> F001
CTGTATTTCGATCAGTTCGGGGCCACTTCCTGATCGAGGGCGACCTGAACCCCGACAACAGCGACGTGGACAAGCTGTTCATCCAGCTGGTGCAGA
CCTACAACCAGCTGTTCGAGGAAAACCCCATCAACGCCAGCGGCGTGGACGCCAAGGCCATCCTGTCTGCCAGACTGAGCAAGAGCAGACGGCTGG
AAAATCTGATCGCCCAGCTGCCCGGCGAGAAGAAGAATGGCCTGTTCGGAAACCTGATTGCCCTGAGCCTGGGCCTGACCCCCAACTTCAAGAGCA
ACTTCGACCTGGCCGAGGATGCCAAACTGCAGCTGAGCAAGGACACCTACGACGACGACCTGGACAACCTGCTGGCCCAGATCGGCGACCAGTACG
CCGACCTGTTTCTGGCCGCCAAGAACCTGTCCGACGCCATCCTGCTGAGCGACATCCTGAGAGTGAACACCGAGATCACCAAGGCCCCCCTGAGCG
CCTCTATGATCAAGAGATACGACGAGCACCACCAGGACCTGACCCTGCTGAAAGCTCTCGTGCGGCAGCAGCTGCCTGAGAAGTACAAAGAGATTT
TCTTCGACCAGAGCAAGAACGGCTACGCCGGCTACATTGACGGCGGAGCCAGCCAGGAAGAGTTCTACAAGTTCATCAAGCCCATCCTGGAAAAGA
TGGACGGCACCGAGGAACTGCTCGTGAAGCTGAACAGAGAGGACCTGCTGCGGAAGCAGCGGACCTTCGACAACGGCAGCATCCCCCACCAGATCC
ACCTGGGAGAGCTGCACGCCATTCTGCGGCGGCAGGAAGATTTTTACCCATTCCTGAAGGACAACCGGGAAAAGATCGAGAAGATCCTGACCTTCC
GCATCCCCTACTACGTGGGCCCTCTGGGCCAGGGGAAACAGCAGATTCGCCTGGATGACCAGAAGAGCGAGGAAACCATCACCCCCTGCACTTCGA
GGAAGTGTGGACAGGCGCTCGCCAGAGCTCATCGAGCGATGACACTCGATAGACTGCCAACGAGAGGTGCTGCCAGCACAGCTGCTGTA

>F002
GGGATCATCTGGAAGTGGACGGCACCGAGGAACTGCTCGTGAAGCTGAACAGAGAGGACCTGCTGCGGAAGCAGCGGACCTTCGACAACGGCAGCA
TCCCCCACCAGATCCACCTGGGAGAGCTGCACGCCATTCTGCGGCGGCAGGAAGATTTTTACCCATTCCTGAAGGACAACCGGGAAAAGATCGAGA
AGATCCTGACCTTCCGCATCCCCTACTACGTGGGCCCTCTGGCCAGGGGAAACAGCAGATTCGCCTGGATGACCAGAAAGAGCGAGGAAACCATCA
CCCCCTGGAACTTCGAGGAAGTGGTGGACAAGGGCGCTTCCGCCCAGAGCTTCATCGAGCGGATGACCAACTTCGATAAGAACCTGCCCAACGAGA
AGGTGCTGCCCAAGCACAGCCTGCTGTACGAGTACTTCACCGTGTATAACGAGCTGACCAAAGTGAAATACGTGACCGAGGGAATGAGAAAGCCCG
CCTTCCTGAGCGGCGAGCAGAAAAAGGCCATCGTGGACCTGCTGTTCAAGACCAACCGGAAAGTGACCGTGAAGCAGCTGAAAGAGGACTACTTCA
AGAAAATCGAGTGCTTCGACTCCGTGGAAATCTCCGGCGTGGAAGATCGGTTCAACGCCTCCCTGGGCACATACCACGATCTGCTGAAAATTATCA
AGGACAAGGACTTCCTGGACAATGAGGAAAACGAGGACATTCTGGAAGATATCGTGCTGACCCTGACACTGTTTGAGGACAGAGAGATGATCGAGG
AACGGCTGAAAACCTATGCCCACCTGTTCGACGACAAAGTGATGAAGCAGCTGAAGCGGCGGAGATACACCGGCTGGGGCAGGCTGAGCCGGAAGC
TGATCAACGGCATCCGGGACAAGCAGTCCGGGCAGACATCCTGGATTTCCTGAAGTCCGACGGCTTCGCAACAGAAACTCATGCAGCTGATCACGA
CGACAGCTGACTTAAGAGACATCAGATGCCAGTGTCGCAGGCGATAGCTGCACGAGCAATGGCATCTGTGCAGGCCGCATAGAGCATCTGCGACTG
AGTGTGACAGCTCTGAATGATGACCGCCAAGCCGAAAACTCTGGTATTCG

>F003
GGCCAGGCTACACGATCTGCTGAATTATCAAGGACAAGGACTTCCTGGACAATGAGGAAAACGAGGACATTCTGGAAGATATCGTGCTGACCCTGA
CACTGTTTGAGGACAGAGAGATGATCGAGGAACGGCTGAAAACCTATGCCCACCTGTTCGACGACAAAGTGATGAAGCAGCTGAAGCGGCGGAGAT
ACACCGGCTGGGGCAGGCTGAGCCGGAAGCTGATCAACGGCATCCGGGACAAGCAGTCCGGCAAGACAATCCTGGATTTCCTGAAGTCCGACGGCT
TCGCCAACAGAAACTTCATGCAGCTGATCCACGACGACAGCCTGACCTTTAAAGAGGACATCCAGAAAGCCCAGGTGTCCGGCCAGGGCGATAGCC
TGCACGAGCACATTGCCAATCTGGCCGGCAGCCCCGCCATTAAGAAGGGCATCCTGCAGACAGTGAAGGTGGTGGACGAGCTCGTGAAAGTGATGG
GCCGGCACAAGCCCGAGAACATCGTGATCGAAATGGCCAGAGAGAACCAGACCACCCAGAAGGGACAGAAGAACAGCCGCGAGAGAATGAAGCGGA
TCGAAGAGGGCATCAAAGAGCTGGGCAGCCAGATCCTGAAAGAACACCCCGTGGAAAACACCCAGCTGCAGAACGAGAAGCTGTACCTGTACTACC
TGCAGAATGGGCGGGATATGTACGTGGACCAGGAACTGGACATCAACCGGCTGTCCGACTACGATGTGGACCATATCGTGCCTCAGAGCTTTCTGA
AGGACGACTCCATCGACAACAAAGTGCTGACCAGAAGCGACAAGAACCGGGGCAAGAGCGACAACGTGCCCTCCGAAGAGGTCGTGAAGAAGATGA
AGAACTACTGGCGGCAGCTGCTGAACGCCCAAGCTGATTACCCAGAGAAAGTTCGACATCTGACCAACGCCGAGAGAGGCGGCCTGAGCGAACTGG
ATAATGCGCTTCATCAGGAGACAGCTGATGAACCCCGCAGATCACAAAGCACGTGGCACAGATCTGACTCCCGGATGACACTTAGTACGACGAGAT
GACAGCCTGATCGGCAGTGAAGTGATCACCCTTGAGTTCAGCTTGGGTTCCGATTCGCAAAGAATTCCTAGCTTACCAAATTGC

>R004rev
GATGGTCTGATTCAACGCCTCCTGGGCACACATCACGATTCTGCTGAAGCAATCAGGACATGACTGCTTGACCAATGAGAAAACGAGACATCTGAA
AGATATCGTGCTGACCTGACACTGTTTGAGGACAGAGAGATGATCGAGAACGGCTGAAAACCTATGCCCCACCTGTTCGACGACAAAGTGATGAAG
CAGCTGAAGCGGCGGAGATACACCGGCTGGGGCAGGCTGAGCCGGAAGCTGATCAACGGCATCCGGGACAAGCAGTCCGGCAAGACAATCCTGGAT
TTCTGAAGTCCGACGGCTTCGCCAACAGAAACTTCATGCAGCTGATCCACGACGACAGCGTGACCTTTAAAGAGGACATCCAGAAAGCCCCAGGTG
TCCGGCCAGGGCGATAGCCTGCACGAGCACATTGCCAATCTGGCCGGCAGCCCCGCCATTAAGAAGGGCATCCTGCAGACAGTGAAGGTGGTGGAC
GAGCTCGTGAAAGTGATGGGCCGGCACAAGCCCGAGAACATCGTGATCGAAATGGCCAGAGAGAACCAGACCACCCAGAAGGGACAGAAGAACAGC
CGCGAGAGAATGAAGCGGATCGAAGAGGGCATCAAAGAGCTGGGCAGCCAGATCCTGAAAGAACACCCCGTGGAAAACACCCAGCTGCAGAACGAG
AAGCTGTACCTGTACTACCTGCAGAATGGGCGGGATATGTACGTGGACCAGGAACTGGACATCAACCGGCTGTCCGACTACGATGTGGACCATATC
GTGCCTCAGAGCTTTCTGAAGGACGACTCCATCGACAACAAGGTGCTGACCAGAAGCGACAAGAACCGGGGCAAGAGCGACAACGTGCCCTCCGAA
GAGGTCGTGAAGAAGATGAAGAACTACTGGCGGCAGCTGCTGAACGCCAAGCTGATTACCCAGAGAAAGTTCGACAATCTGACCAAGGCCGAGAGA
GGCGGCCTGAGCGAACTGGATAAGGCCGGCTTCATCAAGAGACAGCTGGTGGAAACCCGGCAGATCACAAAGCACGTGGCACAGATCCTGGACTCC
CGGATGAACACTAAGTACGACGAGAATGACAAGCTGATCCGGGAAGTGAAAGTGATCACCCTGAAGTCCAAGCTGGTGTCCGATCCGAAGAACCGA

> R005rev
GCTTTCTGAAGGACGACTCCATCGACACAAGGTGCTGACCAGAAGCGACAGACCGGGGCAGAGCGACACGTGCCCTCCGAAGAGTCGTGAGAAGAT
GAAGAACTACTGGCGGCAGCTGCTGAACGCCAAGCTGATTACCCAGAGAAAGTTCGACAATCTGACCAAGGCCCGAGAGAGGCGGCTGAGCGAACT
GGATAAGGCCCGGCTTCATCAAGAGACAGCTGGTGGAAACCCGGCAGATCACAAAGCACGTGGCACAGATCCTGGACTCCCGGATGAACACTAAGT
ACGACGAGAATGACAAGCTGATCCGGGAAGTGAAAGTGATCACCCTGAAGTCCAAGCTGGTGTCCGATTTCCGGAAGGATTTCCAGTTTTACAAAG
TGCGCGAGATCAACAACTACCACCACGCCCACGACGCCTACCTGAACGCCGTCGTGGGAACCGCCCTGATCAAAAAGTACCCTAAGCTGGAAAGCG
AGTTCGTGTACGGCGACTACAAGGTGTACGACGTGCGGAAGATGATCGCCAAGAGCGAGCAGGAAATCGGCAAGGCTACCGCCAAGTACTTCTTCT
ACAGCAACATCATGAACTTTTTCAAGACCGAGATTACCCTGGCCAACGGCGAGATCCGGAAGCGGCCTCTGATCGAGACAAACGGCGAAACCGGGG
AGATCGTGTGGGATAAGGGCCGGGATTTTGCCACCGTGCGGAAAGTGCTGAGCATGCCCCAAGTGAATATCGTGAAAAAGACCGAGGTGCAGACAG
GCGGCTTCAGCAAAGAGTCTATCCTGCCCAAGAGGAACAGCGATAAGCTGATCGCCAGAAAGAAGGACTGGGACCCTAAGAAGTACGGCGGCTTCG
ACAGCCCCACCGTGGCCTATTCTGTGCTGGTGGTGGCCAAAGTGGAAAAGGGCAAGTCCAAGAAACTGAAGAGTGTGAAAGAGCTGCTGGGGATCA
CCATCATGGAAAGAAGCAGCTTCGAGAAGAATCCCATCGACTTCTGAAGCCAAGCACAGTGAGG

> R006rev
GCTGAAGGCGAAGTTTCGAGTTACGACGGACTACAAGAGTACGACGTACGAAGATGATCGGCAAGAGCGAAGCCAGAAATCCGCCAAGCTACGCCA
AGTACTTCTTCTACAGCCACCATCATGAACTTTTTCAAGACGAGATTACCCTGCCAACGAGCGAGATCCAGAAAGCGGCCTCTGATCGAGACATCG
GCGAAACCCGGGAGATCGTGTGGGATAAGAGCCGGGATTTTGCACCGTGCGGAAAGTGCTGAGCATGCCCCAAGTGAATATCGTGAAAAAGACCGA
GGTGCAGACAGGCGGCTTCAGCAAAGAGTCTATCCTGCCCAAGAGGAACAGCGATAAGCTGATCGCCAGAAAGAAGGACTGGGACCCTAAGAAGTA
CGGCGGCTTCGACAGCCCCACCGTGGCCTATTCTGTGCTGGTGGTGGCCAAAGTGGAAAAGGGCAAGTCCAAGAAACTGAAGAGTGTGAAAGAGCT
GCTGGGGATCACCATCATGGAAAGAAGCAGCTTCGAGAAGAATCCCATCGACTTTCTGGAAGCCAAGGGCTACAAAGAAGTGAAAAAGGACCTGAT
CATCAAGCTGCCTAAGTACTCCCTGTTCGAGCTGGAAAACGGCCGGAAGAGAATGCTGGCCTCTGCCGGCGAACTGCAGAAGGGAAACGAACTGGC
CCTGCCCTCCAAATATGTGAACTTCCTGTACCTGGCCAGCCACTATGAGAAGCTGAAGGGCTCCCCCGAGGATAATGAGCAGAAACAGCTGTTTGT
GGAACAGCACAAGCACTACCTGGACGAGATCATCGAGCAGATCAGCGAGTTCTCCAAGAGAGTGATCCTGGCCGACGCTAATCTGGACAAAGTGCT
GTCCGCCTACAACAAGCACCGGGATAAGCCCATCAGAGAGCAGGCCGAGAATATCATCCACCTGTTTACCCTGACCAATCTGGGAGCCCCTGCCGC
CTTCAAGTACTTTGACACCACCATCGACCGGAAGAGGTACACCAGCACCAAAGAGGTGCTGGACGCCACCCTGATCCACCAGAGCATCACCGGCCT
GTACGAGACACGGATCGACCTGTCTCAGCTGGGAGGCGACCCAAAGAAGAAGCGGAAGGTCTGAAAGCTTGCGGCCGCACTCGAGCACCACCACCA
CCACCACTGAGATCCGGCTGCTAACAAAGCCCGAAAGAGCGATTTCC

5. The nucleotides in red highlight in step 4 are joined via copy and paste to obtain the merged Sanger sequence, as shown below.

>gene
GCTGACTCTTCCCTCTAGAATAATTTTGTTTAACTTTAAGAAGGAGATATACCATGGGCAGCAGCCATCATCATCATCATCACAGCAGCGGCCTGG
AAGTTCTGTTCCAGGGGCCCCATATGGCTAGCATGACTGGTGGACAGCAAATGGGTCGCGGATCCCCAAAGAAGAAGCGGAAGGTCGGTATCCACG
GAGTCCCAGCAGCCGACAAGAAGTACAGCATCGGCCTGGACATCGGCACCAACTCTGTGGGCTGGGCCGTGATCACCGACGAGTACAAGGTGCCCA
GCAAGAAATTCAAGGTGCTGGGCAACACCGACCGGCACAGCATCAAGAAGAACCTGATCGGAGCCCTGCTGTTCGACAGCGGCGAAACAGCCGAGG
CCACCCGGCTGAAGAGAACCGCCAGAAGAAGATACACCAGACGGAAGAACCGGATCTGCTATCTGCAAGAGATCTTCAGCAACGAGATGGCCAAGG
TGGACGACAGCTTCTTCCACAGACTGGAAGAGTCCTTCCTGGTGGAAGAGGATAAGAAGCACGAGCGGCACCCCATCTTCGGCAACATCGTGGACG
AGGTGGCCTACCACGAGAAGTACCCCACCATCTACCACCTGAGAAAGAAACTGGTGGACAGCACCGACAAGGCCGACCTGCGGCTGATCTATCTGG
CCCTGGCCCACATGATCAAGTTCCGGGGCCACTTCCTGATCGAGGGCGACCTGAACCCCGACAACAGCGACGTGGACAAGCTGTTCATCCAGCTGG
TGCAGACCTACAACCAGCTGTTCGAGGAAAACCCCATCAACGCCAGCGGCGTGGACGCCAAGGCCATCCTGTCTGCCAGACTGAGCAAGAGCAGAC
GGCTGGAAAATCTGATCGCCCAGCTGCCCGGCGAGAAGAAGAATGGCCTGTTCGGAAACCTGATTGCCCTGAGCCTGGGCCTGACCCCCAACTTCA
AGAGCAACTTCGACCTGGCCGAGGATGCCAAACTGCAGCTGAGCAAGGACACCTACGACGACGACCTGGACAACCTGCTGGCCCAGATCGGCGACC
AGTACGCCGACCTGTTTCTGGCCGCCAAGAACCTGTCCGACGCCATCCTGCTGAGCGACATCCTGAGAGTGAACACCGAGATCACCAAGGCCCCCC
TGAGCGCCTCTATGATCAAGAGATACGACGAGCACCACCAGGACCTGACCCTGCTGAAAGCTCTCGTGCGGCAGCAGCTGCCTGAGAAGTACAAAG
AGATTTTCTTCGACCAGAGCAAGAACGGCTACGCCGGCTACATTGACGGCGGAGCCAGCCAGGAAGAGTTCTACAAGTTCATCAAGCCCATCCTGG
AAAAGATGGACGGCACCGAGGAACTGCTCGTGAAGCTGAACAGAGAGGACCTGCTGCGGAAGCAGCGGACCTTCGACAACGGCAGCATCCCCCACC
AGATCCACCTGGGAGAGCTGCACGCCATTCTGCGGCGGCAGGAAGATTTTTACCCATTCCTGAAGGACAACCGGGAAAAGATCGAGAAGATCCTGA
CCTTCCGCATCCCCTACTACGTGGGCCCTCTGGCCAGGGGAAACAGCAGATTCGCCTGGATGACCAGAAAGAGCGAGGAAACCATCACCCCCTGGA
ACTTCGAGGAAGTGGTGGACAAGGGCGCTTCCGCCCAGAGCTTCATCGAGCGGATGACCAACTTCGATAAGAACCTGCCCAACGAGAAGGTGCTGC
CCAAGCACAGCCTGCTGTACGAGTACTTCACCGTGTATAACGAGCTGACCAAAGTGAAATACGTGACCGAGGGAATGAGAAAGCCCGCCTTCCTGA
GCGGCGAGCAGAAAAAGGCCATCGTGGACCTGCTGTTCAAGACCAACCGGAAAGTGACCGTGAAGCAGCTGAAAGAGGACTACTTCAAGAAAATCG
AGTGCTTCGACTCCGTGGAAATCTCCGGCGTGGAAGATCGGTTCAACGCCTCCCTGGGCACATACCACGATCTGCTGAAAATTATCAAGGACAAGG
ACTTCCTGGACAATGAGGAAAACGAGGACATTCTGGAAGATATCGTGCTGACCCTGACACTGTTTGAGGACAGAGAGATGATCGAGGAACGGCTGA
AAACCTATGCCCACCTGTTCGACGACAAAGTGATGAAGCAGCTGAAGCGGCGGAGATACACCGGCTGGGGCAGGCTGAGCCGGAAGCTGATCAACG
GCATCCGGGACAAGCAGTCCGGCAAGACAATCCTGGATTTCTGAAGTCCGACGGCTTCGCCAACAGAAACTTCATGCAGCTGATCCACGACGACAG
CGTGACCTTTAAAGAGGACATCCAGAAAGCCCCAGGTGTCCGGCCAGGGCGATAGCCTGCACGAGCACATTGCCAATCTGGCCGGCAGCCCCGCCA
TTAAGAAGGGCATCCTGCAGACAGTGAAGGTGGTGGACGAGCTCGTGAAAGTGATGGGCCGGCACAAGCCCGAGAACATCGTGATCGAAATGGCCA
GAGAGAACCAGACCACCCAGAAGGGACAGAAGAACAGCCGCGAGAGAATGAAGCGGATCGAAGAGGGCATCAAAGAGCTGGGCAGCCAGATCCTGA
AAGAACACCCCGTGGAAAACACCCAGCTGCAGAACGAGAAGCTGTACCTGTACTACCTGCAGAATGGGCGGGATATGTACGTGGACCAGGAACTGG
ACATCAACCGGCTGTCCGACTACGATGTGGACCATATCGTGCCTCAGAGCTTTCTGAAGGACGACTCCATCGACAACAAGGTGCTGACCAGAAGCG
ACAAGAACCGGGGCAAGAGCGACAACGTGCCCTCCGAAGAGGTCGTGAAGAAGATGAAGAACTACTGGCGGCAGCTGCTGAACGCCAAGCTGATTA
CCCAGAGAAAGTTCGACAATCTGACCAAGGCCCGAGAGAGGCGGCTGAGCGAACTGGATAAGGCCCGGCTTCATCAAGAGACAGCTGGTGGAAACC
CGGCAGATCACAAAGCACGTGGCACAGATCCTGGACTCCCGGATGAACACTAAGTACGACGAGAATGACAAGCTGATCCGGGAAGTGAAAGTGATC
ACCCTGAAGTCCAAGCTGGTGTCCGATTTCCGGAAGGATTTCCAGTTTTACAAAGTGCGCGAGATCAACAACTACCACCACGCCCACGACGCCTAC
CTGAACGCCGTCGTGGGAACCGCCCTGATCAAAAAGTACCCTAAGCTGGAAAGCGAGTTCGTGTACGGCGACTACAAGGTGTACGACGTGCGGAAG
ATGATCGCCAAGAGCGAGCAGGAAATCGGCAAGGCTACCGCCAAGTACTTCTTCTACAGCAACATCATGAACTTTTTCAAGACCGAGATTACCCTG
GCCAACGGCGAGATCCGGAAGCGGCCTCTGATCGAGACAAACGGCGAAACCGGGGAGATCGTGTGGGATAAGGGCCGGGATTTTGCCACCGTGCGG
AAAGTGCTGAGCATGCCCCAAGTGAATATCGTGAAAAAGACCGAGGTGCAGACAGGCGGCTTCAGCAAAGAGTCTATCCTGCCCAAGAGGAACAGC
GATAAGCTGATCGCCAGAAAGAAGGACTGGGACCCTAAGAAGTACGGCGGCTTCGACAGCCCCACCGTGGCCTATTCTGTGCTGGTGGTGGCCAAA
GTGGAAAAGGGCAAGTCCAAGAAACTGAAGAGTGTGAAAGAGCTGCTGGGGATCACCATCATGGAAAGAAGCAGCTTCGAGAAGAATCCCATCGAC
TTTCTGGAAGCCAAGGGCTACAAAGAAGTGAAAAAGGACCTGATCATCAAGCTGCCTAAGTACTCCCTGTTCGAGCTGGAAAACGGCCGGAAGAGA
ATGCTGGCCTCTGCCGGCGAACTGCAGAAGGGAAACGAACTGGCCCTGCCCTCCAAATATGTGAACTTCCTGTACCTGGCCAGCCACTATGAGAAG
CTGAAGGGCTCCCCCGAGGATAATGAGCAGAAACAGCTGTTTGTGGAACAGCACAAGCACTACCTGGACGAGATCATCGAGCAGATCAGCGAGTTC
TCCAAGAGAGTGATCCTGGCCGACGCTAATCTGGACAAAGTGCTGTCCGCCTACAACAAGCACCGGGATAAGCCCATCAGAGAGCAGGCCGAGAAT
ATCATCCACCTGTTTACCCTGACCAATCTGGGAGCCCCTGCCGCCTTCAAGTACTTTGACACCACCATCGACCGGAAGAGGTACACCAGCACCAAA
GAGGTGCTGGACGCCACCCTGATCCACCAGAGCATCACCGGCCTGTACGAGACACGGATCGACCTGTCTCAGCTGGGAGGCGACCCAAAGAAGAAG
CGGAAGGTCTGAAAGCTTGCGGCCGCACTCGAGCACCACCACCACCACCACTGAGATCCGGCTGCTAACAAAGCCCGAAAGAGCGATTTCC

6. The sequence obtained with the script is shown below.

>merged
GCTGACTCTTCCCTCTAGAATAATTTTGTTTAACTTTAAGAAGGAGATATACCATGGGCAGCAGCCATCATCATCATCATCACAGCAGCGGCCTGG
AAGTTCTGTTCCAGGGGCCCCATATGGCTAGCATGACTGGTGGACAGCAAATGGGTCGCGGATCCCCAAAGAAGAAGCGGAAGGTCGGTATCCACG
GAGTCCCAGCAGCCGACAAGAAGTACAGCATCGGCCTGGACATCGGCACCAACTCTGTGGGCTGGGCCGTGATCACCGACGAGTACAAGGTGCCCA
GCAAGAAATTCAAGGTGCTGGGCAACACCGACCGGCACAGCATCAAGAAGAACCTGATCGGAGCCCTGCTGTTCGACAGCGGCGAAACAGCCGAGG
CCACCCGGCTGAAGAGAACCGCCAGAAGAAGATACACCAGACGGAAGAACCGGATCTGCTATCTGCAAGAGATCTTCAGCAACGAGATGGCCAAGG
TGGACGACAGCTTCTTCCACAGACTGGAAGAGTCCTTCCTGGTGGAAGAGGATAAGAAGCACGAGCGGCACCCCATCTTCGGCAACATCGTGGACG
AGGTGGCCTACCACGAGAAGTACCCCACCATCTACCACCTGAGAAAGAAACTGGTGGACAGCACCGACAAGGCCGACCTGCGGCTGATCTATCTGG

```
CCCTGGCCCACATGATCAAGTTCCGGGGCCACTTCCTGATCGAGGGCGACCTGAACCCCGACAACAGCGACGTGGACAAGCTGTTCATCCAGCTGG
TGCAGACCTACAACCAGCTGTTCGAGGAAAACCCCATCAACGCCAGCGGCGTGGACGCCAAGGCCATCCTGTCTGCCAGACTGAGCAAGAGCAGAC
GGCTGGAAAATCTGATCGCCCAGCTGCCCGGCGAGAAGAAGAATGGCCTGTTCGGAAACCTGATTGCCCTGAGCCTGGGCCTGACCCCCAACTTCA
AGAGCAACTTCGACCTGGCCGAGGATGCCAAACTGCAGCTGAGCAAGGACACCTACGACGACGACCTGGACAACCTGCTGGCCCAGATCGGCGACC
AGTACGCCGACCTGTTTCTGGCCGCCAAGAACCTGTCCGACGCCATCCTGCTGAGCGACATCCTGAGAGTGAACACCGAGATCACCAAGGCCCCCC
TGAGCGCCTCTATGATCAAGAGATACGACGAGCACCACCAGGACCTGACCCTGCTGAAAGCTCTCGTGCGGCAGCAGCTGCCTGAGAAGTACAAAG
AGATTTTCTTCGACCAGAGCAAGAACGGCTACGCCGGCTACATTGACGGCGGAGCCAGCCAGGAAGAGTTCTACAAGTTCATCAAGCCCATCCTGG
AAAAGATGGACGGCACCGAGGAACTGCTCGTGAAGCTGAACAGAGAGGACCTGCTGCGGAAGCAGCGGACCTTCGACAACGGCAGCATCCCCCACC
AGATCCACCTGGGAGAGCTGCACGCCATTCTGCGGCGGCAGGAAGATTTTTACCCATTCCTGAAGGACAACCGGGAAAAGATCGAGAAGATCCTGA
CCTTCCGCATCCCCTACTACGTGGGCCCTCTGGCCAGGGGAAACAGCAGATTCGCCTGGATGACCAGAAAGAGCGAGGAAACCATCACCCCCTGGA
ACTTCGAGGAAGTGGTGGACAAGGGCGCTTCCGCCCAGAGCTTCATCGAGCGGATGACCAACTTCGATAAGAACCTGCCCAACGAGAAGGTGCTGC
CCAAGCACAGCCTGCTGTACGAGTACTTCACCGTGTATAACGAGCTGACCAAAGTGAAATACGTGACCGAGGGAATGAGAAAGCCCGCCTTCCTGA
GCGGCGAGCAGAAAAAGGCCATCGTGGACCTGCTGTTCAAGACCAACCGGAAAGTGACCGTGAAGCAGCTGAAAGAGGACTACTTCAAGAAAATCG
AGTGCTTCGACTCCGTGGAAATCTCCGGCGTGGAAGATCGGTTCAACGCCTCCCTGGGCACATACCACGATCTGCTGAAAATTATCAAGGACAAGG
ACTTCCTGGACAATGAGGAAAACGAGGACATTCTGGAAGATATCGTGCTGACCCTGACACTGTTTGAGGACAGAGAGATGATCGAGGAACGGCTGA
AAACCTATGCCCACCTGTTCGACGACAAAGTGATGAAGCAGCTGAAGCGGCGGAGATACACCGGCTGGGGCAGGCTGAGCCGGAAGCTGATCAACG
GCATCCGGGACAAGCAGTCCGGCAAGACAATCCTGGATTTCTGAAGTCCGACGGCTTCGCCAACAGAAACTTCATGCAGCTGATCCACGACGACAG
CGTGACCTTTAAAGAGGACATCCAGAAAGCCCAGGTGTCCGGCCAGGGCGATAGCCTGCACGAGCACATTGCCAATCTGGCCGGCAGCCCCGCCA
TTAAGAAGGGCATCCTGCAGACAGTGAAGGTGGTGGACGAGCTCGTGAAAGTGATGGGCCGGCACAAGCCCGAGAACATCGTGATCGAAATGGCCA
GAGAGAACCAGACCACCCAGAAGGGACAGAAGAACAGCCGCGAGAGAATGAAGCGGATCGAAGAGGGCATCAAAGAGCTGGGCAGCCAGATCCTGA
AAGAACACCCCGTGGAAAACACCCAGCTGCAGAACGAGAAGCTGTACCTGTACTACCTGCAGAATGGGCGGGATATGTACGTGGACCAGGAACTGG
ACATCAACCGGCTGTCCGACTACGATGTGGACCATATCGTGCCTCAGAGCTTTCTGAAGGACGACTCCATCGACAACAAGGTGCTGACCAGAAGCG
ACAAGAACCGGGGCAAGAGCGACAACGTGCCCTCCGAAGAGGTCGTGAAGAAGATGAAGAACTACTGGCGGCAGCTGCTGAACGCCAAGCTGATTA
CCCAGAGAAAGTTCGACAATCTGACCAAGGCCCGAGAGAGGCGGCTGAGCGAACTGGATAAGGCCCGGCTTCATCAAGAGACAGCTGGTGGAAACC
CGGCAGATCACAAAGCACGTGGCACAGATCCTGGACTCCCGGATGAACACTAAGTACGACGAGAATGACAAGCTGATCCGGGAAGTGAAAGTGATC
ACCCTGAAGTCCAAGCTGGTGTCCGATTTCCGGAAGGATTTCCAGTTTTACAAAGTGCGCGAGATCAACAACTACCACCACGCCCACGACGCCTAC
CTGAACGCCGTCGTGGGAACCGCCCTGATCAAAAAGTACCCTAAGCTGGAAAGCGAGTTCGTGTACGGCGACTACAAGGTGTACGACGTGCGGAAG
ATGATCGCCAAGAGCGAGCAGGAAATCGGCAAGGCTACCGCCAAGTACTTCTTCTACAGCAACATCATGAACTTTTTCAAGACCGAGATTACCCTG
GCCAACGGCGAGATCCGGAAGCGGCCTCTGATCGAGACAAACGGCGAAACCGGGGAGATCGTGTGGGATAAGGGCCGGGATTTTGCCACCGTGCGG
AAAGTGCTGAGCATGCCCCAAGTGAATATCGTGAAAAAGACCGAGGTGCAGACAGGCGGCTTCAGCAAAGAGTCTATCCTGCCCAAGAGGAACAGC
GATAAGCTGATCGCCAGAAAGAAGGACTGGGACCCTAAGAAGTACGGCGGCTTCGACAGCCCCACCGTGGCCTATTCTGTGCTGGTGGTGGCCAAA
GTGGAAAAGGGCAAGTCCAAGAAACTGAAGAGTGTGAAAGAGCTGCTGGGGATCACCATCATGGAAAGAAGCAGCTTCGAGAAGAATCCCATCGAC
TTTCTGGAAGCCAAGGGCTACAAAGAAGTGAAAAAGGACCTGATCATCAAGCTGCCTAAGTACTCCCTGTTCGAGCTGGAAAACGGCCGGAAGAGA
ATGCTGGCCTCTGCCGGCGAACTGCAGAAGGGAAACGAACTGGCCCTGCCCTCCAAATATGTGAACTTCCTGTACCTGGCCAGCCACTATGAGAAG
CTGAAGGGCTCCCCCGAGGATAATGAGCAGAAACAGCTGTTTGTGGAACAGCACAAGCACTACCTGGACGAGATCATCGAGCAGATCAGCGAGTTC
TCCAAGAGAGTGATCCTGGCCGACGCTAATCTGGACAAAGTGCTGTCCGCCTACAACAAGCACCGGGATAAGCCCATCAGAGAGCAGGCCGAGAAT
ATCATCCACCTGTTTACCCTGACCAATCTGGGAGCCCCTGCCGCCTTCAAGTACTTTGACACCACCATCGACCGGAAGAGGTACACCAGCACCAAA
GAGGTGCTGGACGCCACCCTGATCCACCAGAGCATCACCGGCCTGTACGAGACACGGATCGACCTGTCTCAGCTGGGAGGCGACCCAAAGAAGAAG
CGGAAGGTCTGAAAGCTTGCGGCCGCACTCGAGCACCACCACCACCACCACTGAGATCCGGCTGCTAACAAAGCCCGAAAGAGCGATTTCC
```

7. The manual joined sequence is aligned to the script merged sequence with EMBOSS needle and the results is shown below. As can be seen, the two sequence are identical.

```
gene               1 GCTGACTCTTCCCTCTAGAATAATTTTGTTTAACTTTAAGAAGGAGATAT     50
                     ||||||||||||||||||||||||||||||||||||||||||||||||||
merged             1 GCTGACTCTTCCCTCTAGAATAATTTTGTTTAACTTTAAGAAGGAGATAT     50

gene              51 ACCATGGGCAGCAGCCATCATCATCATCATCACAGCAGCGGCCTGGAAGT    100
                     ||||||||||||||||||||||||||||||||||||||||||||||||||
merged            51 ACCATGGGCAGCAGCCATCATCATCATCATCACAGCAGCGGCCTGGAAGT    100

gene             101 TCTGTTCCAGGGGCCCCATATGGCTAGCATGACTGGTGGACAGCAAATGG    150
                     ||||||||||||||||||||||||||||||||||||||||||||||||||
merged           101 TCTGTTCCAGGGGCCCCATATGGCTAGCATGACTGGTGGACAGCAAATGG    150

gene             151 GTCGCGGATCCCCAAAGAAGAAGCGGAAGGTCGGTATCCACGGAGTCCCA    200
                     ||||||||||||||||||||||||||||||||||||||||||||||||||
merged           151 GTCGCGGATCCCCAAAGAAGAAGCGGAAGGTCGGTATCCACGGAGTCCCA    200

gene             201 GCAGCCGACAAGAAGTACAGCATCGGCCTGGACATCGGCACCAACTCTGT    250
                     ||||||||||||||||||||||||||||||||||||||||||||||||||
merged           201 GCAGCCGACAAGAAGTACAGCATCGGCCTGGACATCGGCACCAACTCTGT    250

gene             251 GGGCTGGGCCGTGATCACCGACGAGTACAAGGTGCCCAGCAAGAAATTCA    300
                     ||||||||||||||||||||||||||||||||||||||||||||||||||
merged           251 GGGCTGGGCCGTGATCACCGACGAGTACAAGGTGCCCAGCAAGAAATTCA    300

gene             301 AGGTGCTGGGCAACACCGACCGGCACAGCATCAAGAAGAACCTGATCGGA    350
                     ||||||||||||||||||||||||||||||||||||||||||||||||||
merged           301 AGGTGCTGGGCAACACCGACCGGCACAGCATCAAGAAGAACCTGATCGGA    350

gene             351 GCCCTGCTGTTCGACAGCGGCGAAACAGCCGAGGCCACCCGGCTGAAGAG    400
                     ||||||||||||||||||||||||||||||||||||||||||||||||||
merged           351 GCCCTGCTGTTCGACAGCGGCGAAACAGCCGAGGCCACCCGGCTGAAGAG    400
```

```
gene              401 AACCGCCAGAAGAAGATACACCAGACGGAAGAACCGGATCTGCTATCTGC    450
                      ||||||||||||||||||||||||||||||||||||||||||||||||||
merged            401 AACCGCCAGAAGAAGATACACCAGACGGAAGAACCGGATCTGCTATCTGC    450

gene              451 AAGAGATCTTCAGCAACGAGATGGCCAAGGTGGACGACAGCTTCTTCCAC    500
                      ||||||||||||||||||||||||||||||||||||||||||||||||||
merged            451 AAGAGATCTTCAGCAACGAGATGGCCAAGGTGGACGACAGCTTCTTCCAC    500

gene              501 AGACTGGAAGAGTCCTTCCTGGTGGAAGAGGATAAGAAGCACGAGCGGCA    550
                      ||||||||||||||||||||||||||||||||||||||||||||||||||
merged            501 AGACTGGAAGAGTCCTTCCTGGTGGAAGAGGATAAGAAGCACGAGCGGCA    550

gene              551 CCCCATCTTCGGCAACATCGTGGACGAGGTGGCCTACCACGAGAAGTACC    600
                      ||||||||||||||||||||||||||||||||||||||||||||||||||
merged            551 CCCCATCTTCGGCAACATCGTGGACGAGGTGGCCTACCACGAGAAGTACC    600

gene              601 CCACCATCTACCACCTGAGAAAGAAACTGGTGGACAGCACCGACAAGGCC    650
                      ||||||||||||||||||||||||||||||||||||||||||||||||||
merged            601 CCACCATCTACCACCTGAGAAAGAAACTGGTGGACAGCACCGACAAGGCC    650

gene              651 GACCTGCGGCTGATCTATCTGGCCCTGGCCCACATGATCAAGTTCCGGGG    700
                      ||||||||||||||||||||||||||||||||||||||||||||||||||
merged            651 GACCTGCGGCTGATCTATCTGGCCCTGGCCCACATGATCAAGTTCCGGGG    700

gene              701 CCACTTCCTGATCGAGGGCGACCTGAACCCCGACAACAGCGACGTGGACA    750
                      ||||||||||||||||||||||||||||||||||||||||||||||||||
merged            701 CCACTTCCTGATCGAGGGCGACCTGAACCCCGACAACAGCGACGTGGACA    750

gene              751 AGCTGTTCATCCAGCTGGTGCAGACCTACAACCAGCTGTTCGAGGAAAAC    800
                      ||||||||||||||||||||||||||||||||||||||||||||||||||
merged            751 AGCTGTTCATCCAGCTGGTGCAGACCTACAACCAGCTGTTCGAGGAAAAC    800

gene              801 CCCATCAACGCCAGCGGCGTGGACGCCAAGGCCATCCTGTCTGCCAGACT    850
                      ||||||||||||||||||||||||||||||||||||||||||||||||||
merged            801 CCCATCAACGCCAGCGGCGTGGACGCCAAGGCCATCCTGTCTGCCAGACT    850

gene              851 GAGCAAGAGCAGACGGCTGGAAAATCTGATCGCCCAGCTGCCCGGCGAGA    900
                      ||||||||||||||||||||||||||||||||||||||||||||||||||
merged            851 GAGCAAGAGCAGACGGCTGGAAAATCTGATCGCCCAGCTGCCCGGCGAGA    900

gene              901 AGAAGAATGGCCTGTTCGGAAACCTGATTGCCCTGAGCCTGGGCCTGACC    950
                      ||||||||||||||||||||||||||||||||||||||||||||||||||
merged            901 AGAAGAATGGCCTGTTCGGAAACCTGATTGCCCTGAGCCTGGGCCTGACC    950

gene              951 CCCAACTTCAAGAGCAACTTCGACCTGGCCGAGGATGCCAAACTGCAGCT   1000
                      ||||||||||||||||||||||||||||||||||||||||||||||||||
merged            951 CCCAACTTCAAGAGCAACTTCGACCTGGCCGAGGATGCCAAACTGCAGCT   1000

gene             1001 GAGCAAGGACACCTACGACGACGACCTGGACAACCTGCTGGCCCAGATCG   1050
                      ||||||||||||||||||||||||||||||||||||||||||||||||||
merged           1001 GAGCAAGGACACCTACGACGACGACCTGGACAACCTGCTGGCCCAGATCG   1050

gene             1051 GCGACCAGTACGCCGACCTGTTTCTGGCCGCCAAGAACCTGTCCGACGCC   1100
                      ||||||||||||||||||||||||||||||||||||||||||||||||||
merged           1051 GCGACCAGTACGCCGACCTGTTTCTGGCCGCCAAGAACCTGTCCGACGCC   1100

gene             1101 ATCCTGCTGAGCGACATCCTGAGAGTGAACACCGAGATCACCAAGGCCCC   1150
                      ||||||||||||||||||||||||||||||||||||||||||||||||||
merged           1101 ATCCTGCTGAGCGACATCCTGAGAGTGAACACCGAGATCACCAAGGCCCC   1150

gene             1151 CCTGAGCGCCTCTATGATCAAGAGATACGACGAGCACCACCAGGACCTGA   1200
                      ||||||||||||||||||||||||||||||||||||||||||||||||||
merged           1151 CCTGAGCGCCTCTATGATCAAGAGATACGACGAGCACCACCAGGACCTGA   1200

gene             1201 CCCTGCTGAAAGCTCTCGTGCGGCAGCAGCTGCCTGAGAAGTACAAAGAG   1250
                      ||||||||||||||||||||||||||||||||||||||||||||||||||
merged           1201 CCCTGCTGAAAGCTCTCGTGCGGCAGCAGCTGCCTGAGAAGTACAAAGAG   1250

gene             1251 ATTTTCTTCGACCAGAGCAAGAACGGCTACGCCGGCTACATTGACGGCGG   1300
                      ||||||||||||||||||||||||||||||||||||||||||||||||||
merged           1251 ATTTTCTTCGACCAGAGCAAGAACGGCTACGCCGGCTACATTGACGGCGG   1300

gene             1301 AGCCAGCCAGGAAGAGTTCTACAAGTTCATCAAGCCCATCCTGGAAAAGA   1350
                      ||||||||||||||||||||||||||||||||||||||||||||||||||
```

```
merged         1301 AGCCAGCCAGGAAGAGTTCTACAAGTTCATCAAGCCCATCCTGGAAAAGA   1350

gene           1351 TGGACGGCACCGAGGAACTGCTCGTGAAGCTGAACAGAGAGGACCTGCTG   1400
                    ||||||||||||||||||||||||||||||||||||||||||||||||||
merged         1351 TGGACGGCACCGAGGAACTGCTCGTGAAGCTGAACAGAGAGGACCTGCTG   1400

gene           1401 CGGAAGCAGCGGACCTTCGACAACGGCAGCATCCCCCACCAGATCCACCT   1450
                    ||||||||||||||||||||||||||||||||||||||||||||||||||
merged         1401 CGGAAGCAGCGGACCTTCGACAACGGCAGCATCCCCCACCAGATCCACCT   1450

gene           1451 GGGAGAGCTGCACGCCATTCTGCGGCGGCAGGAAGATTTTTACCCATTCC   1500
                    ||||||||||||||||||||||||||||||||||||||||||||||||||
merged         1451 GGGAGAGCTGCACGCCATTCTGCGGCGGCAGGAAGATTTTTACCCATTCC   1500

gene           1501 TGAAGGACAACCGGGAAAAGATCGAGAAGATCCTGACCTTCCGCATCCCC   1550
                    ||||||||||||||||||||||||||||||||||||||||||||||||||
merged         1501 TGAAGGACAACCGGGAAAAGATCGAGAAGATCCTGACCTTCCGCATCCCC   1550

gene           1551 TACTACGTGGGCCCTCTGGCCAGGGGAAACAGCAGATTCGCCTGGATGAC   1600
                    ||||||||||||||||||||||||||||||||||||||||||||||||||
merged         1551 TACTACGTGGGCCCTCTGGCCAGGGGAAACAGCAGATTCGCCTGGATGAC   1600

gene           1601 CAGAAAGAGCGAGGAAACCATCACCCCCTGGAACTTCGAGGAAGTGGTGG   1650
                    ||||||||||||||||||||||||||||||||||||||||||||||||||
merged         1601 CAGAAAGAGCGAGGAAACCATCACCCCCTGGAACTTCGAGGAAGTGGTGG   1650

gene           1651 ACAAGGGCGCTTCCGCCCAGAGCTTCATCGAGCGGATGACCAACTTCGAT   1700
                    ||||||||||||||||||||||||||||||||||||||||||||||||||
merged         1651 ACAAGGGCGCTTCCGCCCAGAGCTTCATCGAGCGGATGACCAACTTCGAT   1700

gene           1701 AAGAACCTGCCCAACGAGAAGGTGCTGCCCAAGCACAGCCTGCTGTACGA   1750
                    ||||||||||||||||||||||||||||||||||||||||||||||||||
merged         1701 AAGAACCTGCCCAACGAGAAGGTGCTGCCCAAGCACAGCCTGCTGTACGA   1750

gene           1751 GTACTTCACCGTGTATAACGAGCTGACCAAAGTGAAATACGTGACCGAGG   1800
                    ||||||||||||||||||||||||||||||||||||||||||||||||||
merged         1751 GTACTTCACCGTGTATAACGAGCTGACCAAAGTGAAATACGTGACCGAGG   1800

gene           1801 GAATGAGAAAGCCCGCCTTCCTGAGCGGCGAGCAGAAAAAGGCCATCGTG   1850
                    ||||||||||||||||||||||||||||||||||||||||||||||||||
merged         1801 GAATGAGAAAGCCCGCCTTCCTGAGCGGCGAGCAGAAAAAGGCCATCGTG   1850

gene           1851 GACCTGCTGTTCAAGACCAACCGGAAAGTGACCGTGAAGCAGCTGAAAGA   1900
                    ||||||||||||||||||||||||||||||||||||||||||||||||||
merged         1851 GACCTGCTGTTCAAGACCAACCGGAAAGTGACCGTGAAGCAGCTGAAAGA   1900

gene           1901 GGACTACTTCAAGAAAATCGAGTGCTTCGACTCCGTGGAAATCTCCGGCG   1950
                    ||||||||||||||||||||||||||||||||||||||||||||||||||
merged         1901 GGACTACTTCAAGAAAATCGAGTGCTTCGACTCCGTGGAAATCTCCGGCG   1950

gene           1951 TGGAAGATCGGTTCAACGCCTCCCTGGGCACATACCACGATCTGCTGAAA   2000
                    ||||||||||||||||||||||||||||||||||||||||||||||||||
merged         1951 TGGAAGATCGGTTCAACGCCTCCCTGGGCACATACCACGATCTGCTGAAA   2000

gene           2001 ATTATCAAGGACAAGGACTTCCTGGACAATGAGGAAAACGAGGACATTCT   2050
                    ||||||||||||||||||||||||||||||||||||||||||||||||||
merged         2001 ATTATCAAGGACAAGGACTTCCTGGACAATGAGGAAAACGAGGACATTCT   2050

gene           2051 GGAAGATATCGTGCTGACCCTGACACTGTTTGAGGACAGAGAGATGATCG   2100
                    ||||||||||||||||||||||||||||||||||||||||||||||||||
merged         2051 GGAAGATATCGTGCTGACCCTGACACTGTTTGAGGACAGAGAGATGATCG   2100

gene           2101 AGGAACGGCTGAAAACCTATGCCCACCTGTTCGACGACAAAGTGATGAAG   2150
                    ||||||||||||||||||||||||||||||||||||||||||||||||||
merged         2101 AGGAACGGCTGAAAACCTATGCCCACCTGTTCGACGACAAAGTGATGAAG   2150

gene           2151 CAGCTGAAGCGGCGGAGATACACCGGCTGGGGCAGGCTGAGCCGGAAGCT   2200
                    ||||||||||||||||||||||||||||||||||||||||||||||||||
merged         2151 CAGCTGAAGCGGCGGAGATACACCGGCTGGGGCAGGCTGAGCCGGAAGCT   2200

gene           2201 GATCAACGGCATCCGGGACAAGCAGTCCGGCAAGACAATCCTGGATTTCT   2250
                    ||||||||||||||||||||||||||||||||||||||||||||||||||
merged         2201 GATCAACGGCATCCGGGACAAGCAGTCCGGCAAGACAATCCTGGATTTCT   2250

gene           2251 GAAGTCCGACGGCTTCGCCAACAGAAACTTCATGCAGCTGATCCACGACG   2300
```

```
                     ||||||||||||||||||||||||||||||||||||||||||||||||||
merged          2251 GAAGTCCGACGGCTTCGCCAACAGAAACTTCATGCAGCTGATCCACGACG   2300

gene            2301 ACAGCGTGACCTTTAAAGAGGACATCCAGAAAGCCCCAGGTGTCCGGCCA   2350
                     ||||||||||||||||||||||||||||||||||||||||||||||||||
merged          2301 ACAGCGTGACCTTTAAAGAGGACATCCAGAAAGCCCCAGGTGTCCGGCCA   2350

gene            2351 GGGCGATAGCCTGCACGAGCACATTGCCAATCTGGCCGGCAGCCCCGCCA   2400
                     ||||||||||||||||||||||||||||||||||||||||||||||||||
merged          2351 GGGCGATAGCCTGCACGAGCACATTGCCAATCTGGCCGGCAGCCCCGCCA   2400

gene            2401 TTAAGAAGGGCATCCTGCAGACAGTGAAGGTGGTGGACGAGCTCGTGAAA   2450
                     ||||||||||||||||||||||||||||||||||||||||||||||||||
merged          2401 TTAAGAAGGGCATCCTGCAGACAGTGAAGGTGGTGGACGAGCTCGTGAAA   2450

gene            2451 GTGATGGGCCGGCACAAGCCCGAGAACATCGTGATCGAAATGGCCAGAGA   2500
                     ||||||||||||||||||||||||||||||||||||||||||||||||||
merged          2451 GTGATGGGCCGGCACAAGCCCGAGAACATCGTGATCGAAATGGCCAGAGA   2500

gene            2501 GAACCAGACCACCCAGAAGGGACAGAAGAACAGCCGCGAGAGAATGAAGC   2550
                     ||||||||||||||||||||||||||||||||||||||||||||||||||
merged          2501 GAACCAGACCACCCAGAAGGGACAGAAGAACAGCCGCGAGAGAATGAAGC   2550

gene            2551 GGATCGAAGAGGGCATCAAAGAGCTGGGCAGCCAGATCCTGAAAGAACAC   2600
                     ||||||||||||||||||||||||||||||||||||||||||||||||||
merged          2551 GGATCGAAGAGGGCATCAAAGAGCTGGGCAGCCAGATCCTGAAAGAACAC   2600

gene            2601 CCCGTGGAAAACACCCAGCTGCAGAACGAGAAGCTGTACCTGTACTACCT   2650
                     ||||||||||||||||||||||||||||||||||||||||||||||||||
merged          2601 CCCGTGGAAAACACCCAGCTGCAGAACGAGAAGCTGTACCTGTACTACCT   2650

gene            2651 GCAGAATGGGCGGGATATGTACGTGGACCAGGAACTGGACATCAACCGGC   2700
                     ||||||||||||||||||||||||||||||||||||||||||||||||||
merged          2651 GCAGAATGGGCGGGATATGTACGTGGACCAGGAACTGGACATCAACCGGC   2700

gene            2701 TGTCCGACTACGATGTGGACCATATCGTGCCTCAGAGCTTTCTGAAGGAC   2750
                     ||||||||||||||||||||||||||||||||||||||||||||||||||
merged          2701 TGTCCGACTACGATGTGGACCATATCGTGCCTCAGAGCTTTCTGAAGGAC   2750

gene            2751 GACTCCATCGACAACAAGGTGCTGACCAGAAGCGACAAGAACCGGGGCAA   2800
                     ||||||||||||||||||||||||||||||||||||||||||||||||||
merged          2751 GACTCCATCGACAACAAGGTGCTGACCAGAAGCGACAAGAACCGGGGCAA   2800

gene            2801 GAGCGACAACGTGCCCTCCGAAGAGGTCGTGAAGAAGATGAAGAACTACT   2850
                     ||||||||||||||||||||||||||||||||||||||||||||||||||
merged          2801 GAGCGACAACGTGCCCTCCGAAGAGGTCGTGAAGAAGATGAAGAACTACT   2850

gene            2851 GGCGGCAGCTGCTGAACGCCAAGCTGATTACCCAGAGAAAGTTCGACAAT   2900
                     ||||||||||||||||||||||||||||||||||||||||||||||||||
merged          2851 GGCGGCAGCTGCTGAACGCCAAGCTGATTACCCAGAGAAAGTTCGACAAT   2900

gene            2901 CTGACCAAGGCCCGAGAGAGGCGGCTGAGCGAACTGGATAAGGCCCGGCT   2950
                     ||||||||||||||||||||||||||||||||||||||||||||||||||
merged          2901 CTGACCAAGGCCCGAGAGAGGCGGCTGAGCGAACTGGATAAGGCCCGGCT   2950

gene            2951 TCATCAAGAGACAGCTGGTGGAAACCCGGCAGATCACAAAGCACGTGGCA   3000
                     ||||||||||||||||||||||||||||||||||||||||||||||||||
merged          2951 TCATCAAGAGACAGCTGGTGGAAACCCGGCAGATCACAAAGCACGTGGCA   3000

gene            3001 CAGATCCTGGACTCCCGGATGAACACTAAGTACGACGAGAATGACAAGCT   3050
                     ||||||||||||||||||||||||||||||||||||||||||||||||||
merged          3001 CAGATCCTGGACTCCCGGATGAACACTAAGTACGACGAGAATGACAAGCT   3050

gene            3051 GATCCGGGAAGTGAAAGTGATCACCCTGAAGTCCAAGCTGGTGTCCGATT   3100
                     ||||||||||||||||||||||||||||||||||||||||||||||||||
merged          3051 GATCCGGGAAGTGAAAGTGATCACCCTGAAGTCCAAGCTGGTGTCCGATT   3100

gene            3101 TCCGGAAGGATTTCCAGTTTTACAAAGTGCGCGAGATCAACAACTACCAC   3150
                     ||||||||||||||||||||||||||||||||||||||||||||||||||
merged          3101 TCCGGAAGGATTTCCAGTTTTACAAAGTGCGCGAGATCAACAACTACCAC   3150

gene            3151 CACGCCCACGACGCCTACCTGAACGCCGTCGTGGGAACCGCCCTGATCAA   3200
                     ||||||||||||||||||||||||||||||||||||||||||||||||||
merged          3151 CACGCCCACGACGCCTACCTGAACGCCGTCGTGGGAACCGCCCTGATCAA   3200
```

```
gene            3201 AAAGTACCCTAAGCTGGAAAGCGAGTTCGTGTACGGCGACTACAAGGTGT   3250
                     ||||||||||||||||||||||||||||||||||||||||||||||||||
merged          3201 AAAGTACCCTAAGCTGGAAAGCGAGTTCGTGTACGGCGACTACAAGGTGT   3250

gene            3251 ACGACGTGCGGAAGATGATCGCCAAGAGCGAGCAGGAAATCGGCAAGGCT   3300
                     ||||||||||||||||||||||||||||||||||||||||||||||||||
merged          3251 ACGACGTGCGGAAGATGATCGCCAAGAGCGAGCAGGAAATCGGCAAGGCT   3300

gene            3301 ACCGCCAAGTACTTCTTCTACAGCAACATCATGAACTTTTTCAAGACCGA   3350
                     ||||||||||||||||||||||||||||||||||||||||||||||||||
merged          3301 ACCGCCAAGTACTTCTTCTACAGCAACATCATGAACTTTTTCAAGACCGA   3350

gene            3351 GATTACCCTGGCCAACGGCGAGATCCGGAAGCGGCCTCTGATCGAGACAA   3400
                     ||||||||||||||||||||||||||||||||||||||||||||||||||
merged          3351 GATTACCCTGGCCAACGGCGAGATCCGGAAGCGGCCTCTGATCGAGACAA   3400

gene            3401 ACGGCGAAACCGGGGAGATCGTGTGGGATAAGGGCCGGGATTTTGCCACC   3450
                     ||||||||||||||||||||||||||||||||||||||||||||||||||
merged          3401 ACGGCGAAACCGGGGAGATCGTGTGGGATAAGGGCCGGGATTTTGCCACC   3450

gene            3451 GTGCGGAAAGTGCTGAGCATGCCCCAAGTGAATATCGTGAAAAAGACCGA   3500
                     ||||||||||||||||||||||||||||||||||||||||||||||||||
merged          3451 GTGCGGAAAGTGCTGAGCATGCCCCAAGTGAATATCGTGAAAAAGACCGA   3500

gene            3501 GGTGCAGACAGGCGGCTTCAGCAAAGAGTCTATCCTGCCCAAGAGGAACA   3550
                     ||||||||||||||||||||||||||||||||||||||||||||||||||
merged          3501 GGTGCAGACAGGCGGCTTCAGCAAAGAGTCTATCCTGCCCAAGAGGAACA   3550

gene            3551 GCGATAAGCTGATCGCCAGAAAGAAGGACTGGGACCCTAAGAAGTACGGC   3600
                     ||||||||||||||||||||||||||||||||||||||||||||||||||
merged          3551 GCGATAAGCTGATCGCCAGAAAGAAGGACTGGGACCCTAAGAAGTACGGC   3600

gene            3601 GGCTTCGACAGCCCCACCGTGGCCTATTCTGTGCTGGTGGTGGCCAAAGT   3650
                     ||||||||||||||||||||||||||||||||||||||||||||||||||
merged          3601 GGCTTCGACAGCCCCACCGTGGCCTATTCTGTGCTGGTGGTGGCCAAAGT   3650

gene            3651 GGAAAAGGGCAAGTCCAAGAAACTGAAGAGTGTGAAAGAGCTGCTGGGGA   3700
                     ||||||||||||||||||||||||||||||||||||||||||||||||||
merged          3651 GGAAAAGGGCAAGTCCAAGAAACTGAAGAGTGTGAAAGAGCTGCTGGGGA   3700

gene            3701 TCACCATCATGGAAAGAAGCAGCTTCGAGAAGAATCCCATCGACTTTCTG   3750
                     ||||||||||||||||||||||||||||||||||||||||||||||||||
merged          3701 TCACCATCATGGAAAGAAGCAGCTTCGAGAAGAATCCCATCGACTTTCTG   3750

gene            3751 GAAGCCAAGGGCTACAAAGAAGTGAAAAAGGACCTGATCATCAAGCTGCC   3800
                     ||||||||||||||||||||||||||||||||||||||||||||||||||
merged          3751 GAAGCCAAGGGCTACAAAGAAGTGAAAAAGGACCTGATCATCAAGCTGCC   3800

gene            3801 TAAGTACTCCCTGTTCGAGCTGGAAAACGGCCGGAAGAGAATGCTGGCCT   3850
                     ||||||||||||||||||||||||||||||||||||||||||||||||||
merged          3801 TAAGTACTCCCTGTTCGAGCTGGAAAACGGCCGGAAGAGAATGCTGGCCT   3850

gene            3851 CTGCCGGCGAACTGCAGAAGGGAAACGAACTGGCCCTGCCCTCCAAATAT   3900
                     ||||||||||||||||||||||||||||||||||||||||||||||||||
merged          3851 CTGCCGGCGAACTGCAGAAGGGAAACGAACTGGCCCTGCCCTCCAAATAT   3900

gene            3901 GTGAACTTCCTGTACCTGGCCAGCCACTATGAGAAGCTGAAGGGCTCCCC   3950
                     ||||||||||||||||||||||||||||||||||||||||||||||||||
merged          3901 GTGAACTTCCTGTACCTGGCCAGCCACTATGAGAAGCTGAAGGGCTCCCC   3950

gene            3951 CGAGGATAATGAGCAGAAACAGCTGTTTGTGGAACAGCACAAGCACTACC   4000
                     ||||||||||||||||||||||||||||||||||||||||||||||||||
merged          3951 CGAGGATAATGAGCAGAAACAGCTGTTTGTGGAACAGCACAAGCACTACC   4000

gene            4001 TGGACGAGATCATCGAGCAGATCAGCGAGTTCTCCAAGAGAGTGATCCTG   4050
                     ||||||||||||||||||||||||||||||||||||||||||||||||||
merged          4001 TGGACGAGATCATCGAGCAGATCAGCGAGTTCTCCAAGAGAGTGATCCTG   4050

gene            4051 GCCGACGCTAATCTGGACAAAGTGCTGTCCGCCTACAACAAGCACCGGGA   4100
                     ||||||||||||||||||||||||||||||||||||||||||||||||||
merged          4051 GCCGACGCTAATCTGGACAAAGTGCTGTCCGCCTACAACAAGCACCGGGA   4100

gene            4101 TAAGCCCATCAGAGAGCAGGCCGAGAATATCATCCACCTGTTTACCCTGA   4150
                     ||||||||||||||||||||||||||||||||||||||||||||||||||
merged          4101 TAAGCCCATCAGAGAGCAGGCCGAGAATATCATCCACCTGTTTACCCTGA   4150
```

```
gene             4151 CCAATCTGGGAGCCCCTGCCGCCTTCAAGTACTTTGACACCACCATCGAC   4200
                      ||||||||||||||||||||||||||||||||||||||||||||||||||
merged           4151 CCAATCTGGGAGCCCCTGCCGCCTTCAAGTACTTTGACACCACCATCGAC   4200

gene             4201 CGGAAGAGGTACACCAGCACCAAAGAGGTGCTGGACGCCACCCTGATCCA   4250
                      ||||||||||||||||||||||||||||||||||||||||||||||||||
merged           4201 CGGAAGAGGTACACCAGCACCAAAGAGGTGCTGGACGCCACCCTGATCCA   4250

gene             4251 CCAGAGCATCACCGGCCTGTACGAGACACGGATCGACCTGTCTCAGCTGG   4300
                      ||||||||||||||||||||||||||||||||||||||||||||||||||
merged           4251 CCAGAGCATCACCGGCCTGTACGAGACACGGATCGACCTGTCTCAGCTGG   4300

gene             4301 GAGGCGACCCAAAGAAGAAGCGGAAGGTCTGAAAGCTTGCGGCCGCACTC   4350
                      ||||||||||||||||||||||||||||||||||||||||||||||||||
merged           4301 GAGGCGACCCAAAGAAGAAGCGGAAGGTCTGAAAGCTTGCGGCCGCACTC   4350

gene             4351 GAGCACCACCACCACCACCACTGAGATCCGGCTGCTAACAAAGCCCGAAA   4400
                      ||||||||||||||||||||||||||||||||||||||||||||||||||
merged           4351 GAGCACCACCACCACCACCACTGAGATCCGGCTGCTAACAAAGCCCGAAA   4400

gene             4401 GAGCGATTTCC   4411
                      |||||||||||
merged           4401 GAGCGATTTCC   4411
```
