# Supplementary material for: A Python script to merge Sanger sequences
Source: PeerJ. 2021 Apr 27;9:e11354. doi: 10.7717/peerj.11354 (PMC8086567; doi:10.7717/peerj.11354)
Supplement: Supplemental Information 10 [file peerj-09-11354-s010.pdf]

```
MBP:example_input_files lsq$ python3.7 Merge_Sanger_v2.py 001F_sequence_to_be_merged.seq
002F_sequence_to_be_merged.seq  003F_sequence_to_be_merged.seq 004R_sequence_to_be_merged.seq
005R_sequence_to_be_merged.seq 006R_sequence_to_be_merged.seq
(^_^)
(^_^)(^_^)(^_^)
(^_^)(^_^)(^_^)(^_^)(^_^)
(^_^)(^_^)(^_^)(^_^)(^_^)(^_^)(^_^)
(^_^)(^_^)(^_^)(^_^)(^_^)(^_^)(^_^)(^_^)(^_^)
(^_^)(^_^)(^_^)(^_^)(^_^)(^_^)(^_^)(^_^)(^_^)(^_^)(^_^)
(^_^)(^_^)(^_^)(^_^)(^_^)(^_^)(^_^)(^_^)(^_^)(^_^)(^_^)(^_^)(^_^)
(^_^)(^_^)(^_^)(^_^)(^_^)(^_^)(^_^)(^_^)(^_^)(^_^)(^_^)(^_^)(^_^)(^_^)(^_^)
(^_^)(^_^)(^_^)(^_^)(^_^)(^_^)(^_^)(^_^)(^_^)(^_^)(^_^)(^_^)(^_^)(^_^)(^_^)(^_^)(^_^)
(^_^)(^_^)(^_^)(^_^)(^_^)(^_^)(^_^)(^_^)(^_^)(^_^)(^_^)(^_^)(^_^)(^_^)(^_^)(^_^)(^_^)(^_^)(^_^)
(^_^)(^_^)(^_^)(^_^)(^_^)(^_^)(^_^)(^_^)(^_^)(^_^)(^_^)(^_^)(^_^)(^_^)(^_^)(^_^)(^_^)(^_^)(^_^)(^_^)(^_^)
(^_^)(^_^)(^_^)(^_^)(^_^)(^_^)(^_^)(^_^)(^_^)(^_^)(^_^)(^_^)(^_^)(^_^)(^_^)(^_^)(^_^)(^_^)(^_^)(^_^)(^_^)
(^_^)(^_^)
(^_^)(^_^)(^_^)(^_^)(^_^)(^_^)(^_^)(^_^)(^_^)(^_^)(^_^)(^_^)(^_^)(^_^)(^_^)(^_^)(^_^)(^_^)(^_^)(^_^)(^_^)
(^_^)(^_^)(^_^)(^_^)
(^_^)(^_^)(^_^)(^_^)(^_^)(^_^)(^_^)(^_^)(^_^)(^_^)(^_^)(^_^)(^_^)(^_^)(^_^)(^_^)(^_^)(^_^)(^_^)(^_^)(^_^)
(^_^)(^_^)(^_^)(^_^)(^_^)(^_^)
(^_^)(^_^)(^_^)(^_^)(^_^)(^_^)(^_^)(^_^)(^_^)(^_^)(^_^)(^_^)(^_^)(^_^)(^_^)(^_^)(^_^)(^_^)(^_^)(^_^)(^_^)
(^_^)(^_^)(^_^)(^_^)(^_^)(^_^)(^_^)(^_^)
(^_^)(^_^)(^_^)(^_^)(^_^)(^_^)(^_^)(^_^)(^_^)(^_^)(^_^)(^_^)(^_^)(^_^)(^_^)(^_^)(^_^)(^_^)(^_^)(^_^)(^_^)
(^_^)(^_^)(^_^)(^_^)(^_^)(^_^)(^_^)(^_^)(^_^)(^_^)



Current path is /Users/lsq/Desktop/PeerJ-Review/example_input_files.


There are 6 sequences to be joined.
001F_sequence_to_be_merged.seq
002F_sequence_to_be_merged.seq
003F_sequence_to_be_merged.seq
004R_sequence_to_be_merged.seq
005R_sequence_to_be_merged.seq
006R_sequence_to_be_merged.seq
needle -outfile=001F002F.needle -asequence=001F.seq -bsequence=002F.seq -gapopen=10 -gapextend=0.5
Needleman-Wunsch global alignment of two sequences

########################################
# Program: needle
# Rundate: Thu 28 Jan 2021 08:06:05
# Commandline: needle
#    -outfile 001F002F.needle
#    -asequence 001F.seq
#    -bsequence 002F.seq
#    -gapopen 10
#    -gapextend 0.5
# Align_format: srspair
# Report_file: 001F002F.needle
########################################

#=======================================
#
# Aligned_sequences: 2
# 1:
# 2:
# Matrix: EDNAFULL
# Gap_penalty: 10.0
# Extend_penalty: 0.5
#
# Length: 1764
# Identity:     388/1764 (22.0%)
# Similarity:   388/1764 (22.0%)
# Gaps:        1373/1764 (77.8%)
# Score: 1714.0
#
#
#=======================================

                  1 CTGTATTTCGATCAGTTCGGGGCCACTTCCTGATCGAGGGCGACCTGAAC     50

                  0 --------------------------------------------------      0

                 51 CCCGACAACAGCGACGTGGACAAGCTGTTCATCCAGCTGGTGCAGACCTA    100

                  0 --------------------------------------------------      0

                101 CAACCAGCTGTTCGAGGAAAACCCCATCAACGCCAGCGGCGTGGACGCCA    150
```

```
   0 --------------------------------------------------      0

 151 AGGCCATCCTGTCTGCCAGACTGAGCAAGAGCAGACGGCTGGAAAATCTG    200

   0 --------------------------------------------------      0

 201 ATCGCCCAGCTGCCCGGCGAGAAGAAGAATGGCCTGTTCGGAAACCTGAT    250

   0 --------------------------------------------------      0

 251 TGCCCTGAGCCTGGGCCTGACCCCCAACTTCAAGAGCAACTTCGACCTGG    300

   0 --------------------------------------------------      0

 301 CCGAGGATGCCAAACTGCAGCTGAGCAAGGACACCTACGACGACGACCTG    350

   0 --------------------------------------------------      0

 351 GACAACCTGCTGGCCCAGATCGGCGACCAGTACGCCGACCTGTTTCTGGC    400

   0 --------------------------------------------------      0

 401 CGCCAAGAACCTGTCCGACGCCATCCTGCTGAGCGACATCCTGAGAGTGA    450

   0 --------------------------------------------------      0

 451 ACACCGAGATCACCAAGGCCCCCCTGAGCGCCTCTATGATCAAGAGATAC    500

   0 --------------------------------------------------      0

 501 GACGAGCACCACCAGGACCTGACCCTGCTGAAAGCTCTCGTGCGGCAGCA    550

   0 --------------------------------------------------      0

 551 GCTGCCTGAGAAGTACAAAGAGATTTTCTTCGACCAGAGCAAGAACGGCT    600

   0 --------------------------------------------------      0

 601 ACGCCGGCTACATTGACGGCGGAGCCAGCCAGGAAGAGTTCTACAAGTTC    650
                                    ||             |.||
   1 -------------------------------GG-------------GATC      6

 651 ATCAAGCCCATCCTGGAAAAGATGGACGGCACCGAGGAACTGCTCGTGAA    700
     ||          ||||||   .||||||||||||||||||||||||||||
   7 AT----------CTGGAA---GTGGACGGCACCGAGGAACTGCTCGTGAA     43

 701 GCTGAACAGAGAGGACCTGCTGCGGAAGCAGCGGACCTTCGACAACGGCA    750
     ||||||||||||||||||||||||||||||||||||||||||||||||||
  44 GCTGAACAGAGAGGACCTGCTGCGGAAGCAGCGGACCTTCGACAACGGCA     93

 751 GCATCCCCCACCAGATCCACCTGGGAGAGCTGCACGCCATTCTGCGGCGG    800
     ||||||||||||||||||||||||||||||||||||||||||||||||||
  94 GCATCCCCCACCAGATCCACCTGGGAGAGCTGCACGCCATTCTGCGGCGG    143

 801 CAGGAAGATTTTTACCCATTCCTGAAGGACAACCGGGAAAAGATCGAGAA    850
     ||||||||||||||||||||||||||||||||||||||||||||||||||
 144 CAGGAAGATTTTTACCCATTCCTGAAGGACAACCGGGAAAAGATCGAGAA    193

 851 GATCCTGACCTTCCGCATCCCCTACTACGTGGGCCCTCTGGGCCAGGGGA    900
     ||||||||||||||||||||||||||||||||||||||| ||||||||||
 194 GATCCTGACCTTCCGCATCCCCTACTACGTGGGCCCTCT-GGCCAGGGGA    242

 901 AACAGCAGATTCGCCTGGATGACCAG-AAGAGCGAGGAAACCATCACCCC    949
     |||||||||||||||||||||||||| |||||||||||||||||||||||
 243 AACAGCAGATTCGCCTGGATGACCAGAAAGAGCGAGGAAACCATCACCCC    292

 950 CT-GCACTTCGAGGAAGT-GTGGACA--GGCGCT--CG-CCAGAGC-TCA    991
     || |.||||||||||||| |||||||  ||||||  || ||||||| |||
 293 CTGGAACTTCGAGGAAGTGGTGGACAAGGGCGCTTCCGCCCAGAGCTTCA    342

 992 TCGAGC-GATGAC--AC-TCGAT-AGA--CTG-CCAACGAG-AGGTGCTG   1032
     |||||| ||||||  || ||||| |||  ||| |||||||| ||||||||
 343 TCGAGCGGATGACCAACTTCGATAAGAACCTGCCCAACGAGAAGGTGCTG    392

1033 CC--AGCACAG-CTGCTGTA------------------------------   1049
     ||  ||||||| ||||||||
 393 CCCAAGCACAGCCTGCTGTACGAGTACTTCACCGTGTATAACGAGCTGAC    442

1049 --------------------------------------------------   1049

 443 CAAAGTGAAATACGTGACCGAGGGAATGAGAAAGCCCGCCTTCCTGAGCG    492
```

```
1049 --------------------------------------------------   1049

 493 GCGAGCAGAAAAAGGCCATCGTGGACCTGCTGTTCAAGACCAACCGGAAA    542

1049 --------------------------------------------------   1049

 543 GTGACCGTGAAGCAGCTGAAAGAGGACTACTTCAAGAAAATCGAGTGCTT    592

1049 --------------------------------------------------   1049

 593 CGACTCCGTGGAAATCTCCGGCGTGGAAGATCGGTTCAACGCCTCCCTGG    642

1049 --------------------------------------------------   1049

 643 GCACATACCACGATCTGCTGAAAATTATCAAGGACAAGGACTTCCTGGAC    692

1049 --------------------------------------------------   1049

 693 AATGAGGAAAACGAGGACATTCTGGAAGATATCGTGCTGACCCTGACACT    742

1049 --------------------------------------------------   1049

 743 GTTTGAGGACAGAGAGATGATCGAGGAACGGCTGAAAACCTATGCCCACC    792

1049 --------------------------------------------------   1049

 793 TGTTCGACGACAAAGTGATGAAGCAGCTGAAGCGGCGGAGATACACCGGC    842

1049 --------------------------------------------------   1049

 843 TGGGGCAGGCTGAGCCGGAAGCTGATCAACGGCATCCGGGACAAGCAGTC    892

1049 --------------------------------------------------   1049

 893 CGGGCAGACATCCTGGATTTCCTGAAGTCCGACGGCTTCGCAACAGAAAC    942

1049 --------------------------------------------------   1049

 943 TCATGCAGCTGATCACGACGACAGCTGACTTAAGAGACATCAGATGCCAG    992

1049 --------------------------------------------------   1049

 993 TGTCGCAGGCGATAGCTGCACGAGCAATGGCATCTGTGCAGGCCGCATAG   1042

1049 --------------------------------------------------   1049

1043 AGCATCTGCGACTGAGTGTGACAGCTCTGAATGATGACCGCCAAGCCGAA   1092

1049 --------------   1049

1093 AACTCTGGTATTCG   1106


#---------------------------------------
#---------------------------------------
The beginning of excellent alignment is shown below.

701  GCTGAACAGAGAGGACCTGCTGCGGAAGCAGCGGACCTTCGACAACGGCA   750
44   GCTGAACAGAGAGGACCTGCTGCGGAAGCAGCGGACCTTCGACAACGGCA    93


needle -outfile=002F003F.needle -asequence=002F.seq -bsequence=003F.seq -gapopen=10 -gapextend=0.5
Needleman-Wunsch global alignment of two sequences

########################################
# Program: needle
# Rundate: Thu 28 Jan 2021 08:06:05
# Commandline: needle
#    -outfile 002F003F.needle
#    -asequence 002F.seq
#    -bsequence 003F.seq
#    -gapopen 10
#    -gapextend 0.5
# Align_format: srspair
# Report_file: 002F003F.needle
########################################

#=======================================
#
# Aligned_sequences: 2
```

```
# 1:
# 2:
# Matrix: EDNAFULL
# Gap_penalty: 10.0
# Extend_penalty: 0.5
#
# Length: 1789
# Identity:     441/1789 (24.7%)
# Similarity:   441/1789 (24.7%)
# Gaps:        1332/1789 (74.5%)
# Score: 1787.5
#
#
#=======================================

                  1 GGGATCATCTGGAAGTGGACGGCACCGAGGAACTGCTCGTGAAGCTGAAC     50

                  0 --------------------------------------------------      0

                 51 AGAGAGGACCTGCTGCGGAAGCAGCGGACCTTCGACAACGGCAGCATCCC    100

                  0 --------------------------------------------------      0

                101 CCACCAGATCCACCTGGGAGAGCTGCACGCCATTCTGCGGCGGCAGGAAG    150

                  0 --------------------------------------------------      0

                151 ATTTTTACCCATTCCTGAAGGACAACCGGGAAAAGATCGAGAAGATCCTG    200

                  0 --------------------------------------------------      0

                201 ACCTTCCGCATCCCCTACTACGTGGGCCCTCTGGCCAGGGGAAACAGCAG    250

                  0 --------------------------------------------------      0

                251 ATTCGCCTGGATGACCAGAAAGAGCGAGGAAACCATCACCCCCTGGAACT    300

                  0 --------------------------------------------------      0

                301 TCGAGGAAGTGGTGGACAAGGGCGCTTCCGCCCAGAGCTTCATCGAGCGG    350

                  0 --------------------------------------------------      0

                351 ATGACCAACTTCGATAAGAACCTGCCCAACGAGAAGGTGCTGCCCAAGCA    400

                  0 --------------------------------------------------      0

                401 CAGCCTGCTGTACGAGTACTTCACCGTGTATAACGAGCTGACCAAAGTGA    450

                  0 --------------------------------------------------      0

                451 AATACGTGACCGAGGGAATGAGAAAGCCCGCCTTCCTGAGCGGCGAGCAG    500

                  0 --------------------------------------------------      0

                501 AAAAAGGCCATCGTGGACCTGCTGTTCAAGACCAACCGGAAAGTGACCGT    550

                  0 --------------------------------------------------      0

                551 GAAGCAGCTGAAAGAGGACTACTTCAAGAAAATCGAGTGCTTCGACTCCG    600

                  0 --------------------------------------------------      0

                601 TGGAAATCTCCGGCGTGGAAGATCGGTTCAACGCCTCCCTGGGCACA---    647
                                                             ||| ||
                  1 -----------------------------------------GGC-CAGGC      8

                648 TACCACGATCTGCTGAAAATTATCAAGGACAAGGACTTCCTGGACAATGA    697
                    || ||||||||||||  |||||||||||||||||||||||||||||||||
                  9 TA-CACGATCTGCTG--AATTATCAAGGACAAGGACTTCCTGGACAATGA     55

                698 GGAAAACGAGGACATTCTGGAAGATATCGTGCTGACCCTGACACTGTTTG    747
                    ||||||||||||||||||||||||||||||||||||||||||||||||||
                 56 GGAAAACGAGGACATTCTGGAAGATATCGTGCTGACCCTGACACTGTTTG    105

                748 AGGACAGAGAGATGATCGAGGAACGGCTGAAAACCTATGCCCACCTGTTC    797
                    ||||||||||||||||||||||||||||||||||||||||||||||||||
                106 AGGACAGAGAGATGATCGAGGAACGGCTGAAAACCTATGCCCACCTGTTC    155

                798 GACGACAAAGTGATGAAGCAGCTGAAGCGGCGGAGATACACCGGCTGGGG    847
                    ||||||||||||||||||||||||||||||||||||||||||||||||||
```

```
 156 GACGACAAAGTGATGAAGCAGCTGAAGCGGCGGAGATACACCGGCTGGGG    205

 848 CAGGCTGAGCCGGAAGCTGATCAACGGCATCCGGGACAAGCAGTCCGGGC    897
     ||||||||||||||||||||||||||||||||||||||||||||||||..
 206 CAGGCTGAGCCGGAAGCTGATCAACGGCATCCGGGACAAGCAGTCCGGCA    255

 898 AGAC-ATCCTGGATTTCCTGAAGTCCGACGGCTTCG-CAACAGAAAC-TC    944
     |||| ||||||||||||||||||||||||||||||| |||||||||| ||
 256 AGACAATCCTGGATTTCCTGAAGTCCGACGGCTTCGCCAACAGAAACTTC    305

 945 ATGCAGCTGAT-CACGACGACAG-CTGAC---TTAAGA-GACATC---AG    985
     ||||||||||| ||||||||||| |||||   |.|||| ||||||   |.
 306 ATGCAGCTGATCCACGACGACAGCCTGACCTTTAAAGAGGACATCCAGAA    355

 986 ATGCCA-GTGTC--GC--AGGCGATAG-CTGCACGAGCA-ATGGC--ATC   1026
     |..||| |||||  ||  .|||||||| ||||||||||| ||.||  |||
 356 AGCCCAGGTGTCCGGCCAGGGCGATAGCCTGCACGAGCACATTGCCAATC    405

1027 TGTGCAGGCCG------CAT-----AGAGCAT-CTGC-GAC--TGA----   1057
     || ||.|||.|      |||     ||.|||| |||| |||  |||
 406 TG-GCCGGCAGCCCCGCCATTAAGAAGGGCATCCTGCAGACAGTGAAGGT    454

1058 -GTGTGAC-AGCTC-TGAA--TGAT-GACCG--CCAAG-CCGAAAAC-TC   1097
      ||| ||| ||||| ||||  |||| |.|||  .|||| ||||.||| ||
 455 GGTG-GACGAGCTCGTGAAAGTGATGGGCCGGCACAAGCCCGAGAACATC    503

1098 TGGTATTCG-----------------------------------------   1106
       ||..|||
 504 --GTGATCGAAATGGCCAGAGAGAACCAGACCACCCAGAAGGGACAGAAG    551

1106 --------------------------------------------------   1106

 552 AACAGCCGCGAGAGAATGAAGCGGATCGAAGAGGGCATCAAAGAGCTGGG    601

1106 --------------------------------------------------   1106

 602 CAGCCAGATCCTGAAAGAACACCCCGTGGAAAACACCCAGCTGCAGAACG    651

1106 --------------------------------------------------   1106

 652 AGAAGCTGTACCTGTACTACCTGCAGAATGGGCGGGATATGTACGTGGAC    701

1106 --------------------------------------------------   1106

 702 CAGGAACTGGACATCAACCGGCTGTCCGACTACGATGTGGACCATATCGT    751

1106 --------------------------------------------------   1106

 752 GCCTCAGAGCTTTCTGAAGGACGACTCCATCGACAACAAAGTGCTGACCA    801

1106 --------------------------------------------------   1106

 802 GAAGCGACAAGAACCGGGGCAAGAGCGACAACGTGCCCTCCGAAGAGGTC    851

1106 --------------------------------------------------   1106

 852 GTGAAGAAGATGAAGAACTACTGGCGGCAGCTGCTGAACGCCCAAGCTGA    901

1106 --------------------------------------------------   1106

 902 TTACCCAGAGAAAGTTCGACATCTGACCAACGCCGAGAGAGGCGGCCTGA    951

1106 --------------------------------------------------   1106

 952 GCGAACTGGATAATGCGCTTCATCAGGAGACAGCTGATGAACCCCGCAGA   1001

1106 --------------------------------------------------   1106

1002 TCACAAAGCACGTGGCACAGATCTGACTCCCGGATGACACTTAGTACGAC   1051

1106 --------------------------------------------------   1106

1052 GAGATGACAGCCTGATCGGCAGTGAAGTGATCACCCTTGAGTTCAGCTTG   1101

1106 ---------------------------------------   1106

1102 GGTTCCGATTCGCAAAGAATTCCTAGCTTACCAAATTGC   1140


#---------------------------------------
#---------------------------------------
```

The beginning of excellent alignment is shown below.

```
698  GGAAAACGAGGACATTCTGGAAGATATCGTGCTGACCCTGACACTGTTTG   747
56   GGAAAACGAGGACATTCTGGAAGATATCGTGCTGACCCTGACACTGTTTG   105
```

```
needle -outfile=003F004R.needle -asequence=003F.seq -bsequence=004R.seq -gapopen=10 -gapextend=0.5
Needleman-Wunsch global alignment of two sequences

########################################
# Program: needle
# Rundate: Thu 28 Jan 2021 08:06:05
# Commandline: needle
#    -outfile 003F004R.needle
#    -asequence 003F.seq
#    -bsequence 004R.seq
#    -gapopen 10
#    -gapextend 0.5
# Align_format: srspair
# Report_file: 003F004R.needle
########################################

#=======================================
#
# Aligned_sequences: 2
# 1:
# 2:
# Matrix: EDNAFULL
# Gap_penalty: 10.0
# Extend_penalty: 0.5
#
# Length: 1181
# Identity:    1081/1181 (91.5%)
# Similarity:  1081/1181 (91.5%)
# Gaps:          70/1181 ( 5.9%)
# Score: 5021.5
#
#
#=======================================

   1 -----------------------GGCCAGGCTACACGA-TCTGCTGAA-T     25
                            ||.||..|..||||| ||||||||| .
   1 GATGGTCTGATTCAACGCCTCCTGGGCACACATCACGATTCTGCTGAAGC     50

  26 TATCAAGGACAAGGACTTCCTGGACAATGAGGAAAACGAGGACATTCTGG     75
     .||| ||||| |.||||.|.||..|||||| |||||||| |||| ||||.
  51 AATC-AGGAC-ATGACTGCTTGACCAATGA-GAAAACGA-GACA-TCTGA     95

  76 AAGATATCGTGCTGACCCTGACACTGTTTGAGGACAGAGAGATGATCGAG    125
     ||||||||||||||| ||||||||||||||||||||||||||||||||| 
  96 AAGATATCGTGCTGA-CCTGACACTGTTTGAGGACAGAGAGATGATCGA-    143

 126 GAACGGCTGAAAACCTATG-CCCACCTGTTCGACGACAAAGTGATGAAGC    174
     ||||||||||||||||||| ||||||||||||||||||||||||||||||
 144 GAACGGCTGAAAACCTATGCCCCACCTGTTCGACGACAAAGTGATGAAGC    193

 175 AGCTGAAGCGGCGGAGATACACCGGCTGGGGCAGGCTGAGCCGGAAGCTG    224
     ||||||||||||||||||||||||||||||||||||||||||||||||||
 194 AGCTGAAGCGGCGGAGATACACCGGCTGGGGCAGGCTGAGCCGGAAGCTG    243

 225 ATCAACGGCATCCGGGACAAGCAGTCCGGCAAGACAATCCTGGATTTCCT    274
     ||||||||||||||||||||||||||||||||||||||||||||||| ||
 244 ATCAACGGCATCCGGGACAAGCAGTCCGGCAAGACAATCCTGGATTT-CT    292

 275 GAAGTCCGACGGCTTCGCCAACAGAAACTTCATGCAGCTGATCCACGACG    324
     ||||||||||||||||||||||||||||||||||||||||||||||||||
 293 GAAGTCCGACGGCTTCGCCAACAGAAACTTCATGCAGCTGATCCACGACG    342

 325 ACAGCCTGACCTTTAAAGAGGACATCCAGAAAG-CCCAGGTGTCCGGCCA    373
     |||||.||||||||||||||||||||||||||| ||||||||||||||||
 343 ACAGCGTGACCTTTAAAGAGGACATCCAGAAAGCCCCAGGTGTCCGGCCA    392

 374 GGGCGATAGCCTGCACGAGCACATTGCCAATCTGGCCGGCAGCCCCGCCA    423
     ||||||||||||||||||||||||||||||||||||||||||||||||||
 393 GGGCGATAGCCTGCACGAGCACATTGCCAATCTGGCCGGCAGCCCCGCCA    442

 424 TTAAGAAGGGCATCCTGCAGACAGTGAAGGTGGTGGACGAGCTCGTGAAA    473
     ||||||||||||||||||||||||||||||||||||||||||||||||||
 443 TTAAGAAGGGCATCCTGCAGACAGTGAAGGTGGTGGACGAGCTCGTGAAA    492

 474 GTGATGGGCCGGCACAAGCCCGAGAACATCGTGATCGAAATGGCCAGAGA    523
```

```
                    ||||||||||||||||||||||||||||||||||||||||||||||||||
     493 GTGATGGGCCGGCACAAGCCCGAGAACATCGTGATCGAAATGGCCAGAGA    542

     524 GAACCAGACCACCCAGAAGGGACAGAAGAACAGCCGCGAGAGAATGAAGC    573
         ||||||||||||||||||||||||||||||||||||||||||||||||||
     543 GAACCAGACCACCCAGAAGGGACAGAAGAACAGCCGCGAGAGAATGAAGC    592

     574 GGATCGAAGAGGGCATCAAAGAGCTGGGCAGCCAGATCCTGAAAGAACAC    623
         ||||||||||||||||||||||||||||||||||||||||||||||||||
     593 GGATCGAAGAGGGCATCAAAGAGCTGGGCAGCCAGATCCTGAAAGAACAC    642

     624 CCCGTGGAAAACACCCAGCTGCAGAACGAGAAGCTGTACCTGTACTACCT    673
         ||||||||||||||||||||||||||||||||||||||||||||||||||
     643 CCCGTGGAAAACACCCAGCTGCAGAACGAGAAGCTGTACCTGTACTACCT    692

     674 GCAGAATGGGCGGGATATGTACGTGGACCAGGAACTGGACATCAACCGGC    723
         ||||||||||||||||||||||||||||||||||||||||||||||||||
     693 GCAGAATGGGCGGGATATGTACGTGGACCAGGAACTGGACATCAACCGGC    742

     724 TGTCCGACTACGATGTGGACCATATCGTGCCTCAGAGCTTTCTGAAGGAC    773
         ||||||||||||||||||||||||||||||||||||||||||||||||||
     743 TGTCCGACTACGATGTGGACCATATCGTGCCTCAGAGCTTTCTGAAGGAC    792

     774 GACTCCATCGACAACAAAGTGCTGACCAGAAGCGACAAGAACCGGGGCAA    823
         ||||||||||||||||.|||||||||||||||||||||||||||||||||
     793 GACTCCATCGACAACAAGGTGCTGACCAGAAGCGACAAGAACCGGGGCAA    842

     824 GAGCGACAACGTGCCCTCCGAAGAGGTCGTGAAGAAGATGAAGAACTACT    873
         ||||||||||||||||||||||||||||||||||||||||||||||||||
     843 GAGCGACAACGTGCCCTCCGAAGAGGTCGTGAAGAAGATGAAGAACTACT    892

     874 GGCGGCAGCTGCTGAACGCCCAAGCTGATTACCCAGAGAAAGTTCGAC-A    922
         |||||||||||||||||| ||||||||||||||||||||||||||||| |
     893 GGCGGCAGCTGCTGAACG-CCAAGCTGATTACCCAGAGAAAGTTCGACAA    941

     923 TCTGACCAACGCCGAGAGAGGCGGCCTGAGCGAACTGGATAATGC--GCT    970
         |||||||||.||||||||||||||||||||||||||||||||.||  |||
     942 TCTGACCAAGGCCGAGAGAGGCGGCCTGAGCGAACTGGATAAGGCCGGCT    991

     971 TCATCAGGAGACAGCTGATG--AACCCCGCAGATCACAAAGCACGTGGCA   1018
         ||||||.||||||||||.||  |||||.||||||||||||||||||||||
     992 TCATCAAGAGACAGCTGGTGGAAACCCGGCAGATCACAAAGCACGTGGCA   1041

    1019 CAGATC--TGACTCCCGGATG-ACACTTAGTACGACGAG-ATGACAGCCT   1064
         ||||||  .|||||||||||| |||||.||||||||||| ||||||..||
    1042 CAGATCCTGGACTCCCGGATGAACACTAAGTACGACGAGAATGACAAGCT   1091

    1065 GATC--GGCAGTG-AAGTGATCACCCTTGAGTTC-AGCTTGGGT-TCCGA   1109
         ||||  ||.|||| |||||||||||||..|||.| ||||  ||| |||||
    1092 GATCCGGGAAGTGAAAGTGATCACCCTGAAGTCCAAGCT--GGTGTCCGA   1139

    1110 TTCGCAAAGAATTCCTAGCTTACCAAATTGC   1140
         |.||  |||||  ||.|
    1140 TCCG--AAGAA--CCGA---------------   1152


#---------------------------------------
#---------------------------------------
The beginning of excellent alignment is shown below.

175  AGCTGAAGCGGCGGAGATACACCGGCTGGGGCAGGCTGAGCCGGAAGCTG   224
194  AGCTGAAGCGGCGGAGATACACCGGCTGGGGCAGGCTGAGCCGGAAGCTG   243


needle -outfile=004R005R.needle -asequence=004R.seq -bsequence=005R.seq -gapopen=10 -gapextend=0.5
Needleman-Wunsch global alignment of two sequences

########################################
# Program: needle
# Rundate: Thu 28 Jan 2021 08:06:05
# Commandline: needle
#    -outfile 004R005R.needle
#    -asequence 004R.seq
#    -bsequence 005R.seq
#    -gapopen 10
#    -gapextend 0.5
# Align_format: srspair
# Report_file: 004R005R.needle
########################################

#=======================================
```

```
#
# Aligned_sequences: 2
# 1:
# 2:
# Matrix: EDNAFULL
# Gap_penalty: 10.0
# Extend_penalty: 0.5
#
# Length: 1814
# Identity:     360/1814 (19.8%)
# Similarity:   360/1814 (19.8%)
# Gaps:        1452/1814 (80.0%)
# Score: 1679.0
#
#
#=======================================

                   1 GATGGTCTGATTCAACGCCTCCTGGGCACACATCACGATTCTGCTGAAGC     50

                   0 --------------------------------------------------      0

                  51 AATCAGGACATGACTGCTTGACCAATGAGAAAACGAGACATCTGAAAGAT    100

                   0 --------------------------------------------------      0

                 101 ATCGTGCTGACCTGACACTGTTTGAGGACAGAGAGATGATCGAGAACGGC    150

                   0 --------------------------------------------------      0

                 151 TGAAAACCTATGCCCCACCTGTTCGACGACAAAGTGATGAAGCAGCTGAA    200

                   0 --------------------------------------------------      0

                 201 GCGGCGGAGATACACCGGCTGGGGCAGGCTGAGCCGGAAGCTGATCAACG    250

                   0 --------------------------------------------------      0

                 251 GCATCCGGGACAAGCAGTCCGGCAAGACAATCCTGGATTTCTGAAGTCCG    300

                   0 --------------------------------------------------      0

                 301 ACGGCTTCGCCAACAGAAACTTCATGCAGCTGATCCACGACGACAGCGTG    350

                   0 --------------------------------------------------      0

                 351 ACCTTTAAAGAGGACATCCAGAAAGCCCCAGGTGTCCGGCCAGGGCGATA    400

                   0 --------------------------------------------------      0

                 401 GCCTGCACGAGCACATTGCCAATCTGGCCGGCAGCCCCGCCATTAAGAAG    450

                   0 --------------------------------------------------      0

                 451 GGCATCCTGCAGACAGTGAAGGTGGTGGACGAGCTCGTGAAAGTGATGGG    500

                   0 --------------------------------------------------      0

                 501 CCGGCACAAGCCCGAGAACATCGTGATCGAAATGGCCAGAGAGAACCAGA    550

                   0 --------------------------------------------------      0

                 551 CCACCCAGAAGGGACAGAAGAACAGCCGCGAGAGAATGAAGCGGATCGAA    600

                   0 --------------------------------------------------      0

                 601 GAGGGCATCAAAGAGCTGGGCAGCCAGATCCTGAAAGAACACCCCGTGGA    650

                   0 --------------------------------------------------      0

                 651 AAACACCCAGCTGCAGAACGAGAAGCTGTACCTGTACTACCTGCAGAATG    700

                   0 --------------------------------------------------      0

                 701 GGCGGGATATGTACGTGGACCAGGAACTGGACATCAACCGGCTGTCCGAC    750

                   0 --------------------------------------------------      0

                 751 TACGATGTGGACCATATCGTGCCTCAGAGCTTTCTGAAGGACGACTCCAT    800
                                                 ||||||||||||||||||||||
                   1 ----------------------------GCTTTCTGAAGGACGACTCCAT     22
```

```
 801 CGACAACAAGGTGCTGACCAGAAGCGACAAGAACCGGGGCAAGAGCGACA    850
     |||| ||||||||||||||||||||||||  .|||||||| ||||||||
  23 CGAC-ACAAGGTGCTGACCAGAAGCGACA--GACCGGGGC-AGAGCGAC-     67

 851 ACGTGCCCTCCGAAGAGGTCGTGAAGAAGATGAAGAACTACTGGCGGCAG    900
     |||||||||||||||| |||||| ||||||||||||||||||||||||||
  68 ACGTGCCCTCCGAAGA-GTCGTG-AGAAGATGAAGAACTACTGGCGGCAG    115

 901 CTGCTGAACGCCAAGCTGATTACCCAGAGAAAGTTCGACAATCTGACCAA    950
     ||||||||||||||||||||||||||||||||||||||||||||||||||
 116 CTGCTGAACGCCAAGCTGATTACCCAGAGAAAGTTCGACAATCTGACCAA    165

 951 GG-CCGAGAGAGGCGGCCTGAGCGAACTGGATAAGG-CCGGCTTCATCAA    998
     || ||||||||||||| ||||||||||||||||||| |||||||||||||
 166 GGCCCGAGAGAGGCGG-CTGAGCGAACTGGATAAGGCCCGGCTTCATCAA    214

 999 GAGACAGCTGGTGGAAACCCGGCAGATCACAAAGCACGTGGCACAGATCC   1048
     ||||||||||||||||||||||||||||||||||||||||||||||||||
 215 GAGACAGCTGGTGGAAACCCGGCAGATCACAAAGCACGTGGCACAGATCC    264

1049 TGGACTCCCGGATGAACACTAAGTACGACGAGAATGACAAGCTGATCCGG   1098
     ||||||||||||||||||||||||||||||||||||||||||||||||||
 265 TGGACTCCCGGATGAACACTAAGTACGACGAGAATGACAAGCTGATCCGG    314

1099 GAAGTGAAAGTGATCACCCTGAAGTCCAAGCTGGTGTCCGAT---CCGAA   1145
     ||||||||||||||||||||||||||||||||||||||||||   |.|||
 315 GAAGTGAAAGTGATCACCCTGAAGTCCAAGCTGGTGTCCGATTTCCGGAA    364

1146 GAACCGA-------------------------------------------   1152
     |    ||
 365 G----GATTTCCAGTTTTACAAAGTGCGCGAGATCAACAACTACCACCAC    410

1152 --------------------------------------------------   1152

 411 GCCCACGACGCCTACCTGAACGCCGTCGTGGGAACCGCCCTGATCAAAAA    460

1152 --------------------------------------------------   1152

 461 GTACCCTAAGCTGGAAAGCGAGTTCGTGTACGGCGACTACAAGGTGTACG    510

1152 --------------------------------------------------   1152

 511 ACGTGCGGAAGATGATCGCCAAGAGCGAGCAGGAAATCGGCAAGGCTACC    560

1152 --------------------------------------------------   1152

 561 GCCAAGTACTTCTTCTACAGCAACATCATGAACTTTTTCAAGACCGAGAT    610

1152 --------------------------------------------------   1152

 611 TACCCTGGCCAACGGCGAGATCCGGAAGCGGCCTCTGATCGAGACAAACG    660

1152 --------------------------------------------------   1152

 661 GCGAAACCGGGGAGATCGTGTGGGATAAGGGCCGGGATTTTGCCACCGTG    710

1152 --------------------------------------------------   1152

 711 CGGAAAGTGCTGAGCATGCCCCAAGTGAATATCGTGAAAAAGACCGAGGT    760

1152 --------------------------------------------------   1152

 761 GCAGACAGGCGGCTTCAGCAAAGAGTCTATCCTGCCCAAGAGGAACAGCG    810

1152 --------------------------------------------------   1152

 811 ATAAGCTGATCGCCAGAAAGAAGGACTGGGACCCTAAGAAGTACGGCGGC    860

1152 --------------------------------------------------   1152

 861 TTCGACAGCCCCACCGTGGCCTATTCTGTGCTGGTGGTGGCCAAAGTGGA    910

1152 --------------------------------------------------   1152

 911 AAAGGGCAAGTCCAAGAAACTGAAGAGTGTGAAAGAGCTGCTGGGGATCA    960

1152 --------------------------------------------------   1152

 961 CCATCATGGAAAGAAGCAGCTTCGAGAAGAATCCCATCGACTTCTGAAGC   1010

1152 --------------   1152
```

```
                1011 CAAGCACAGTGAGG   1024


#---------------------------------------
#---------------------------------------
The beginning of excellent alignment is shown below.

901  CTGCTGAACGCCAAGCTGATTACCCAGAGAAAGTTCGACAATCTGACCAA   950
116  CTGCTGAACGCCAAGCTGATTACCCAGAGAAAGTTCGACAATCTGACCAA   165


needle -outfile=005R006R.needle -asequence=005R.seq -bsequence=006R.seq -gapopen=10 -gapextend=0.5
Needleman-Wunsch global alignment of two sequences

########################################
# Program: needle
# Rundate: Thu 28 Jan 2021 08:06:05
# Commandline: needle
#    -outfile 005R006R.needle
#    -asequence 005R.seq
#    -bsequence 006R.seq
#    -gapopen 10
#    -gapextend 0.5
# Align_format: srspair
# Report_file: 005R006R.needle
########################################

#=======================================
#
# Aligned_sequences: 2
# 1:
# 2:
# Matrix: EDNAFULL
# Gap_penalty: 10.0
# Extend_penalty: 0.5
#
# Length: 1676
# Identity:     528/1676 (31.5%)
# Similarity:   528/1676 (31.5%)
# Gaps:        1129/1676 (67.4%)
# Score: 2368.5
#
#
#=======================================

                   1 GCTTTCTGAAGGACGACTCCATCGACACAAGGTGCTGACCAGAAGCGACA     50

                   0 --------------------------------------------------      0

                  51 GACCGGGGCAGAGCGACACGTGCCCTCCGAAGAGTCGTGAGAAGATGAAG    100

                   0 --------------------------------------------------      0

                 101 AACTACTGGCGGCAGCTGCTGAACGCCAAGCTGATTACCCAGAGAAAGTT    150

                   0 --------------------------------------------------      0

                 151 CGACAATCTGACCAAGGCCCGAGAGAGGCGGCTGAGCGAACTGGATAAGG    200

                   0 --------------------------------------------------      0

                 201 CCCGGCTTCATCAAGAGACAGCTGGTGGAAACCCGGCAGATCACAAAGCA    250

                   0 --------------------------------------------------      0

                 251 CGTGGCACAGATCCTGGACTCCCGGATGAACACTAAGTACGACGAGAATG    300

                   0 --------------------------------------------------      0

                 301 ACAAGCTGATCCGGGAAGTGAAAGTGATCACCCTGAAGTCCAAGCTGGTG    350

                   0 --------------------------------------------------      0

                 351 TCCGATTTCCGGAAGGATTTCCAGTTTTACAAAGTGCGCGAGATCAACAA    400

                   0 --------------------------------------------------      0

                 401 CTACCACCACGCCCACGACGCCTACCTGAACGCCGTCGTGGGAACCGCCC    450

                   0 --------------------------------------------------      0
```

```
 451 TGATCAAAAAGTACCCTAAGCTGGAAAGCGA--GTTCGTG-TACGGC-GA    496
                        ||| |||.||||  .||||.| ||||.| ||
   1 -------------------GCT-GAAGGCGAAGTTTCGAGTTACGACGGA     30

 497 CTACAAGGTGTACGACGTGCGGAAGATGATCGCCAAGAGCG-AGCAGGAA    545
     |||||| |.|||||||||.| |||||||||||.|||||||| |||..|||
  31 CTACAA-GAGTACGACGTAC-GAAGATGATCGGCAAGAGCGAAGCCAGAA     78

 546 ATCGGCAAGGCTACCGCCAAGTACTTCTTCTACAG-CAACATCATGAACT    594
     |||.||.|.|||| ||||||||||||||||||||| ||.|||||||||||
  79 ATCCGCCAAGCTA-CGCCAAGTACTTCTTCTACAGCCACCATCATGAACT    127

 595 TTTTCAAGACCGAGATTACCCTGGCCAACG-GCGAGATCC-GGAAGCGGC    642
     ||||||||| |||||||||||| ||||||| ||||||||| |.|||||||
 128 TTTTCAAGA-CGAGATTACCCT-GCCAACGAGCGAGATCCAGAAAGCGGC    175

 643 CTCTGATCGAGACAAACGGCGAAACCGGGGAGATCGTGTGGGATAAGGGC    692
     ||||||||||||| |.||||||||||.||||||||||||||||||||.||
 176 CTCTGATCGAGAC-ATCGGCGAAACCCGGGAGATCGTGTGGGATAAGAGC    224

 693 CGGGATTTTGCCACCGTGCGGAAAGTGCTGAGCATGCCCCAAGTGAATAT    742
     |||||||||| |||||||||||||||||||||||||||||||||||||||
 225 CGGGATTTTG-CACCGTGCGGAAAGTGCTGAGCATGCCCCAAGTGAATAT    273

 743 CGTGAAAAAGACCGAGGTGCAGACAGGCGGCTTCAGCAAAGAGTCTATCC    792
     ||||||||||||||||||||||||||||||||||||||||||||||||||
 274 CGTGAAAAAGACCGAGGTGCAGACAGGCGGCTTCAGCAAAGAGTCTATCC    323

 793 TGCCCAAGAGGAACAGCGATAAGCTGATCGCCAGAAAGAAGGACTGGGAC    842
     ||||||||||||||||||||||||||||||||||||||||||||||||||
 324 TGCCCAAGAGGAACAGCGATAAGCTGATCGCCAGAAAGAAGGACTGGGAC    373

 843 CCTAAGAAGTACGGCGGCTTCGACAGCCCCACCGTGGCCTATTCTGTGCT    892
     ||||||||||||||||||||||||||||||||||||||||||||||||||
 374 CCTAAGAAGTACGGCGGCTTCGACAGCCCCACCGTGGCCTATTCTGTGCT    423

 893 GGTGGTGGCCAAAGTGGAAAAGGGCAAGTCCAAGAAACTGAAGAGTGTGA    942
     ||||||||||||||||||||||||||||||||||||||||||||||||||
 424 GGTGGTGGCCAAAGTGGAAAAGGGCAAGTCCAAGAAACTGAAGAGTGTGA    473

 943 AAGAGCTGCTGGGGATCACCATCATGGAAAGAAGCAGCTTCGAGAAGAAT    992
     ||||||||||||||||||||||||||||||||||||||||||||||||||
 474 AAGAGCTGCTGGGGATCACCATCATGGAAAGAAGCAGCTTCGAGAAGAAT    523

 993 CCCATCGAC-TTCT-GAAGCCAAGCAC--------AGTG----AGG----   1024
     ||||||||| |||| |||||||||..|        ||||    |||
 524 CCCATCGACTTTCTGGAAGCCAAGGGCTACAAAGAAGTGAAAAAGGACCT    573

1024 --------------------------------------------------   1024

 574 GATCATCAAGCTGCCTAAGTACTCCCTGTTCGAGCTGGAAAACGGCCGGA    623

1024 --------------------------------------------------   1024

 624 AGAGAATGCTGGCCTCTGCCGGCGAACTGCAGAAGGGAAACGAACTGGCC    673

1024 --------------------------------------------------   1024

 674 CTGCCCTCCAAATATGTGAACTTCCTGTACCTGGCCAGCCACTATGAGAA    723

1024 --------------------------------------------------   1024

 724 GCTGAAGGGCTCCCCCGAGGATAATGAGCAGAAACAGCTGTTTGTGGAAC    773

1024 --------------------------------------------------   1024

 774 AGCACAAGCACTACCTGGACGAGATCATCGAGCAGATCAGCGAGTTCTCC    823

1024 --------------------------------------------------   1024

 824 AAGAGAGTGATCCTGGCCGACGCTAATCTGGACAAAGTGCTGTCCGCCTA    873

1024 --------------------------------------------------   1024

 874 CAACAAGCACCGGGATAAGCCCATCAGAGAGCAGGCCGAGAATATCATCC    923

1024 --------------------------------------------------   1024

 924 ACCTGTTTACCCTGACCAATCTGGGAGCCCCTGCCGCCTTCAAGTACTTT    973
```

```
1024 --------------------------------------------------   1024

 974 GACACCACCATCGACCGGAAGAGGTACACCAGCACCAAAGAGGTGCTGGA   1023

1024 --------------------------------------------------   1024

1024 CGCCACCCTGATCCACCAGAGCATCACCGGCCTGTACGAGACACGGATCG   1073

1024 --------------------------------------------------   1024

1074 ACCTGTCTCAGCTGGGAGGCGACCCAAAGAAGAAGCGGAAGGTCTGAAAG   1123

1024 --------------------------------------------------   1024

1124 CTTGCGGCCGCACTCGAGCACCACCACCACCACCACTGAGATCCGGCTGC   1173

1024 --------------------------   1024

1174 TAACAAAGCCCGAAAGAGCGATTTCC   1199
```

```
#---------------------------------------
#---------------------------------------
The beginning of excellent alignment is shown below.

743  CGTGAAAAAGACCGAGGTGCAGACAGGCGGCTTCAGCAAAGAGTCTATCC   792
274  CGTGAAAAAGACCGAGGTGCAGACAGGCGGCTTCAGCAAAGAGTCTATCC   323
```

The merged DNA sequence is shown below.

```
CTGTATTTCGATCAGTTCGGGGCCACTTCCTGATCGAGGGCGACCTGAACCCCGACAACAGCGACGTGGACAAGCTGTTCATCCAGCTGGTGCAGACCTA
CAACCAGCTGTTCGAGGAAAACCCCATCAACGCCAGCGGCGTGGACGCCAAGGCCATCCTGTCTGCCAGACTGAGCAAGAGCAGACGGCTGGAAAATCTG
ATCGCCCAGCTGCCCGGCGAGAAGAAGAATGGCCTGTTCGGAAACCTGATTGCCCTGAGCCTGGGCCTGACCCCCAACTTCAAGAGCAACTTCGACCTGG
CCGAGGATGCCAAACTGCAGCTGAGCAAGGACACCTACGACGACGACCTGGACAACCTGCTGGCCCAGATCGGCGACCAGTACGCCGACCTGTTTCTGGC
CGCCAAGAACCTGTCCGACGCCATCCTGCTGAGCGACATCCTGAGAGTGAACACCGAGATCACCAAGGCCCCCCTGAGCGCCTCTATGATCAAGAGATAC
GACGAGCACCACCAGGACCTGACCCTGCTGAAAGCTCTCGTGCGGCAGCAGCTGCCTGAGAAGTACAAAGAGATTTTCTTCGACCAGAGCAAGAACGGCT
ACGCCGGCTACATTGACGGCGGAGCCAGCCAGGAAGAGTTCTACAAGTTCATCAAGCCCATCCTGGAAAAGATGGACGGCACCGAGGAACTGCTCGTGAA
GCTGAACAGAGAGGACCTGCTGCGGAAGCAGCGGACCTTCGACAACGGCAGCATCCCCCACCAGATCCACCTGGGAGAGCTGCACGCCATTCTGCGGCGG
CAGGAAGATTTTTACCCATTCCTGAAGGACAACCGGGAAAAGATCGAGAAGATCCTGACCTTCCGCATCCCCTACTACGTGGGCCCTCTGGCCAGGGGAA
ACAGCAGATTCGCCTGGATGACCAGAAAGAGCGAGGAAACCATCACCCCCTGGAACTTCGAGGAAGTGGTGGACAAGGGCGCTTCCGCCCAGAGCTTCAT
CGAGCGGATGACCAACTTCGATAAGAACCTGCCCAACGAGAAGGTGCTGCCCAAGCACAGCCTGCTGTACGAGTACTTCACCGTGTATAACGAGCTGACC
AAAGTGAAATACGTGACCGAGGGAATGAGAAAGCCCGCCTTCCTGAGCGGCGAGCAGAAAAAGGCCATCGTGGACCTGCTGTTCAAGACCAACCGGAAAG
TGACCGTGAAGCAGCTGAAAGAGGACTACTTCAAGAAAATCGAGTGCTTCGACTCCGTGGAAATCTCCGGCGTGGAAGATCGGTTCAACGCCTCCCTGGG
CACATACCACGATCTGCTGAAAATTATCAAGGACAAGGACTTCCTGGACAATGAGGAAAACGAGGACATTCTGGAAGATATCGTGCTGACCCTGACACTG
TTTGAGGACAGAGAGATGATCGAGGAACGGCTGAAAACCTATGCCCACCTGTTCGACGACAAAGTGATGAAGCAGCTGAAGCGGCGGAGATACACCGGCT
GGGGCAGGCTGAGCCGGAAGCTGATCAACGGCATCCGGGACAAGCAGTCCGGCAAGACAATCCTGGATTTCTGAAGTCCGACGGCTTCGCCAACAGAAAC
TTCATGCAGCTGATCCACGACGACAGCGTGACCTTTAAAGAGGACATCCAGAAAGCCCCAGGTGTCCGGCCAGGGCGATAGCCTGCACGAGCACATTGCC
AATCTGGCCGGCAGCCCCGCCATTAAGAAGGGCATCCTGCAGACAGTGAAGGTGGTGGACGAGCTCGTGAAAGTGATGGGCCGGCACAAGCCCGAGAACA
TCGTGATCGAAATGGCCAGAGAGAACCAGACCACCCAGAAGGGACAGAAGAACAGCCGCGAGAGAATGAAGCGGATCGAAGAGGGCATCAAAGAGCTGGG
CAGCCAGATCCTGAAAGAACACCCCGTGGAAAACACCCAGCTGCAGAACGAGAAGCTGTACCTGTACTACCTGCAGAATGGGCGGGATATGTACGTGGAC
CAGGAACTGGACATCAACCGGCTGTCCGACTACGATGTGGACCATATCGTGCCTCAGAGCTTTCTGAAGGACGACTCCATCGACAACAAGGTGCTGACCA
GAAGCGACAAGAACCGGGGCAAGAGCGACAACGTGCCCTCCGAAGAGGTCGTGAAGAAGATGAAGAACTACTGGCGGCAGCTGCTGAACGCCAAGCTGAT
TACCCAGAGAAAGTTCGACAATCTGACCAAGGCCCGAGAGAGGCGGCTGAGCGAACTGGATAAGGCCCGGCTTCATCAAGAGACAGCTGGTGGAAACCCG
GCAGATCACAAAGCACGTGGCACAGATCCTGGACTCCCGGATGAACACTAAGTACGACGAGAATGACAAGCTGATCCGGGAAGTGAAAGTGATCACCCTG
AAGTCCAAGCTGGTGTCCGATTTCCGGAAGGATTTCCAGTTTTACAAAGTGCGCGAGATCAACAACTACCACCACGCCCACGACGCCTACCTGAACGCCG
TCGTGGGAACCGCCCTGATCAAAAAGTACCCTAAGCTGGAAAGCGAGTTCGTGTACGGCGACTACAAGGTGTACGACGTGCGGAAGATGATCGCCAAGAG
CGAGCAGGAAATCGGCAAGGCTACCGCCAAGTACTTCTTCTACAGCAACATCATGAACTTTTTCAAGACCGAGATTACCCTGGCCAACGGCGAGATCCGG
AAGCGGCCTCTGATCGAGACAAACGGCGAAACCGGGGAGATCGTGTGGGATAAGGGCCGGGATTTTGCCACCGTGCGGAAAGTGCTGAGCATGCCCCAAG
TGAATATCGTGAAAAAGACCGAGGTGCAGACAGGCGGCTTCAGCAAAGAGTCTATCCTGCCCAAGAGGAACAGCGATAAGCTGATCGCCAGAAAGAAGGA
CTGGGACCCTAAGAAGTACGGCGGCTTCGACAGCCCCACCGTGGCCTATTCTGTGCTGGTGGTGGCCAAAGTGGAAAAGGGCAAGTCCAAGAAACTGAAG
AGTGTGAAAGAGCTGCTGGGGATCACCATCATGGAAAGAAGCAGCTTCGAGAAGAATCCCATCGACTTTCTGGAAGCCAAGGGCTACAAAGAAGTGAAAA
AGGACCTGATCATCAAGCTGCCTAAGTACTCCCTGTTCGAGCTGGAAAACGGCCGGAAGAGAATGCTGGCCTCTGCCGGCGAACTGCAGAAGGGAAACGA
ACTGGCCCTGCCCTCCAAATATGTGAACTTCCTGTACCTGGCCAGCCACTATGAGAAGCTGAAGGGCTCCCCCGAGGATAATGAGCAGAAACAGCTGTTT
GTGGAACAGCACAAGCACTACCTGGACGAGATCATCGAGCAGATCAGCGAGTTCTCCAAGAGAGTGATCCTGGCCGACGCTAATCTGGACAAAGTGCTGT
CCGCCTACAACAAGCACCGGGATAAGCCCATCAGAGAGCAGGCCGAGAATATCATCCACCTGTTTACCCTGACCAATCTGGGAGCCCCTGCCGCCTTCAA
GTACTTTGACACCACCATCGACCGGAAGAGGTACACCAGCACCAAAGAGGTGCTGGACGCCACCCTGATCCACCAGAGCATCACCGGCCTGTACGAGACA
CGGATCGACCTGTCTCAGCTGGGAGGCGACCCAAAGAAGAAGCGGAAGGTCTGAAAGCTTGCGGCCGCACTCGAGCACCACCACCACCACCACTGAGATC
CGGCTGCTAACAAAGCCCGAAAGAGCGATTTCC
```
