# Supplementary material for: A Python script to merge Sanger sequences
Source: PeerJ. 2021 Apr 27;9:e11354. doi: 10.7717/peerj.11354 (PMC8086567; doi:10.7717/peerj.11354)
Supplement: Supplemental Information 11 [file peerj-09-11354-s011.pdf]

1. The original Sanger sequencing file in seq format is changed to fasta format with TextEdit. The forward Sanger sequencing files are stored as F000~003.fasta and the reverse Sanger sequencing files are stored as R004~006.fasta.

2. The files are uploaded to website at http://hvdr.bioinf.wits.ac.za/fmt/ for sequence merging and the following sequence is obtained.

```
>sequence
GCTGACTCTTCCCTCTAGAATAATTTTGTTTAACTTTAAGAAGGAGATATACCATGGGCAGCAGCCATCATCATCATCATCACAGCAGCGGCCTGG
AAGTTCTGTTCCAGGGGCCCCATATGGCTAGCATGACTGGTGGACAGCAAATGGGTCGCGGATCCCCAAAGAAGAAGCGGAAGGTCGGTATCCACG
GAGTCCCAGCAGCCGACAAGAAGTACAGCATCGGCCTGGACATCGGCACCAACTCTGTGGGCTGGGCCGTGATCACCGACGAGTACAAGGTGCCCA
GCAAGAAATTCAAGGTGCTGGGCAACACCGACCGGCACAGCATCAAGAAGAACCTGATCGGAGCCCTGCTGTTCGACAGCGGCGAAACAGCCGAGG
CCACCCGGCTGAAGAGAACCGCCAGAAGAAGATACACCAGACGGAAGAACCGGATCTGCTATCTGCAAGAGATCTTCAGCAACGAGATGGCCAAGG
TGGACGACAGCTTCTTCCACAGACTGGAAGAGTCCTTCCTGGTGGAAGAGGATAAGAAGCACGAGCGGCACCCCATCTTCGGCAACATCGTGGACG
AGGTGGCCTACCACGAGAAGTACCCCACCATCTACCACCTGAGAAAGAAACTGGTGGACAGCACCGACAAGGCCGACCTGCGGCTGATCTATCTGG
CCCTGGCCCACATGATCAAGTTCCGGGGCCACTTCCTGATCGAGGGCGACCTGAACCCCGACAACAGCGACGTGGACAAGCTGTTCATCCAGCTGG
TGCAGACCTACAACCAGCTGTTCGAGGAAAACCCCATCAACGCCAGCGGCGTGGACGCCAAGGCCATCCTGTCTGCCAGACTGAGCAAGAGCAGAC
GGCTGGAAAATCTGATCGCCCAGCTGCCCGGCGAGAAGAAGAATGGCCCTGTTCGGAAACCTGATTGCCCTGAGCCTGGGCCTGACCCCCAACTTC
AAGAGCAACTTCGACCTGGCCGAGGATGCCAAACTGCAGCTGAGCAAGGACACCTACGACGACGACCTGACACCTGCTGGCCCAGATCGGCGACCA
GTACGCCGACCTGTTTTCTATGCAGACTGTCGACGCATCTGCTGACGACATCTGAGAGTGAACACGAGATCACAGCTCCCTTGACCTTATGATCAG
AGAATATCGATCGAGCACCTATCGAACCTGACCCTGCTGAAAGCTCTCGTGCGGCAGCAGCTGCCTGAGAAGTACAAAGAGATTTTCTTCGACCAG
AGCAAGAACGGCTACGCCGGCTACATTGACGGCGGAGCCAGCCAGGAAGAGTTCTACAAGTTCATCAAGCCCATCCTGGAAAAGATGGACGGCACC
GAGGAACTGCTCGTGAAGCTGAACAGAGAGGACCTGCTGCGGAAGCAGCGGACCTTCGACAACGGCAGCATCCCCCACCAGATCCACCTGGGAGAG
CTGCACGCCATTCTGCGGCGGCAGGAAGATTTTTACCCATTCCTGAAGGACAACCGGGAAAAGATCGAGAAGATCCTGACCTTCCGCATCCCCTAC
TACGTGGGCCCTCTGGGCCAGGGGAAACAGCAGATTCGCCTGGATGACCAGAAGAGCGAGGAAACCATCACCCCCTGCACTTCGAGGAAGTGTGGA
CAGGCGCTCGCCAGAGCTCATCGAGCGATGACACTCGATAGACTGCCAACGAGAGGTGCCCAACGAGAAGGTGCTGCCCAAGCACAGCCTGCTGTA
CGAGTACTTCACCGTGTATAACGAGCTGACCAAAGTGAAATACGTGACCGAGGGAATGAGAAAGCCCGCCTTCCTGAGCGGCGAGCAGAAAAAGGC
CATCGTGGACCTGCTGTTCAAGACCAACCGGAAAGTGACCGTGAAGCAGCTGAAAGAGGACTACTTCAAGAAAATCGAGTGCTTCGACTCCGTGGA
AATCTCCGGCGTGGAAGATCGGTTCAACGCCTCCCTGGGCACATACCACGATCTGCTGAAAATTATCAAGGACAAGGACTTCCTGGACAATGAGGA
AAACGAGGACATTCTGGAAGATATCGTGCTGACCCTGACACTGTTTGAGGACAGAGAGATGATCGAGGAACGGCTGAAAACCTATGCCCACCTGTT
CGACGACAAAGTGATGAAGCAGCTGAAGCGGCGGAGATACACCGGCTGGGGCAGGCTGAGCCGGAAGCTGATCAACGGCATCCGGGACAAGCAGTC
CGGGCAGACATCCTGGATTTCCTGAAGTCCGACGGCTTCGCAACAGAAACTCATGCAGCTGATCACGACGACAGCTGACTTAAGAGACATCAGATG
CCAGTGTCGCAGGCGATAGCTGCACGAGCAATGGCATCTGTGCAGGCCGCATAGAGCATCTGCGACTGAGTGTGACAGCTCTGAATGATGACCGCC
AAGAAGGTGGTGGACGAGCTCGTGAAAGTGATGGGCCGGCACAAGCCCGAGAACATCGTGATCGAAATGGCCAGAGAGAACCAGACCACCCAGAAG
GGACAGAAGAACAGCCGCGAGAGAATGAAGCGGATCGAAGAGGGCATCAAAGAGCTGGGCAGCCAGATCCTGAAAGAACACCCCGTGGAAAACACC
CAGCTGCAGAACGAGAAGCTGTACCTGTACTACCTGCAGAATGGGCGGGATATGTACGTGGACCAGGAACTGGACATCAACCGGCTGTCCGACTAC
GATGTGGACCATATCGTGCCTCAGAGCTTTCTGAAGGACGACTCCATCGACAACAAAGTGCTGACCAGAAGCGACAAGAACCGGGGCAAGAGCGAC
AACGTGCCCTCCGAAGAGGTCGTGAAGAAGATGAAGAACTACTGGCGGCAGCTGCTGAACGCCCAAGCTGATTACCCAGAGAAAGTTCGACATCTG
ACCAACGCCGAGAGAGGCGGCCTGAGCGAACTGGATAATGCGCTTCATCAGGAGACAGCTGATGAACCCCGCAGATCACAAAGCACGTGGCACAGA
TCTGACTCCCGGATGACACTTAGTACGACGAGATGACAGCCTGATCGGCAGTGAAGTGATCACCCTTGAGTTCAGCTTGGGTTCCGATTCGCAAAG
AATTTCCAGTTTTACAAAGTGCGCGAGATCAACAACTACCACCACGCCCACGACGCCTACCTGAACGCCGTCGTGGGAACCGCCCTGATCAAAAAG
TACCCTAAGCTGGAAAGCGAGTTCGTGTACGGCGACTACAAGGTGTACGACGTGCGGAAGATGATCGCCAAGAGCGAGCAGGAAATCGGCAAGGCT
ACCGCCAAGTACTTCTTCTACAGCAACATCATGAACTTTTTCAAGACCGAGATTACCCTGGCCAACGGCGAGATCCGGAAGCGGCCTCTGATCGAG
ACAAACGGCGAAACCGGGGAGATCGTGTGGGATAAGGGCCGGGATTTTGCCACCGTGCGGAAAGTGCTGAGCATGCCCCAAGTGAATATCGTGAAA
AAGACCGAGGTGCAGACAGGCGGCTTCAGCAAAGAGTCTATCCTGCCCAAGAGGAACAGCGATAAGCTGATCGCCAGAAAGAAGGACTGGGACCCT
AAGAAGTACGGCGGCTTCGACAGCCCCACCGTGGCCTATTCTGTGCTGGTGGTGGCCAAAGTGGAAAAGGGCAAGTCCAAGAAACTGAAGAGTGTG
AAAGAGCTGCTGGGGATCACCATCATGGAAAGAAGCAGCTTCGAGAAGAATCCCATCGACTTCTGAAGCCAAGGGCTACAAAGAAGTGAAAAAGGA
CCTGATCATCAAGCTGCCTAAGTACTCCCTGTTCGAGCTGGAAAACGGCCGGAAGAGAATGCTGGCCTCTGCCGGCGAACTGCAGAAGGGAAACGA
ACTGGCCCTGCCCTCCAAATATGTGAACTTCCTGTACCTGGCCAGCCACTATGAGAAGCTGAAGGGCTCCCCCGAGGATAATGAGCAGAAACAGCT
GTTTGTGGAACAGCACAAGCACTACCTGGACGAGATCATCGAGCAGATCAGCGAGTTCTCCAAGAGAGTGATCCTGGCCGACGCTAATCTGGACAA
AGTGCTGTCCGCCTACAACAAGCACCGGGATAAGCCCATCAGAGAGCAGGCCGAGAATATCATCCACCTGTTTACCCTGACCAATCTGGGAGCCCC
TGCCGCCTTCAAGTACTTTGACACCACCATCGACCGGAAGAGGTACACCAGCACCAAAGAGGTGCTGGACGCCACCCTGATCCACCAGAGCATCAC
CGGCCTGTACGAGACACGGATCGACCTGTCTCAGCTGGGAGGCGACCCAAAGAAGAAGCGGAAGGTCTGAAAGCTTGCGGCCGCACTCGAGCACCA
CCACCACCACCACTGAGATCCGGCTGCTAACAAAGCCCGAAAGAGCGATTTCC
```

3. The manually merged sequence is shown as follows:

```
>gene
GCTGACTCTTCCCTCTAGAATAATTTTGTTTAACTTTAAGAAGGAGATATACCATGGGCAGCAGCCATCATCATCATCATCACAGCAGCGGCCTGG
AAGTTCTGTTCCAGGGGCCCCATATGGCTAGCATGACTGGTGGACAGCAAATGGGTCGCGGATCCCCAAAGAAGAAGCGGAAGGTCGGTATCCACG
GAGTCCCAGCAGCCGACAAGAAGTACAGCATCGGCCTGGACATCGGCACCAACTCTGTGGGCTGGGCCGTGATCACCGACGAGTACAAGGTGCCCA
GCAAGAAATTCAAGGTGCTGGGCAACACCGACCGGCACAGCATCAAGAAGAACCTGATCGGAGCCCTGCTGTTCGACAGCGGCGAAACAGCCGAGG
CCACCCGGCTGAAGAGAACCGCCAGAAGAAGATACACCAGACGGAAGAACCGGATCTGCTATCTGCAAGAGATCTTCAGCAACGAGATGGCCAAGG
TGGACGACAGCTTCTTCCACAGACTGGAAGAGTCCTTCCTGGTGGAAGAGGATAAGAAGCACGAGCGGCACCCCATCTTCGGCAACATCGTGGACG
AGGTGGCCTACCACGAGAAGTACCCCACCATCTACCACCTGAGAAAGAAACTGGTGGACAGCACCGACAAGGCCGACCTGCGGCTGATCTATCTGG
CCCTGGCCCACATGATCAAGTTCCGGGGCCACTTCCTGATCGAGGGCGACCTGAACCCCGACAACAGCGACGTGGACAAGCTGTTCATCCAGCTGG
TGCAGACCTACAACCAGCTGTTCGAGGAAAACCCCATCAACGCCAGCGGCGTGGACGCCAAGGCCATCCTGTCTGCCAGACTGAGCAAGAGCAGAC
GGCTGGAAAATCTGATCGCCCAGCTGCCCGGCGAGAAGAAGAATGGCCTGTTCGGAAACCTGATTGCCCTGAGCCTGGGCCTGACCCCCAACTTCA
AGAGCAACTTCGACCTGGCCGAGGATGCCAAACTGCAGCTGAGCAAGGACACCTACGACGACGACCTGGACAACCTGCTGGCCCAGATCGGCGACC
AGTACGCCGACCTGTTTCTGGCCGCCAAGAACCTGTCCGACGCCATCCTGCTGAGCGACATCCTGAGAGTGAACACCGAGATCACCAAGGCCCCCC
TGAGCGCCTCTATGATCAAGAGATACGACGAGCACCACCAGGACCTGACCCTGCTGAAAGCTCTCGTGCGGCAGCAGCTGCCTGAGAAGTACAAAG
AGATTTTCTTCGACCAGAGCAAGAACGGCTACGCCGGCTACATTGACGGCGGAGCCAGCCAGGAAGAGTTCTACAAGTTCATCAAGCCCATCCTGG
AAAAGATGGACGGCACCGAGGAACTGCTCGTGAAGCTGAACAGAGAGGACCTGCTGCGGAAGCAGCGGACCTTCGACAACGGCAGCATCCCCCACC
AGATCCACCTGGGAGAGCTGCACGCCATTCTGCGGCGGCAGGAAGATTTTTACCCATTCCTGAAGGACAACCGGGAAAAGATCGAGAAGATCCTGA
CCTTCCGCATCCCCTACTACGTGGGCCCTCTGGCCAGGGGAAACAGCAGATTCGCCTGGATGACCAGAAAGAGCGAGGAAACCATCACCCCCTGGA
ACTTCGAGGAAGTGGTGGACAAGGGCGCTTCCGCCCAGAGCTTCATCGAGCGGATGACCAACTTCGATAAGAACCTGCCCAACGAGAAGGTGCTGC
CCAAGCACAGCCTGCTGTACGAGTACTTCACCGTGTATAACGAGCTGACCAAAGTGAAATACGTGACCGAGGGAATGAGAAAGCCCGCCTTCCTGA
```

```
GCGGCGAGCAGAAAAAGGCCATCGTGGACCTGCTGTTCAAGACCAACCGGAAAGTGACCGTGAAGCAGCTGAAAGAGGACTACTTCAAGAAAATCG
AGTGCTTCGACTCCGTGGAAATCTCCGGCGTGGAAGATCGGTTCAACGCCTCCCTGGGCACATACCACGATCTGCTGAAAATTATCAAGGACAAGG
ACTTCCTGGACAATGAGGAAAACGAGGACATTCTGGAAGATATCGTGCTGACCCTGACACTGTTTGAGGACAGAGAGATGATCGAGGAACGGCTGA
AAACCTATGCCCACCTGTTCGACGACAAAGTGATGAAGCAGCTGAAGCGGCGGAGATACACCGGCTGGGGCAGGCTGAGCCGGAAGCTGATCAACG
GCATCCGGGACAAGCAGTCCGGCAAGACAATCCTGGATTTCTGAAGTCCGACGGCTTCGCCAACAGAAACTTCATGCAGCTGATCCACGACGACAG
CGTGACCTTTAAAGAGGACATCCAGAAAGCCCCAGGTGTCCGGCCAGGGCGATAGCCTGCACGAGCACATTGCCAATCTGGCCGGCAGCCCCGCCA
TTAAGAAGGGCATCCTGCAGACAGTGAAGGTGGTGGACGAGCTCGTGAAAGTGATGGGCCGGCACAAGCCCGAGAACATCGTGATCGAAATGGCCA
GAGAGAACCAGACCACCCAGAAGGGACAGAAGAACAGCCGCGAGAGAATGAAGCGGATCGAAGAGGGCATCAAAGAGCTGGGCAGCCAGATCCTGA
AAGAACACCCCGTGGAAAACACCCAGCTGCAGAACGAGAAGCTGTACCTGTACTACCTGCAGAATGGGCGGGATATGTACGTGGACCAGGAACTGG
ACATCAACCGGCTGTCCGACTACGATGTGGACCATATCGTGCCTCAGAGCTTTCTGAAGGACGACTCCATCGACAACAAGGTGCTGACCAGAAGCG
ACAAGAACCGGGGCAAGAGCGACAACGTGCCCTCCGAAGAGGTCGTGAAGAAGATGAAGAACTACTGGCGGCAGCTGCTGAACGCCAAGCTGATTA
CCCAGAGAAAGTTCGACAATCTGACCAAGGCCCGAGAGAGGCGGCTGAGCGAACTGGATAAGGCCCGGCTTCATCAAGAGACAGCTGGTGGAAACC
CGGCAGATCACAAAGCACGTGGCACAGATCCTGGACTCCCGGATGAACACTAAGTACGACGAGAATGACAAGCTGATCCGGGAAGTGAAAGTGATC
ACCCTGAAGTCCAAGCTGGTGTCCGATTTCCGGAAGGATTTCCAGTTTTACAAAGTGCGCGAGATCAACAACTACCACCACGCCCACGACGCCTAC
CTGAACGCCGTCGTGGGAACCGCCCTGATCAAAAAGTACCCTAAGCTGGAAAGCGAGTTCGTGTACGGCGACTACAAGGTGTACGACGTGCGGAAG
ATGATCGCCAAGAGCGAGCAGGAAATCGGCAAGGCTACCGCCAAGTACTTCTTCTACAGCAACATCATGAACTTTTTCAAGACCGAGATTACCCTG
GCCAACGGCGAGATCCGGAAGCGGCCTCTGATCGAGACAAACGGCGAAACCGGGGAGATCGTGTGGGATAAGGGCCGGGATTTTGCCACCGTGCGG
AAAGTGCTGAGCATGCCCCAAGTGAATATCGTGAAAAAGACCGAGGTGCAGACAGGCGGCTTCAGCAAAGAGTCTATCCTGCCCAAGAGGAACAGC
GATAAGCTGATCGCCAGAAAGAAGGACTGGGACCCTAAGAAGTACGGCGGCTTCGACAGCCCCACCGTGGCCTATTCTGTGCTGGTGGTGGCCAAA
GTGGAAAAGGGCAAGTCCAAGAAACTGAAGAGTGTGAAAGAGCTGCTGGGGATCACCATCATGGAAAGAAGCAGCTTCGAGAAGAATCCCATCGAC
TTTCTGGAAGCCAAGGGCTACAAAGAAGTGAAAAAGGACCTGATCATCAAGCTGCCTAAGTACTCCCTGTTCGAGCTGGAAAACGGCCGGAAGAGA
ATGCTGGCCTCTGCCGGCGAACTGCAGAAGGGAAACGAACTGGCCCTGCCCTCCAAATATGTGAACTTCCTGTACCTGGCCAGCCACTATGAGAAG
CTGAAGGGCTCCCCCGAGGATAATGAGCAGAAACAGCTGTTTGTGGAACAGCACAAGCACTACCTGGACGAGATCATCGAGCAGATCAGCGAGTTC
TCCAAGAGAGTGATCCTGGCCGACGCTAATCTGGACAAAGTGCTGTCCGCCTACAACAAGCACCGGGATAAGCCCATCAGAGAGCAGGCCGAGAAT
ATCATCCACCTGTTTACCCTGACCAATCTGGGAGCCCCTGCCGCCTTCAAGTACTTTGACACCACCATCGACCGGAAGAGGTACACCAGCACCAAA
GAGGTGCTGGACGCCACCCTGATCCACCAGAGCATCACCGGCCTGTACGAGACACGGATCGACCTGTCTCAGCTGGGAGGCGACCCAAAGAAGAAG
CGGAAGGTCTGAAAGCTTGCGGCCGCACTCGAGCACCACCACCACCACCACTGAGATCCGGCTGCTAACAAAGCCCGAAAGAGCGATTTCC
```

4. The merged sequence obtained via Fragment Merger and the merged sequence obtained via manual operation are aligned with EMBOSS needle. The sites that do not match are highlighted blue and shown as follows:

```
sequence           1 GCTGACTCTTCCCTCTAGAATAATTTTGTTTAACTTTAAGAAGGAGATAT     50
                     ||||||||||||||||||||||||||||||||||||||||||||||||||
gene               1 GCTGACTCTTCCCTCTAGAATAATTTTGTTTAACTTTAAGAAGGAGATAT     50

sequence          51 ACCATGGGCAGCAGCCATCATCATCATCATCACAGCAGCGGCCTGGAAGT    100
                     ||||||||||||||||||||||||||||||||||||||||||||||||||
gene              51 ACCATGGGCAGCAGCCATCATCATCATCATCACAGCAGCGGCCTGGAAGT    100

sequence         101 TCTGTTCCAGGGGCCCCATATGGCTAGCATGACTGGTGGACAGCAAATGG    150
                     ||||||||||||||||||||||||||||||||||||||||||||||||||
gene             101 TCTGTTCCAGGGGCCCCATATGGCTAGCATGACTGGTGGACAGCAAATGG    150

sequence         151 GTCGCGGATCCCCAAAGAAGAAGCGGAAGGTCGGTATCCACGGAGTCCCA    200
                     ||||||||||||||||||||||||||||||||||||||||||||||||||
gene             151 GTCGCGGATCCCCAAAGAAGAAGCGGAAGGTCGGTATCCACGGAGTCCCA    200

sequence         201 GCAGCCGACAAGAAGTACAGCATCGGCCTGGACATCGGCACCAACTCTGT    250
                     ||||||||||||||||||||||||||||||||||||||||||||||||||
gene             201 GCAGCCGACAAGAAGTACAGCATCGGCCTGGACATCGGCACCAACTCTGT    250

sequence         251 GGGCTGGGCCGTGATCACCGACGAGTACAAGGTGCCCAGCAAGAAATTCA    300
                     ||||||||||||||||||||||||||||||||||||||||||||||||||
gene             251 GGGCTGGGCCGTGATCACCGACGAGTACAAGGTGCCCAGCAAGAAATTCA    300

sequence         301 AGGTGCTGGGCAACACCGACCGGCACAGCATCAAGAAGAACCTGATCGGA    350
                     ||||||||||||||||||||||||||||||||||||||||||||||||||
gene             301 AGGTGCTGGGCAACACCGACCGGCACAGCATCAAGAAGAACCTGATCGGA    350

sequence         351 GCCCTGCTGTTCGACAGCGGCGAAACAGCCGAGGCCACCCGGCTGAAGAG    400
                     ||||||||||||||||||||||||||||||||||||||||||||||||||
gene             351 GCCCTGCTGTTCGACAGCGGCGAAACAGCCGAGGCCACCCGGCTGAAGAG    400

sequence         401 AACCGCCAGAAGAAGATACACCAGACGGAAGAACCGGATCTGCTATCTGC    450
                     ||||||||||||||||||||||||||||||||||||||||||||||||||
gene             401 AACCGCCAGAAGAAGATACACCAGACGGAAGAACCGGATCTGCTATCTGC    450

sequence         451 AAGAGATCTTCAGCAACGAGATGGCCAAGGTGGACGACAGCTTCTTCCAC    500
                     ||||||||||||||||||||||||||||||||||||||||||||||||||
gene             451 AAGAGATCTTCAGCAACGAGATGGCCAAGGTGGACGACAGCTTCTTCCAC    500

sequence         501 AGACTGGAAGAGTCCTTCCTGGTGGAAGAGGATAAGAAGCACGAGCGGCA    550
                     ||||||||||||||||||||||||||||||||||||||||||||||||||
gene             501 AGACTGGAAGAGTCCTTCCTGGTGGAAGAGGATAAGAAGCACGAGCGGCA    550
```

```
sequence         551 CCCCATCTTCGGCAACATCGTGGACGAGGTGGCCTACCACGAGAAGTACC    600
                     ||||||||||||||||||||||||||||||||||||||||||||||||||
gene             551 CCCCATCTTCGGCAACATCGTGGACGAGGTGGCCTACCACGAGAAGTACC    600

sequence         601 CCACCATCTACCACCTGAGAAAGAAACTGGTGGACAGCACCGACAAGGCC    650
                     ||||||||||||||||||||||||||||||||||||||||||||||||||
gene             601 CCACCATCTACCACCTGAGAAAGAAACTGGTGGACAGCACCGACAAGGCC    650

sequence         651 GACCTGCGGCTGATCTATCTGGCCCTGGCCCACATGATCAAGTTCCGGGG    700
                     ||||||||||||||||||||||||||||||||||||||||||||||||||
gene             651 GACCTGCGGCTGATCTATCTGGCCCTGGCCCACATGATCAAGTTCCGGGG    700

sequence         701 CCACTTCCTGATCGAGGGCGACCTGAACCCCGACAACAGCGACGTGGACA    750
                     ||||||||||||||||||||||||||||||||||||||||||||||||||
gene             701 CCACTTCCTGATCGAGGGCGACCTGAACCCCGACAACAGCGACGTGGACA    750

sequence         751 AGCTGTTCATCCAGCTGGTGCAGACCTACAACCAGCTGTTCGAGGAAAAC    800
                     ||||||||||||||||||||||||||||||||||||||||||||||||||
gene             751 AGCTGTTCATCCAGCTGGTGCAGACCTACAACCAGCTGTTCGAGGAAAAC    800

sequence         801 CCCATCAACGCCAGCGGCGTGGACGCCAAGGCCATCCTGTCTGCCAGACT    850
                     ||||||||||||||||||||||||||||||||||||||||||||||||||
gene             801 CCCATCAACGCCAGCGGCGTGGACGCCAAGGCCATCCTGTCTGCCAGACT    850

sequence         851 GAGCAAGAGCAGACGGCTGGAAAATCTGATCGCCCAGCTGCCCGGCGAGA    900
                     ||||||||||||||||||||||||||||||||||||||||||||||||||
gene             851 GAGCAAGAGCAGACGGCTGGAAAATCTGATCGCCCAGCTGCCCGGCGAGA    900

sequence         901 AGAAGAATGGCCCTGTTCGGAAACCTGATTGCCCTGAGCCTGGGCCTGAC    950
                     |||||||||| |||||||||||||||||||||||||||||||||||||||
gene             901 AGAAGAATGG-CCTGTTCGGAAACCTGATTGCCCTGAGCCTGGGCCTGAC    949

sequence         951 CCCCAACTTCAAGAGCAACTTCGACCTGGCCGAGGATGCCAAACTGCAGC   1000
                     ||||||||||||||||||||||||||||||||||||||||||||||||||
gene             950 CCCCAACTTCAAGAGCAACTTCGACCTGGCCGAGGATGCCAAACTGCAGC    999

sequence        1001 TGAGCAAGGACACCTACGACGACGACCT-GAC-ACCTGCTGGCCCAGATC   1048
                     |||||||||||||||||||||||||||| ||| |||||||||||||||||
gene            1000 TGAGCAAGGACACCTACGACGACGACCTGGACAACCTGCTGGCCCAGATC   1049

sequence        1049 GGCGACCAGTACGCCGACCTGTTTTCTATGC-----AGA--CTGT-CGAC   1090
                     ||||||||||||||||||||| ||||| .||     |||  |||| ||||
gene            1050 GGCGACCAGTACGCCGACCTG-TTTCT-GGCCGCCAAGAACCTGTCCGAC   1097

sequence        1091 G-CAT-CTGCTGA-CGACAT-CTGAGAGTGAACA-CGAGATCAC---AGC   1132
                     | ||| ||||||| |||||| ||||||||||||| |||||||||   .||
gene            1098 GCCATCCTGCTGAGCGACATCCTGAGAGTGAACACCGAGATCACCAAGGC   1147

sequence        1133 TCCCTTGA---CCT-TATGATCAGAGA-ATATCGATCGAGCACCTATC-G   1176
                     .|||.|||   ||| |||||||| ||| ||| ||| |||||||| |.| |
gene            1148 CCCCCTGAGCGCCTCTATGATCA-AGAGATA-CGA-CGAGCACC-ACCAG   1193

sequence        1177 AACCTGACCCTGCTGAAAGCTCTCGTGCGGCAGCAGCTGCCTGAGAAGTA   1226
                     .|||||||||||||||||||||||||||||||||||||||||||||||||
gene            1194 GACCTGACCCTGCTGAAAGCTCTCGTGCGGCAGCAGCTGCCTGAGAAGTA   1243

sequence        1227 CAAAGAGATTTTCTTCGACCAGAGCAAGAACGGCTACGCCGGCTACATTG   1276
                     ||||||||||||||||||||||||||||||||||||||||||||||||||
gene            1244 CAAAGAGATTTTCTTCGACCAGAGCAAGAACGGCTACGCCGGCTACATTG   1293

sequence        1277 ACGGCGGAGCCAGCCAGGAAGAGTTCTACAAGTTCATCAAGCCCATCCTG   1326
                     ||||||||||||||||||||||||||||||||||||||||||||||||||
gene            1294 ACGGCGGAGCCAGCCAGGAAGAGTTCTACAAGTTCATCAAGCCCATCCTG   1343

sequence        1327 GAAAAGATGGACGGCACCGAGGAACTGCTCGTGAAGCTGAACAGAGAGGA   1376
                     ||||||||||||||||||||||||||||||||||||||||||||||||||
gene            1344 GAAAAGATGGACGGCACCGAGGAACTGCTCGTGAAGCTGAACAGAGAGGA   1393

sequence        1377 CCTGCTGCGGAAGCAGCGGACCTTCGACAACGGCAGCATCCCCCACCAGA   1426
                     ||||||||||||||||||||||||||||||||||||||||||||||||||
gene            1394 CCTGCTGCGGAAGCAGCGGACCTTCGACAACGGCAGCATCCCCCACCAGA   1443

sequence        1427 TCCACCTGGGAGAGCTGCACGCCATTCTGCGGCGGCAGGAAGATTTTTAC   1476
                     ||||||||||||||||||||||||||||||||||||||||||||||||||
```

```
gene            1444 TCCACCTGGGAGAGCTGCACGCCATTCTGCGGCGGCAGGAAGATTTTTAC   1493

sequence        1477 CCATTCCTGAAGGACAACCGGGAAAAGATCGAGAAGATCCTGACCTTCCG   1526
                     ||||||||||||||||||||||||||||||||||||||||||||||||||
gene            1494 CCATTCCTGAAGGACAACCGGGAAAAGATCGAGAAGATCCTGACCTTCCG   1543

sequence        1527 CATCCCCTACTACGTGGGCCCTCTGGGCCAGGGGAAACAGCAGATTCGCC   1576
                     |||||||||||||||||||||||| |||||||||||||||||||||||||
gene            1544 CATCCCCTACTACGTGGGCCCTCT-GGCCAGGGGAAACAGCAGATTCGCC   1592

sequence        1577 TGGATGACCAG-AAGAGCGAGGAAACCATCACCCCCT-GCACTTCGAGGA   1624
                     ||||||||||| ||||||||||||||||||||||||| |.||||||||||
gene            1593 TGGATGACCAGAAAGAGCGAGGAAACCATCACCCCCTGGAACTTCGAGGA   1642

sequence        1625 AGT-GTGGACA--GGCGCT--CG-CCAGAGC-TCATCGAGC-GATGAC--   1664
                     ||| |||||||  ||||||  || ||||||| ||||||||| ||||||
gene            1643 AGTGGTGGACAAGGGCGCTTCCGCCCAGAGCTTCATCGAGCGGATGACCA   1692

sequence        1665 AC-TCGATAGACTGCCAACGAG-AGGTGCCCAACGAGAAGGTGCTGCCCA   1712
                     || ||||||           || |..||||||||||||||||||||||||
gene            1693 ACTTCGATA-----------AGAACCTGCCCAACGAGAAGGTGCTGCCCA   1731

sequence        1713 AGCACAGCCTGCTGTACGAGTACTTCACCGTGTATAACGAGCTGACCAAA   1762
                     ||||||||||||||||||||||||||||||||||||||||||||||||||
gene            1732 AGCACAGCCTGCTGTACGAGTACTTCACCGTGTATAACGAGCTGACCAAA   1781

sequence        1763 GTGAAATACGTGACCGAGGGAATGAGAAAGCCCGCCTTCCTGAGCGGCGA   1812
                     ||||||||||||||||||||||||||||||||||||||||||||||||||
gene            1782 GTGAAATACGTGACCGAGGGAATGAGAAAGCCCGCCTTCCTGAGCGGCGA   1831

sequence        1813 GCAGAAAAAGGCCATCGTGGACCTGCTGTTCAAGACCAACCGGAAAGTGA   1862
                     ||||||||||||||||||||||||||||||||||||||||||||||||||
gene            1832 GCAGAAAAAGGCCATCGTGGACCTGCTGTTCAAGACCAACCGGAAAGTGA   1881

sequence        1863 CCGTGAAGCAGCTGAAAGAGGACTACTTCAAGAAAATCGAGTGCTTCGAC   1912
                     ||||||||||||||||||||||||||||||||||||||||||||||||||
gene            1882 CCGTGAAGCAGCTGAAAGAGGACTACTTCAAGAAAATCGAGTGCTTCGAC   1931

sequence        1913 TCCGTGGAAATCTCCGGCGTGGAAGATCGGTTCAACGCCTCCCTGGGCAC   1962
                     ||||||||||||||||||||||||||||||||||||||||||||||||||
gene            1932 TCCGTGGAAATCTCCGGCGTGGAAGATCGGTTCAACGCCTCCCTGGGCAC   1981

sequence        1963 ATACCACGATCTGCTGAAAATTATCAAGGACAAGGACTTCCTGGACAATG   2012
                     ||||||||||||||||||||||||||||||||||||||||||||||||||
gene            1982 ATACCACGATCTGCTGAAAATTATCAAGGACAAGGACTTCCTGGACAATG   2031

sequence        2013 AGGAAAACGAGGACATTCTGGAAGATATCGTGCTGACCCTGACACTGTTT   2062
                     ||||||||||||||||||||||||||||||||||||||||||||||||||
gene            2032 AGGAAAACGAGGACATTCTGGAAGATATCGTGCTGACCCTGACACTGTTT   2081

sequence        2063 GAGGACAGAGAGATGATCGAGGAACGGCTGAAAACCTATGCCCACCTGTT   2112
                     ||||||||||||||||||||||||||||||||||||||||||||||||||
gene            2082 GAGGACAGAGAGATGATCGAGGAACGGCTGAAAACCTATGCCCACCTGTT   2131

sequence        2113 CGACGACAAAGTGATGAAGCAGCTGAAGCGGCGGAGATACACCGGCTGGG   2162
                     ||||||||||||||||||||||||||||||||||||||||||||||||||
gene            2132 CGACGACAAAGTGATGAAGCAGCTGAAGCGGCGGAGATACACCGGCTGGG   2181

sequence        2163 GCAGGCTGAGCCGGAAGCTGATCAACGGCATCCGGGACAAGCAGTCCGGG   2212
                     |||||||||||||||||||||||||||||||||||||||||||||||||.
gene            2182 GCAGGCTGAGCCGGAAGCTGATCAACGGCATCCGGGACAAGCAGTCCGGC   2231

sequence        2213 CAGAC-ATCCTGGATTTCCTGAAGTCCGACGGCTTCG-CAACAGAAAC-T   2259
                     .|||| ||||||||||| ||||||||||||||||||| |||||||||| |
gene            2232 AAGACAATCCTGGATTT-CTGAAGTCCGACGGCTTCGCCAACAGAAACTT   2280

sequence        2260 CATGCAGCTGAT-CACGACGACAGC-TGAC---TTAAGA-GACAT-CAG-   2301
                     |||||||||||| |||||||||||| ||||   |.|||| ||||| |||
gene            2281 CATGCAGCTGATCCACGACGACAGCGTGACCTTTAAAGAGGACATCCAGA   2330

sequence        2302 ATGCC---AGTGTC--GC--AGGCGATAG-CTGCACGAGCA-ATGGC--A   2340
                     |.|||   .|||||  ||  .|||||||| ||||||||||| ||.||  |
gene            2331 AAGCCCCAGGTGTCCGGCCAGGGCGATAGCCTGCACGAGCACATTGCCAA   2380

sequence        2341 TCTGTGCAGGCCG------CAT-----AGAGCAT-CTGC-GACTGAGTGT   2377
```

```
                             |||| ||.|||.|      |||     ||.|||| |||| |||  |||  
gene                    2381 TCTG-GCCGGCAGCCCCGCCATTAAGAAGGGCATCCTGCAGAC--AGT--   2425

sequence                2378 GACAGCTCTGAATGATGACCGCCAAGAAGGTGGTGGACGAGCTCGTGAAA   2427
                                                      |||||||||||||||||||||||||
gene                    2426 -------------------------GAAGGTGGTGGACGAGCTCGTGAAA   2450

sequence                2428 GTGATGGGCCGGCACAAGCCCGAGAACATCGTGATCGAAATGGCCAGAGA   2477
                             ||||||||||||||||||||||||||||||||||||||||||||||||||
gene                    2451 GTGATGGGCCGGCACAAGCCCGAGAACATCGTGATCGAAATGGCCAGAGA   2500

sequence                2478 GAACCAGACCACCCAGAAGGGACAGAAGAACAGCCGCGAGAGAATGAAGC   2527
                             ||||||||||||||||||||||||||||||||||||||||||||||||||
gene                    2501 GAACCAGACCACCCAGAAGGGACAGAAGAACAGCCGCGAGAGAATGAAGC   2550

sequence                2528 GGATCGAAGAGGGCATCAAAGAGCTGGGCAGCCAGATCCTGAAAGAACAC   2577
                             ||||||||||||||||||||||||||||||||||||||||||||||||||
gene                    2551 GGATCGAAGAGGGCATCAAAGAGCTGGGCAGCCAGATCCTGAAAGAACAC   2600

sequence                2578 CCCGTGGAAAACACCCAGCTGCAGAACGAGAAGCTGTACCTGTACTACCT   2627
                             ||||||||||||||||||||||||||||||||||||||||||||||||||
gene                    2601 CCCGTGGAAAACACCCAGCTGCAGAACGAGAAGCTGTACCTGTACTACCT   2650

sequence                2628 GCAGAATGGGCGGGATATGTACGTGGACCAGGAACTGGACATCAACCGGC   2677
                             ||||||||||||||||||||||||||||||||||||||||||||||||||
gene                    2651 GCAGAATGGGCGGGATATGTACGTGGACCAGGAACTGGACATCAACCGGC   2700

sequence                2678 TGTCCGACTACGATGTGGACCATATCGTGCCTCAGAGCTTTCTGAAGGAC   2727
                             ||||||||||||||||||||||||||||||||||||||||||||||||||
gene                    2701 TGTCCGACTACGATGTGGACCATATCGTGCCTCAGAGCTTTCTGAAGGAC   2750

sequence                2728 GACTCCATCGACAACAAAGTGCTGACCAGAAGCGACAAGAACCGGGGCAA   2777
                             |||||||||||||||||.||||||||||||||||||||||||||||||||
gene                    2751 GACTCCATCGACAACAAGGTGCTGACCAGAAGCGACAAGAACCGGGGCAA   2800

sequence                2778 GAGCGACAACGTGCCCTCCGAAGAGGTCGTGAAGAAGATGAAGAACTACT   2827
                             ||||||||||||||||||||||||||||||||||||||||||||||||||
gene                    2801 GAGCGACAACGTGCCCTCCGAAGAGGTCGTGAAGAAGATGAAGAACTACT   2850

sequence                2828 GGCGGCAGCTGCTGAACGCCCAAGCTGATTACCCAGAGAAAGTTCGAC-A   2876
                             |||||||||||||||||| ||||||||||||||||||||||||||||| |
gene                    2851 GGCGGCAGCTGCTGAACG-CCAAGCTGATTACCCAGAGAAAGTTCGACAA   2899

sequence                2877 TCTGACCAACG-CCGAGAGAGGCGGCCTGAGCGAACTGGATAATGC---G   2922
                             |||||||||.| ||||||||||||| |||||||||||||||||.||   |
gene                    2900 TCTGACCAAGGCCCGAGAGAGGCGG-CTGAGCGAACTGGATAAGGCCCGG   2948

sequence                2923 CTTCATCAGGAGACAGCTGATG--AACCCCGCAGATCACAAAGCACGTGG   2970
                             ||||||||.||||||||||.||  |||||.||||||||||||||||||||
gene                    2949 CTTCATCAAGAGACAGCTGGTGGAAACCCGGCAGATCACAAAGCACGTGG   2998

sequence                2971 CACAGATC--TGACTCCCGGATG-ACACTTAGTACGACGAG-ATGACAGC   3016
                             ||||||||  .|||||||||||| |||||.||||||||||| ||||||..
gene                    2999 CACAGATCCTGGACTCCCGGATGAACACTAAGTACGACGAGAATGACAAG   3048

sequence                3017 CTGATC--GGCAGTG-AAGTGATCACCCTTGAGTTC-AGCTTGGGT-TCC   3061
                             ||||||  ||.|||| |||||||||||||..|||.| ||||  ||| |||
gene                    3049 CTGATCCGGGAAGTGAAAGTGATCACCCTGAAGTCCAAGCT--GGTGTCC   3096

sequence                3062 GATT--CGCAAAGAATTTCCAGTTTTACAAAGTGCGCGAGATCAACAACT   3109
                             ||||  || .|||.||||||||||||||||||||||||||||||||||||
gene                    3097 GATTTCCG-GAAGGATTTCCAGTTTTACAAAGTGCGCGAGATCAACAACT   3145

sequence                3110 ACCACCACGCCCACGACGCCTACCTGAACGCCGTCGTGGGAACCGCCCTG   3159
                             ||||||||||||||||||||||||||||||||||||||||||||||||||
gene                    3146 ACCACCACGCCCACGACGCCTACCTGAACGCCGTCGTGGGAACCGCCCTG   3195

sequence                3160 ATCAAAAAGTACCCTAAGCTGGAAAGCGAGTTCGTGTACGGCGACTACAA   3209
                             ||||||||||||||||||||||||||||||||||||||||||||||||||
gene                    3196 ATCAAAAAGTACCCTAAGCTGGAAAGCGAGTTCGTGTACGGCGACTACAA   3245

sequence                3210 GGTGTACGACGTGCGGAAGATGATCGCCAAGAGCGAGCAGGAAATCGGCA   3259
                             ||||||||||||||||||||||||||||||||||||||||||||||||||
gene                    3246 GGTGTACGACGTGCGGAAGATGATCGCCAAGAGCGAGCAGGAAATCGGCA   3295
```

```
sequence        3260 AGGCTACCGCCAAGTACTTCTTCTACAGCAACATCATGAACTTTTTCAAG   3309
                     ||||||||||||||||||||||||||||||||||||||||||||||||||
gene            3296 AGGCTACCGCCAAGTACTTCTTCTACAGCAACATCATGAACTTTTTCAAG   3345

sequence        3310 ACCGAGATTACCCTGGCCAACGGCGAGATCCGGAAGCGGCCTCTGATCGA   3359
                     ||||||||||||||||||||||||||||||||||||||||||||||||||
gene            3346 ACCGAGATTACCCTGGCCAACGGCGAGATCCGGAAGCGGCCTCTGATCGA   3395

sequence        3360 GACAAACGGCGAAACCGGGGAGATCGTGTGGGATAAGGGCCGGGATTTTG   3409
                     ||||||||||||||||||||||||||||||||||||||||||||||||||
gene            3396 GACAAACGGCGAAACCGGGGAGATCGTGTGGGATAAGGGCCGGGATTTTG   3445

sequence        3410 CCACCGTGCGGAAAGTGCTGAGCATGCCCCAAGTGAATATCGTGAAAAAG   3459
                     ||||||||||||||||||||||||||||||||||||||||||||||||||
gene            3446 CCACCGTGCGGAAAGTGCTGAGCATGCCCCAAGTGAATATCGTGAAAAAG   3495

sequence        3460 ACCGAGGTGCAGACAGGCGGCTTCAGCAAAGAGTCTATCCTGCCCAAGAG   3509
                     ||||||||||||||||||||||||||||||||||||||||||||||||||
gene            3496 ACCGAGGTGCAGACAGGCGGCTTCAGCAAAGAGTCTATCCTGCCCAAGAG   3545

sequence        3510 GAACAGCGATAAGCTGATCGCCAGAAAGAAGGACTGGGACCCTAAGAAGT   3559
                     ||||||||||||||||||||||||||||||||||||||||||||||||||
gene            3546 GAACAGCGATAAGCTGATCGCCAGAAAGAAGGACTGGGACCCTAAGAAGT   3595

sequence        3560 ACGGCGGCTTCGACAGCCCCACCGTGGCCTATTCTGTGCTGGTGGTGGCC   3609
                     ||||||||||||||||||||||||||||||||||||||||||||||||||
gene            3596 ACGGCGGCTTCGACAGCCCCACCGTGGCCTATTCTGTGCTGGTGGTGGCC   3645

sequence        3610 AAAGTGGAAAAGGGCAAGTCCAAGAAACTGAAGAGTGTGAAAGAGCTGCT   3659
                     ||||||||||||||||||||||||||||||||||||||||||||||||||
gene            3646 AAAGTGGAAAAGGGCAAGTCCAAGAAACTGAAGAGTGTGAAAGAGCTGCT   3695

sequence        3660 GGGGATCACCATCATGGAAAGAAGCAGCTTCGAGAAGAATCCCATCGAC-   3708
                     ||||||||||||||||||||||||||||||||||||||||||||||||| 
gene            3696 GGGGATCACCATCATGGAAAGAAGCAGCTTCGAGAAGAATCCCATCGACT   3745

sequence        3709 TTCT-GAAGCCAAGGGCTACAAAGAAGTGAAAAAGGACCTGATCATCAAG   3757
                     |||| |||||||||||||||||||||||||||||||||||||||||||||
gene            3746 TTCTGGAAGCCAAGGGCTACAAAGAAGTGAAAAAGGACCTGATCATCAAG   3795

sequence        3758 CTGCCTAAGTACTCCCTGTTCGAGCTGGAAAACGGCCGGAAGAGAATGCT   3807
                     ||||||||||||||||||||||||||||||||||||||||||||||||||
gene            3796 CTGCCTAAGTACTCCCTGTTCGAGCTGGAAAACGGCCGGAAGAGAATGCT   3845

sequence        3808 GGCCTCTGCCGGCGAACTGCAGAAGGGAAACGAACTGGCCCTGCCCTCCA   3857
                     ||||||||||||||||||||||||||||||||||||||||||||||||||
gene            3846 GGCCTCTGCCGGCGAACTGCAGAAGGGAAACGAACTGGCCCTGCCCTCCA   3895

sequence        3858 AATATGTGAACTTCCTGTACCTGGCCAGCCACTATGAGAAGCTGAAGGGC   3907
                     ||||||||||||||||||||||||||||||||||||||||||||||||||
gene            3896 AATATGTGAACTTCCTGTACCTGGCCAGCCACTATGAGAAGCTGAAGGGC   3945

sequence        3908 TCCCCCGAGGATAATGAGCAGAAACAGCTGTTTGTGGAACAGCACAAGCA   3957
                     ||||||||||||||||||||||||||||||||||||||||||||||||||
gene            3946 TCCCCCGAGGATAATGAGCAGAAACAGCTGTTTGTGGAACAGCACAAGCA   3995

sequence        3958 CTACCTGGACGAGATCATCGAGCAGATCAGCGAGTTCTCCAAGAGAGTGA   4007
                     ||||||||||||||||||||||||||||||||||||||||||||||||||
gene            3996 CTACCTGGACGAGATCATCGAGCAGATCAGCGAGTTCTCCAAGAGAGTGA   4045

sequence        4008 TCCTGGCCGACGCTAATCTGGACAAAGTGCTGTCCGCCTACAACAAGCAC   4057
                     ||||||||||||||||||||||||||||||||||||||||||||||||||
gene            4046 TCCTGGCCGACGCTAATCTGGACAAAGTGCTGTCCGCCTACAACAAGCAC   4095

sequence        4058 CGGGATAAGCCCATCAGAGAGCAGGCCGAGAATATCATCCACCTGTTTAC   4107
                     ||||||||||||||||||||||||||||||||||||||||||||||||||
gene            4096 CGGGATAAGCCCATCAGAGAGCAGGCCGAGAATATCATCCACCTGTTTAC   4145

sequence        4108 CCTGACCAATCTGGGAGCCCCTGCCGCCTTCAAGTACTTTGACACCACCA   4157
                     ||||||||||||||||||||||||||||||||||||||||||||||||||
gene            4146 CCTGACCAATCTGGGAGCCCCTGCCGCCTTCAAGTACTTTGACACCACCA   4195

sequence        4158 TCGACCGGAAGAGGTACACCAGCACCAAAGAGGTGCTGGACGCCACCCTG   4207
                     ||||||||||||||||||||||||||||||||||||||||||||||||||
gene            4196 TCGACCGGAAGAGGTACACCAGCACCAAAGAGGTGCTGGACGCCACCCTG   4245
```

```
sequence        4208 ATCCACCAGAGCATCACCGGCCTGTACGAGACACGGATCGACCTGTCTCA   4257
                     ||||||||||||||||||||||||||||||||||||||||||||||||||
gene            4246 ATCCACCAGAGCATCACCGGCCTGTACGAGACACGGATCGACCTGTCTCA   4295

sequence        4258 GCTGGGAGGCGACCCAAAGAAGAAGCGGAAGGTCTGAAAGCTTGCGGCCG   4307
                     ||||||||||||||||||||||||||||||||||||||||||||||||||
gene            4296 GCTGGGAGGCGACCCAAAGAAGAAGCGGAAGGTCTGAAAGCTTGCGGCCG   4345

sequence        4308 CACTCGAGCACCACCACCACCACCACTGAGATCCGGCTGCTAACAAAGCC   4357
                     ||||||||||||||||||||||||||||||||||||||||||||||||||
gene            4346 CACTCGAGCACCACCACCACCACCACTGAGATCCGGCTGCTAACAAAGCC   4395

sequence        4358 CGAAAGAGCGATTTCC   4373
                     ||||||||||||||||
gene            4396 CGAAAGAGCGATTTCC   4411
```
