# Supplementary material for: A Python script to merge Sanger sequences
Source: PeerJ. 2021 Apr 27;9:e11354. doi: 10.7717/peerj.11354 (PMC8086567; doi:10.7717/peerj.11354)
Supplement: Supplemental Information 12 [file peerj-09-11354-s012.docx]

**How to run the script to merge the Sanger sequences**

1. Make a new directory and copy the Sanger sequencing result files to the newly made directory. Also, copy the script ‘Merge_Sanger_v2.py’ to the directory.

2. Open a terminal, cd to the directory, input command like this and press enter

python3.7 Merging_sequencing_v2.py 000F-original_file_name1.seq 001F-original_file_name2.seq 002R-original_file_name3.seq 003R-original_file_name4.seq

3. After running finishes, a folder named ‘merged_sequence, which contains the merged sequence file with the name ‘merged.seq’, appears. The terminal screen also prints the useful information for knowing what the script has done.

Please notice the followings:

(1) The script requires macOS Catalina 10.15.3 or higher (Apple Inc., CA, USA), Python 3.7.3, Biopython 1.7.4 and EMBOSS 6.6.0., please install the three packages in advance before running the script (see the references in the manuscript for installation).

(2) This script is used to merge multiple successive Sanger DNA sequencing results. It can merge 2 to hundreds of tandemly arranged Sanger sequencing results. Please notice that a space key between each input item is obligatory, and the tab key can be used to automatically fill up the input file name to simplify the input process. Also, the backward slash "\" can be used a multiline command input like this:

python3.7 Merge_Sanger_v2.py 001F_sequence_to_be_merged.seq \

002F_sequence_to_be_merged.seq 003F_sequence_to_be_merged.seq \

004R_sequence_to_be_merged.seq 005R_sequence_to_be_merged.seq \

006R_sequence_to_be_merged.seq

(3) The file names of the sequences to be merged starts with '000' plus 'F' (forward sequencing result file) or 'R' (reverse sequencing result file), and are in .seq format. This can be done by adding the four characters to the original file name provided by the sequencing company. The files are arranged in tandemly according to their real positions corresponding to the sequencing plasmid or linear DNA.
